# Adaptive Evolution of the Fox Coronavirus Based on Genome-Wide Sequence Analysis

**DOI:** 10.1155/2022/9627961

**Published:** 2022-04-13

**Authors:** Chunyu Feng, Yuting Liu, Guangqi Lyu, Songyang Shang, Hongyue Xia, Junpeng Zhang, David M. Irwin, Zhe Wang, Shuyi Zhang

**Affiliations:** ^1^College of Animal Science and Veterinary Medicine, Shenyang Agricultural University, Shenyang 110866, China; ^2^Agricultural Development Service Center of Hunnan District, Shenyang 110000, China; ^3^Department of Laboratory Medicine and Pathobiology, University of Toronto, Toronto, Ontario, Canada M5S 1A8

## Abstract

**Purpose:**

To report the first complete fox coronavirus (CoV) genome sequence obtained through genome-wide amplifications and to understand the adaptive evolution of fox CoV.

**Methods:**

Anal swab samples were collected from 35 foxes to detect the presence of CoV and obtain the virus sequence. Phylogenetic analysis was conducted using MrBayes. The possibility of recombination within these sequences was assessed using GARD. Analysis of the levels of selection pressure experienced by these sequences was assessed using methods on both the PAML and Data Monkey platforms.

**Results:**

Of the 35 samples, two were positive, and complete genome sequences for the viruses were obtained. Phylogenetic analysis, using Bayesian methods, of these sequences, together with other CoV sequences, revealed that the fox CoV sequences clustered with canine coronavirus (CCoV) sequences, with sequences from other carnivores more distantly related. In contrast to the feline, ferret and mink CoV sequences that clustered into species-specific clades, the fox CoV fell within the CCoV clade. Minimal evidence for recombination was found among the sequences. A total of 7, 3, 14, and 2 positively selected sites were identified in the M, N, S, and 7B genes, respectively, with 99, 111, and 581 negatively selected sites identified in M, N, and S genes, respectively.

**Conclusion:**

The complete genome sequence of fox CoV has been obtained for the first time. The results suggest that the genome sequence of fox CoV may have experienced adaptive evolution in the genes replication, entry, and virulence. The number of sites in each gene that experienced negative selection is far greater than the number that underwent positive selection, suggesting that most of the sequence is highly conserved and important for viral survive. However, positive selection at a few sites likely aided these viruses to adapt to new environments.

## 1. Introduction

Coronavirus (CoV) are widely found in nature and belong to the order *Nidovirales*, within the subfamily *Coronavirinae* of the *family Coronaviridae*. These viruses are single-stranded positive-sense RNA viruses with a capsule membrane [1, 2] and possess the longest RNA viruses with a genome that is between 27 and 31 kb in length [3]. CoV are classified as *Alphacoronavirus*, *Betacoronavirus*, *Gammacoronavirus*, and *Deltacoronavirus* based to their serotypes and genotypes [4–6] and can infect birds and mammals. These viruses are capable of transmission between species, perhaps due to their high rates of mutation and recombination. Over the past decade, several CoVs of animal origin have spread among humans, including severe acute respiratory syndrome coronavirus (SARS-CoV), which caused more than 8,000 cases and at least 700 deaths [7–9]; Middle East respiratory syndrome (MERS-CoV), a zoonotic pathogen that continues to infect humans [10–13]; and SARS-CoV-2, which broke out in such a violent manner that it quickly spreads across the world with serious consequences [14–16]. Therefore, it is very important to study the CoVs that are carried by animals.

In 1971, canine coronavirus (CCoV) was first reported in Germany, with subsequent reports in Asia, other parts of Europe, South America, North America, and other countries and regions [17]. Today, CCoV is widespread across the world and causes serious harm to the dog industry. The first report on CCoV in China was by Xu et al. [18], while Hu et al. [19] reported some of the characteristics of a CCoV that was isolated from the liver of a giant panda. Wang et al. [20] reported the prevalence of CCoV in the feces of dogs, foxes, raccoons, and minks in China using a reverse transcription polymerase chain reaction (RT-PCR) assay to detect this virus. This study showed that CCoV is prevalent in healthy dogs, foxes, raccoons, and minks in China [20]. Ma et al. [21] reported that CCoV belonging to the HC2, HF3, and HR strains could be isolated from dogs, foxes, and raccoons, and used these partial sequences to carry out a series of analyses and phylogenetic studies. While comparisons using gene segments provide important insights into evolutionary trends and the pathogenicity of these CoV, they could improve with complete genome sequences. To our knowledge, the CCoV complete genome sequences are available from other canines, but no complete genome sequence of a fox CoV is currently available.

In this study, the whole-genome sequence of the fox CoV was amplified by RT-PCR and nested RT-PCR. Phylogenetic, recombination, and selective level analyses were performed using our new whole-genome sequences together with other CoV sequences including feline coronavirus (FCoV), CCoV, and ferret CoV, to yield insight into evolutionary trends and the molecular basis of the pathogenicity of this virus.

## 2. Materials and Methods

### 2.1. Animal Sampling

All procedures used here were approved by the Ethics Committee for Animal Experiments of Shenyang Agricultural University. Anal swab samples were collected from foxes kept at the Shenyang Animal Rescue and Support Center between October and November 2019, yielding a total of 35 samples. Samples were collected using animal virus sampling tubes from Youkang Hengye Biotechnology (Beijing) Co., Ltd., temporary stored on ice, and then returned to the laboratory the same day. In the laboratory, samples were divided into 1.5-ml centrifuge tubes and stored at -80°C.

### 2.2. Detection of Viral RNA

Viral RNA, to be used as template for cDNA synthesis, was extracted from each of the 35 samples using the TaKaRa MiniBEST Viral RNA/DNA Extraction Kit Ver.5.0 (Axygen Company), according to the manufacturer's instructions. Viral cDNA synthesis was carried out according to the instructions of the PrimeScript II 1st Strand cDNA Synthesis Kit (Dalian). Synthesized cDNA was stored at -20°C until used as template for PCR. Fox CoV was detected by RT-PCR using primers (forward: N-F: 5′-GATCTCAATCTAGAGGAAGG-3′; Reverse: N-R: 5′-GTTTGATGACACACAGGTTG-3′) reported by Lu et al. [22] that amplify a 606-bp fragment of the N gene. PCR amplification was performed in a 10 *μ*l reaction volume, composing 11 *μ*l of each primer, 1 *μ*l cDNA, 2 *μ*l double-distilled water, and 5 *μ*l 2× EasyTaq PCR SuperMix (Takara Biotechnology Co., Ltd., People's Republic of China). PCR conditions consisted of an initial activation step of 95°C for 5 minutes followed by 35 cycles of denaturation at 95°C for 30 seconds, annealing at 55°C for 30 seconds, extension at 72°C for 1 minute, and finally, extend it at 72°C for 10 minutes.

### 2.3. Genome Sequence Amplification

Primers suitable for the amplification of the fox CoV genome were initially based on those reported by Lu et al. [22], with additional primers designed from the sequences generated by our results. Primers were designed using Primer Premier 5 and synthesized by Shanghai Sangon Biotechnology Co., Ltd. Sequences and the locations of the primers are shown in Table S1. We used a combination of PCR and nested PCR to generate the complete genome sequence. The first round of PCR included an initial denaturation step of 95°C for 5 minutes followed by 35 cycles of denaturation at 95°C for 30 seconds, an annealing temperature that depended on the primer pair for 30 seconds and an extension at 72°C for a time that depended on the length of the amplification product, and finally an extension at 72°C for 10 minutes. Conditions for the subsequent rounds of PCR were similar to the first round, except for the different temperatures used during annealing and the extension times at 72°C.

### 2.4. Sequence Alignment and Phylogenetic Analysis

In addition to the fox CoV sequence, we obtained, by sequencing in this study, additional CoV sequence that are closely related to fox CoV (e.g., CCoV, FCoV, and ferret CoV) that were downloaded from the GenBank database. Information on these sequences is provided in Table S2.

To establish the phylogenetic relationships of these viruses, we first used ClustalW, from molecular evolutionary genetics analysis software Mega 6 [23], to generate an alignment of the viral genome sequences. A Bayesian phylogenetic tree was constructed using MrBayes 3.1 [24] based on the aligned nucleotide sequences, with 5,000,000 generations, sampled every 100 generations, using the commands mcmcp samplefreq =100, a burnin of 20,000 generations, 4 chains, the “allcompat” option was used for generating consensus tree, and the best fit substitution model GTR + I + G applied, with the best fit substitution model selected using jModelTest [25]. After checking convergence between the two Bayesian runs by Tracer [26], we discarded as aging the first one (nucleotide data) or two (amino acid data) million generations before summarizing the results as a consensus tree. Porcine epidemic diarrhea virus (PEDV) sequences (accession numbers NC_003436 and LT897799.1) were used as the outgroup to root the trees.

### 2.5. Recombination Analysis

Since recombination can influence the detection of positive selection, we tested our alignment for the presence of recombination using GARD (genetic algorithm for recombination detection) from Data Monkey suite of programs [27]. A model selection procedure was run for each gene (the E, M, N, S, and 7b genes were each considered to be a single gene), which tests 203 possible time reversible models in a hierarchical testing procedure combining nested LRT (likelihood ratio test) tests with AIC selection to identify a single “best-fitting” rate matrix, with site-to-site rate variation accounted for by the *β*–*Γ* distribution [27]. A KH (Kishino-Hasegawa) test was used to evaluate the significance, and the *p* values on both sides of the potential breakpoints were evaluated that LHS (left-hand side) and RHS (right-hand side) represent the *p* values left and right of the breakpoint, respectively. If the *p* values on both sides of a breakpoint were less than 0.05, then a breakpoint is inferred.

### 2.6. Selective Pressure Analysis

To measure the level of selection acting on the sequences, we used branch, site, and branch-site tests from the PAML package [28]. *ω* (the nonsynonymous/synonymous rate ratio) values greater than 1 suggest positive selection. *p* values can be calculated through an LRT; if the *p* value of the calculated result is less than 0.05, then the null hypothesis (neutral evolution) is rejected. The branch model detects positive selection in a particular lineage [29, 30]. We analyzed three models: one ratio, free ratio, and two ratio. Comparing the free ratio and the one ratio models tests whether the ratio is different among different lineages. A comparison of the two ratios and free ratio tests establishes whether difference exists between the foreground and background systems studied. The sites model test allows different *ω* ratios at sites in the alignment [31, 32]. The different models (M0, M1, M2, M3, M7, and M8) were tested by adjusting the parameters such as Nssites. Among these models, LRT comparisons of the M1 and M2 and the M7 and M8 model pairs test for positive selection, while comparison of M0 and M3 is used to detect the diversity of *ω* values across sequence sites. If a site class with *ω* greater than one is found, which suggests positive selection, then sites with evidence of positive selection and a posterior probability *p* >95% level were identified. Posterior probabilities were calculated by NEB (naïve empirical Bayes) and the BEB (Bayes empirical Bayes) [31].

In addition, we also used several methods from the Data Monkey suite of programs [27] to detect the presence of positive selection in our alignment, including FEL (fixed-effects likelihood; *p* < 0.05), REL (random effects likelihood; Bayes factor>50), and MEME (mixed-effects model of evolution; *p* < 0.05). Sites that were identified as potentially selected by methods from both PAML and Data Monkey were concluded to have experienced positive or negative selection.

## 3. Results

### 3.1. Genome Sequence

We tested samples from 35 foxes and found that two of them (fox 38 and 41) were positive for CoV sequences; both foxes infected with these viruses were found to have diarrhea and lethargy. Complete genome sequences were then generated for both of these fox samples, which we named fox CoV-38 and fox CoV-41. The two sequences were 29,598 bp and 29,851 bp in length and deposited into GenBank with accession numbers MW354910 and MW354911, respectively. The lengths of our fox CoV sequences are in agreement with the coronavirus genome sequence lengths, which range from 27 to 31 kb. Both genome sequences were found to contain the genes 2a, 2b, S, E, M, N, 7a, and 7b. The GC content of the fox CoV-38 and fox CoV-41 genome sequences are 37.59% and 37.49%, respectively. Through three ways, sequence alignment, recombination analysis, and selection pressure analysis, it is concluded that sequences fox CoV-38 and -41 belong to the same type, with BLAST comparisons also confirming that fox CoV is similar to CCoV.

### 3.2. Sequence Alignment and Phylogenetic Analysis

**Figure 1:**
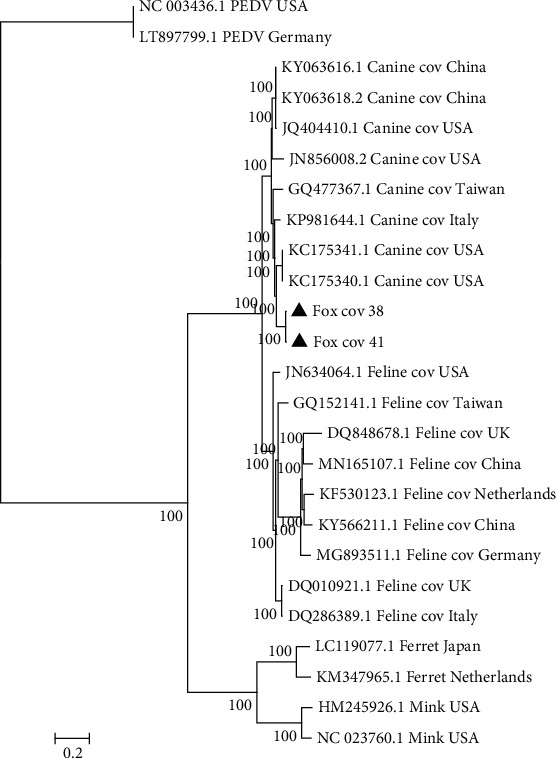
Phylogenetic relationships of carnivore CoV sequences. Phylogenetic relationships of 25 complete CoV genome sequences from foxes, dogs and cats, mink and ferret inferred using MrBayes 3.1. The posterior probabilities for each node are shown and the PEDV sequences were used as the outgroup. Strains identified in this study are labeled with triangles. Branches are proportional to the amount of inferred change, with the bar representing 0.2 nucleotide substitutions per site.

To better understand the evolution of the fox CoV, a phylogenetic analysis was conducted. Our phylogenetic analysis was performed using complete genome sequences and included sequences from other 25 CoV genomes found in carnivores and used PEDV sequences for rooting. Our analysis showed that our two fox CoV genomes clustered, forming a unique branch (Figure 1). Species-specific CoV clusters were found for the other species (dog, cat, mink, and ferret), with the fox CoV most closely related to CCoV (Figure 1). Mink and ferret CoV were most distantly related, with FCoV having an intermediate relationship.

### 3.3. Analysis for Recombination

As recombination can mislead tests for positive selection, we first used GARD [27] to test for the presence of recombination breakpoints in our genome alignment (Table 1). GARD detected one recombination breakpoint in the M gene and three recombination breakpoints in both the S and N genes; however, only the sites in the M gene (site 252) and the S gene (sites 949, 1193, and 3022) remained significant (*p* < 0.05) after the application of the KH test with Bonferroni correction.

### 3.4. Analysis of Selective Pressure

When the branch and branch site models of PAML were applied, no evidence for positive selection was found. To identify sites with evidence for positive selection, we applied five methods to our aligned CoV sequences. The results from these five methods are shown in Table 2. The identification of positive selection sites in PAML was performed when the *p* value of M7 VS M8 was less than 0.05 in a LRT. In the M gene, a total of seven sites (57, 59, 61, 62, 66, 77, and 263) were identified (*P* < 0.05) by at least one method, with sites 59 and 77 being detected by methods from both PAML and Data Monkey. For the N gene, three sites (32, 45, and 320) were identified, all of which were only detected by programs from the Data Monkey suite. The S gene has three sites (25, 36, and 194) that were detected using methods from both PAML and Data Monkey, with eight sites (13, 18, 22, 61, 188, 196, 225, and 276) detected using only PAML and three sites (28, 397, and 1400) detected only using Data Monkey. For the 7B gene, positive selection was detected at two sites (82 and 104) using both Data Monkey.

In addition to detecting positive selection, the FEL and REL methods from Data Monkey also detect sites experiencing negative selection. These methods found 99, 111, and 581 sites experiencing negative selection in the M, N, and S genes, respectively, with these sites showing significance with both methods (Tables S3-S8).

## 4. Discussion

We obtained full length genome sequences for two fox CoVs. To our knowledge, this is the first time that a whole-genome sequence of a fox CoV has been reported [20, 21]. This result provides important resources for studying evolution trends of this virus in foxes. In this study, a phylogenetic study, using Bayesian methods, was conducted using the two whole-genome sequences for fox CoV obtained here together with other CoVVs (e.g., FCoV, CCoV, and ferret CoV) that are closely related to fox CoV to explore the evolutionary changes in these coronaviruses. The clustering of CoV sequences from these foxes within the dog CCoV sequences is consistent with the close relationship between foxes and dogs [33] and also suggests that these viruses can be transmitted between these two species. The FCoVs forms separate distinct cluster, as does a cluster that contains both ferret and mink CoVs, yet maintaining species specificity, suggesting that the CoVs retain some degree of species specificity. Intriguingly, our fox CoV sequences do not cluster with CCoV from China in our phylogenetic tree (Figure 1), but rather are more closely related to CCoV from other countries, thus suggesting that the fox samples identified here are not recent infections from dogs in China. The absence of significant regional clustering of the CoV sequences could also be due to the international trade in canids and increased tourism, where the virus can be transmitted from one country to another.

We used programs from the Data Monkey suite to identify codons in the CoV gene sequences that display evidence of selection and to detect the presence of recombination within the sequences [34]. GARD was used first to detect recombination breakpoints in genes [30], resulting in the identification of three breakpoints in the S gene and one in the M gene fragment, after *p* values were corrected by Bonferroni correction. As the number of breakpoints was not more than 3, it has been suggested that this should have little influence on empirical Bayesian methods to detect positive selection sites [35]. In addition, the S gene of CCoV is highly similar to HCOV-OC43 and bovine coronavirus (BCoV) [36]; type II FCoV occurs through the recombination of type I FCoV and type II CCoV [37, 38]. Previous studies have shown that the transmission of naturally occurring recombinant strains occurs only rarely; thus, we should be cautious about extrapolating recombination events solely based on these analyses [39].

Adaptive evolution of viruses occurs via both positive and negative selection. Positive selection leads to an increase in the number of genetic mutations, while negative selection tends to conserve gene sequences [40–42]. Since the results of using only one analysis method are relatively partial (Tables S3-S5), we combined the intersection of the results analyzed by these methods to determine the occurrence of positive selection sites to ensure the reliability of the analysis results. When we carried out site selection pressure analyses, we identified 7 sites in M gene, 3 sites in N gene, 14 sites in S gene, and 2 sites in 7B gene that had evidence for positive selection. By analyzing the selection pressures experienced by these genes in the genome, we identified sites that are potentially experiencing adaptive evolution. In other words, these sites in fox CoV have also experienced adaptive evolution. The FEL and REL programs from the Data Monkey suite can identify sites in genes experiencing negative selection. Results from showed that FEL and REL identified 99, 111, and 581 negative selection sites in M, N and S genes, respectively (Tables S6-S11). As previously reported, positive selection has been detected at site 245 of the S gene, the site that distinguishes FIPV from FECV in 96% of cases; it is suggested that the positively selected site 245 may be associated with the occurrence of FIPV [43]. In this study, we detected 14 positive selection sites in the S gene and speculated that the occurrence of fox CoV may be related to this. The detection of sites experiencing positive selection in these genes suggests that they contribute to the survival and adaptation of these viruses. Positive selection at sites in the S gene likely contributes to the wide host range of this virus [44–47], while positive selection at other sites may contribute to the entry of viruses into host cells and enhance their virulence. Among all of the genes that we analyzed, a much larger number of negative selection sites were detected than positive selection sites, suggesting that most sites are conserved; positive selection of a small number of sites may help these viruses adapt to survive under harmful conditions.

## 5. Conclusion

The complete genome sequence of fox CoV has been obtained for the first time. The results suggest that the genome sequence of fox CoV may have experienced adaptive evolution in the genes replication, entry, and virulence. The number of sites in each gene that experienced negative selection is far greater than the number that underwent positive selection, suggesting that most of the sequence is highly conserved and important for viral survive. However, positive selection at a few sites likely aided these viruses adapt to new environments.

## Figures and Tables

**Table 1:** KH Breakpoints testing using the GARD method.

				*p* value (Bonferroni corrected)	
Gene	Number	△AICc	Location	LHS	RHS
S	3	3722.71	949	0.0024 (0.0144)	0.0006 (0.0036)
			1193	0.0006 (0.0036)	0.0006 (0.0036)
			3022	0.0006 (0.0036)	0.0006 (0.0036)
M	1	316.14	252	0.0002 (0.0004)	0.0002 (0.0004)
N	3	179.27	353	0.0648 (0.3888)	0.0168 (0.0504)
			838	0.0006 (0.0036)	0.0198 (0.1188)
			1108	0.0972 (0.5832)	0.0174 (0.1044)

Breakpoint estimation and KH test were performed using the nucleotide sequence alignment. All *p* values are adjusted by Bonferroni correction.

**Table 2:** Sites with evidence of positive selection in the M, N, S, and 7b gene.

Gene	Position	FEL p^a^	REL bf^b^	MEME p^a^	M3 NEB pp^c^	M8 BEB pp^c^
M	57	—	—	—	0.988	0.990
	59	—	—	0.047	0.995	0.995
	61	—	—	—	0.989	0.990
	62	—	—	—	1.000	1.000
	66	—	—	—	0.957	0.961
	77	0.024	122.241	—	0.990	0.990
	263	0.033	—	0.020	—	—
N	32	0.020	70.121	0.009	—	—
	45	—	81.452	0.048	—	—
	320	0.007	343.486	0.003	—	—
S	13	—	—	—	0.993	0.994
	18	—	—	—	0.983	0.987
	22	—	—	—	0.950	0.966
	25	—	—	0.043	0.998	0.998
	28	0.044	—	0.029	—	—
	36	—	—	0.013	0.989	0.990
	61	—	—	—	0.997	0.997
	188	—	—	—	0.999	0.999
	194	0.006	—	0.004	—	0.964
	196	—	—	—	0.954	0.966
	225	—	—	—	0.976	0.982
	276	—	—	—	0.998	0.998
	397	0.049	—	0.027	—	—
	1400	—	160.429	0.041	—	—
7b	82	0.008	—	0.023	—	—
	104	0.045	—	0.004	—	—

^a^ *p* value, ^b^Bayes factor, ^c^Posterior probability. “–” represents negative results of positive selection.

## Data Availability

All virus data obtained or analyzed during this study are included in this published article. Sequences of the obtained viruses have been uploaded in GenBank with accession numbers is MW354910 and MW354911.

## Supplementary Materials

Supplementary Materials The supplementary material lists information on other coronavirus sequences used in this study, primers designed for the amplification of genomic sequences, and the negative selection sites in the M, N, and S genes identified by the analyses with REL and FEL.
