# Supplementary material for: Adaptive Evolution of the Fox Coronavirus Based on Genome-Wide Sequence Analysis
Source: Biomed Res Int. 2022 Apr 13;2022:9627961. doi: 10.1155/2022/9627961 (PMC9020971; doi:10.1155/2022/9627961)
Supplement: Supplementary Materials — The supplementary material lists information on other coronavirus sequences used in this study, primers designed for the amplification of genomic sequences, and the negative selection sites in the M, N, and S genes identified by the analyses with REL and FEL. [file 9627961.f1.docx]

**Table S1** Viral genome-wide segmented amplification primers.

| Primer | Sequence (5‘→ 3’) | Product size (bp) | Annealing temperature （℃） |
| --- | --- | --- | --- |
| 1F1 | ATACTACTGGTGACAAGTCTGAAC | 1710 | 60 |
| 1R1 | GGAAACCATAATGMGCTTGC |  |  |
| 1F2 | CGAAGAAGAAGTTGAATCTGTTGAAG | 1662 | 60 |
| 1R2 | AACCATAATGMGCTTGCCAATC |  |  |
| 1HF1 | CTTGYTGCTAAGTGTGGTTGTG | 549 | 61 |
| 1HR1 | GATCAAGACATCCAAATCACCCT |  |  |
| 1HF2 | CTTGYTGCTAAGTGTGGTTGTGG | 548 | 62 |
| 1HR2 | ATCAAGACATCCAAATCACCCTG |  |  |
| 12F1 | CTTGAAACATCATTGCAGTGCTTAC | 1249 | 62 |
| 12R1 | GCGGTGGTTTTACTTTAACTTTAGG |  |  |
| 12F2 | CAGAGAATTGCATGTCTTCGTGTAC | 1206 | 61 |
| 12R2 | GGTGGTTTTACTTTAACTTTAGGCATC |  |  |
| 2F1 | CTGTTGGGTTAATGCAATTTGTC | 2041 | 60 |
| 2R1 | TGACACATCAAGATTGCAAGCC |  |  |
| 2F2 | CTCAATGGAAATTTCCTGGTGTTAG | 1986 | 62 |
| 2R2 | GATTGCAAGCCTTGTAGCATTCA |  |  |
| 23F1 | TARATCTCACCAAGAAGTCACAAAAG | 1587 | 61 |
| 23R1 | GCTACYACAGACATGTTAGAGTTAAAGAG |  |  |
| 23F2 | ACAGATTCTATGTTGGTGATGAGTTC | 1474 | 61 |
| 23R2 | CCACAGATATATCCATTGCCAAACT |  |  |
| 3F1 | CTGGCAATGCAGTGAGTAAGG | 1361 | 58 |
| 3R1 | GTGTTCTGGTGTGTTAGGATTGAC |  |  |
| 3F2 | ACGCTGCATGCACCACTCTTAC | 1300 | 61 |
| 3R2 | TTAGGATTGACCTGGTTAACTTTGAG |  |  |
| 34F1 | GCCTAGTGGTCTTGTAGAGCCTTG | 1037 | 61 |
| 34R1 | CCGTTGAGACYAAAGTTGTGAC |  |  |
| 34F2 | CCTATGGTAACAATGTGCTTAATGG | 997 | 61 |
| 34R2 | GTTGAGACYAAAGTTGTGACAGCCAG |  |  |
| 4F1 | CAAATGTATGGTGTAAGTCTTCAAGC | 2160 | 61 |
| 4R1 | CAACGTATGCTTTAGCAGGATCAG |  |  |
| 4F2 | ATGTATGGTGTAAGTCTTCAAGCTGG | 2154 | 61 |
| 4R2 | CGTATGCTTTAGCAGGATCAGGT |  |  |
| 5F1 | ACCTTAAGTATGTYAAATGGGAGAGC | 2318 | 61 |
| 5R1 | CAATTGACTTCAAATGCTTCTGGT |  |  |
| 5F2 | GCGYTAATGGTCCTGAAGTCAAG | 2209 | 61 |
| 5R2 | TTGTCKCGTAGTCATAGTAGAAAGAAGTG |  |  |
| 6F1 | ATGCATTAATGCTCGTGAAGTTG | 2138 | 59 |
| 6R1 | CMAGTGTRTTATAGGCTTCCGC |  |  |
| 6F2 | GTTACAAACTATGACAAGAGTGCTGG | 2066 | 60 |
| 6R2 | GATATGGTATTGTACTTTTCTTGGTTGAC |  |  |
| 7F1 | CGGCCCTAAGGAAATTGTACTC | 2445 | 60 |
| 7R1 | AGACCATTCATTTGACCATGTACC |  |  |
| 7F2 | TGAGCAATCTGAGTACGGTAGTGAC | 2084 | 61 |
| 7R2 | GGTACACAATAGACCAATCAACACG |  |  |
| 8F1 | GCTGGTGGTCTTGAGCTTACAACTATG | 2336 | 64 |
| 8R1 | CGTCGGAAACGTAATCTCTCAAGTC |  |  |
| 8F2 | CGGCAAAGYTGCAACTTGTTATAG | 2235 | 63 |
| 8R2 | CCAATATGGCATCATCTGGTAACC |  |  |
| 9F1 | CACAGCTCCAATCTGCCGAATG | 2130 | 66 |
| 9R1 | CATAACAGTATCGCGGTCCGTCAG |  |  |
| 9F2 | CATGCCTACATTGTACAAAATCCAGC | 2072 | 65 |
| 9R2 | CAGTTACGCCGAATGGAATCTCAC |  |  |
| 10F1 | GTTTTGAAGGTGCCCAGTTTAGCC | 2710 | 65 |
| 10R1 | AACACTGTGTGAAAGAAGATCATGCC |  |  |
| 10F2 | GAAGGTGCCCAGTTTAGCCAATG | 2567 | 65 |
| 10R2 | CTAGCCCTAACCTCYGCTTGTCTGG |  |  |
| 11F1 | CAACAGATTCTGGCTAATGCTTTC | 2013 | 60 |
| 11R1 | GGAGAGACCAAGCTTAGCATGTG |  |  |
| 11F2 | GCCATACATCAAACATCACAAGGTC | 1940 | 61 |
| 11R2 | GAGAGACCAAGCTTAGCATGTGC |  |  |
| 12F1 | GGTCTTGTATGGACATTGTCAAATCTAT | 1912 | 62 |
| 12R1 | GTGTGTAGACGATGGTCCTGCC |  |  |
| 12F2 | CTATTGACACATCCGTAGACGCTG | 1836 | 62 |
| 12R2 | ATTGTCGATGTTCATACCACCAGC |  |  |
| 13F1 | AGTTTCCAGATATGTAATGTTCGGC | 1624 | 62 |
| 13R1 | GTTCGTCACCTCATCAATAATCTCAA |  |  |
| 13F2 | TTCGGCTTTAGTGTTGCAGGTG | 1528 | 62 |
| 13R2 | CAACCTCTTGCTCTGACTTATCTGC |  |  |
| 14F1 | GCCATTGGACTGCAAAGGAAG | 1301 | 62 |
| 14R1 | GTACAAGAGTAGACAGCGCGATGA |  |  |
| 14F2 | GGAAGATGGTGACCAGATTGAGG | 1274 | 62 |
| 14R2 | GACAGCGCGATGACCAGTAATT |  |  |

**Table S2** Other coronavirus sequences used in the study.

| No. | Accession No. | Sequence | Source | Collection date |
| --- | --- | --- | --- | --- |
| 1 | KY063616.1 | Complete genome | Canine coronavirus | Jul-2016 |
| 2 | KY063618.2 | Complete genome | Canine coronavirus | Jul-2016 |
| 3 | KP981644.1 | Complete genome | Canine coronavirus | 2005 |
| 4 | KC175341.1 | Complete genome | Canine coronavirus | 1978 |
| 5 | KC175340.1 | Complete genome | Canine coronavirus | 1978 |
| 6 | JQ404410.1 | Complete genome | Canine coronavirus | 2012 |
| 7 | JN856008.2 | Complete genome | Canine coronavirus | 1976 |
| 8 | GQ477367.1 | Complete genome | Canine coronavirus | Nov-2008 |
| 9 | MN165107.1 | Complete genome | Feline coronavirus | Apr-2018 |
| 10 | JN634064.1 | Complete genome | Feline coronavirus | 2011 |
| 11 | GQ152141.1 | Complete genome | Feline coronavirus | Sep-2007 |
| 12 | MG893511.1 | Complete genome | Feline coronavirus | Jul-2012 |
| 13 | DQ848678.1 | Complete genome | Feline coronavirus | 2006 |
| 14 | DQ010921.1 | Complete genome | Feline coronavirus | 2005 |
| 15 | KF530123.1 | Complete genome | Feline coronavirus | Aug-2010 |
| 16 | DQ286389.1 | Complete genome | Feline coronavirus | 2005 |
| 17 | KY566211.1 | Complete genome | Feline coronavirus | Nov-2016 |
| 18 | LC119077.1 | Complete genome | Ferret coronavirus | Jun-2013 |
| 19 | KM347965.1 | Complete genome | Ferret coronavirus | 2010 |
| 20 | NC_023760.1 | Complete genome | Mink coronavirus | Jan-1998 |
| 21 | HM245926.1 | Complete genome | Mink coronavirus | Jan-1998 |
| 22 | NC_003436 | Complete genome | PEDV | 2000 |
| 23 | LT897799.1 | Complete genome | PEDV | 2017 |
| 24 | NC_028814.1 | Complete genome | Bat coronavirus | 2013 |
| 25 | NC_010437.1 | Complete genome | Bat coronavirus | 2008 |
| 26 | KU740200.1 | Complete genome | MERS CoV | 2014 |
| 27 | KT225476.2 | Complete genome | MERS CoV | 2015 |
| 28 | NC_004718.3 | Complete genome | SARS CoV | 2003 |
| 29 | KF514421.1 | Complete genome | SARS CoV | 2010 |
| 30 | NC_045512.2 | Complete genome | SARS CoV2 | 2020 |
| 31 | MT419818.1 | Complete genome | SARS CoV2 | 2020 |
| 32 | MN988668.1 | Complete genome | SARS CoV2 | 2020 |
| 33 | MW031801.1 | Complete genome | SARS CoV2 | 2020 |

**Table S3** Calculation results of FEL.

| Gene | Site | dN/dS | Normalized dN-dS | P-value |
| --- | --- | --- | --- | --- |
| E | **-** | **-** | **-** | **-** |
| M | 77 | 1154461611324262.750 | 2.25 | 0.02 |
|  | 263 | Infinite | 0.47 | 0.03 |
| N | 32 | Infinite | 2.57 | 0.02 |
|  | 320 | 11.806 | 2.45 | 0.01 |
| S | 28 | 1959429095550303.750 | 1.19 | 0.04 |
|  | 194 | 77018900.000 | 22.77 | 0.01 |
|  | 397 | Infinite | 0.48 | 0.05 |
|  | 482 | Infinite | 0.56 | 0.03 |
|  | 717 | Infinite | 0.28 | 0.04 |
| 7b | 22 | 697.879 | 1.91 | 0.01 |
|  | 74 | 4715262840918868.000 | 1.38 | 0.03 |
|  | 82 | Infinite | 0.77 | 0.01 |
|  | 104 | 71.409 | 1.67 | 0.05 |

**Table S4** Calculation results of REL.

| Gene | Site | Normalized dN-dS | Posterior Probability | Bayes Factor |
| --- | --- | --- | --- | --- |
| E |  | -0.805 | 0.592 |  |
| M | 33 | 0.89 | 0.91 | 297.51 |
|  | 77 | 0.79 | 0.81 | 122.24 |
|  | 148 | 0.55 | 0.64 | 53.12 |
| N | 32 | 0.14 | 0.72 | 70.12 |
|  | 45 | 0.16 | 0.75 | 81.45 |
|  | 320 | 0.30 | 0.93 | 343.49 |
|  | 363 | 0.15 | 0.76 | 86.70 |
|  | 419 | 0.15 | 0.83 | 144.04 |
| S | 202 | 0.08 | 0.86 | 94.73 |
|  | 453 | 0.05 | 0.81 | 68.15 |
|  | 1400 | 0.11 | 0.91 | 160.43 |
| **7b** |  | -0.74 | 1.428 |  |

**Table S5** Calculation results of MEME.

| Gene | Site | dS | dN^-^ | p1 | dN^+^ | p2 | p-value | q-value |
| --- | --- | --- | --- | --- | --- | --- | --- | --- |
| E | 33 | 0.35 | 0 | 0.90 | 49.35 | 0.10 | 0.02 | 1 |
| M | 35 | 0.47 | 0 | 0.97 | 57.14 | 0.03 | 0.02 | 1 |
|  | 59 | 0.65 | 0.65 | 0.79 | 159.05 | 0.21 | 0.05 | 1 |
|  | 60 | 1.49 | 0 | 0.74 | 14.82 | 0.26 | 0.00 | 0.52 |
|  | 76 | 0.65 | 0.58 | 0.86 | 1012.61 | 0.14 | 0.01 | 0.91 |
|  | 88 | 2.03 | 0 | 0.97 | 190.01 | 0.03 | 0.04 | 1 |
|  | 250 | 0.31 | 0.06 | 0.92 | 14.56 | 0.08 | 0.00 | 0.58 |
|  | 263 | 0 | 0 | 0.78 | 2.45 | 0.21 | 0.02 | 1 |
| N | 20 | 0 | 0 | 0.97 | 326.16 | 0.03 | 0.00 | 0.75 |
|  | 32 | 0.10 | 0.10 | 0.82 | 29.75 | 0.17 | 0.01 | 0.82 |
|  | 45 | 0 | 0 | 0.73 | 7.02 | 0.27 | 0.05 | 1 |
|  | 77 | 0.81 | 0 | 0.97 | 87.25 | 0.03 | 0.01 | 0.81 |
|  | 176 | 0.31 | 0 | 0.77 | 5.31 | 0.23 | 0.04 | 1 |
|  | 225 | 0.15 | 0 | 0.82 | 4.06 | 0.17 | 0.00 | 1 |
|  | 320 | 0.24 | 0.12 | 0.88 | 40.11 | 0.12 | 0.00 | 0.74 |
|  | 431 | 0 | 0 | 0.87 | 3.32 | 0.13 | 0.03 | 1 |
| S | 25 | 0.81 | 0.12 | 0.48 | 12.39 | 0.51 | 0.04 | 1 |
|  | 28 | 0 | 0 | 0.00 | 2.68 | 1 | 0.03 | 1 |
|  | 36 | 0.27 | 0.27 | 0.72 | 17.41 | 0.23 | 0.01 | 0.97 |
|  | 47 | 0.73 | 0.04 | 0.90 | 14.18 | 0.10 | 0.04 | 1 |
|  | 64 | 0.30 | 0.03 | 0.97 | 13.71 | 0.03 | 0.02 | 1 |
|  | 65 | 0.25 | 0.10 | 0.93 | 51.24 | 0.07 | 0.00 | 0.55 |
|  | 123 | 0.22 | 0 | 0.85 | 27.36 | 0.15 | 0.01 | 1 |
|  | 153 | 0 | 0 | 0.79 | 8.54 | 0.21 | 0.01 | 1 |
|  | 168 | 0.33 | 0.23 | 0.73 | 9.61 | 0.27 | 0.04 | 1 |
|  | 180 | 0.62 | 0.11 | 0.97 | 177.81 | 0.03 | 0.04 | 1 |
|  | 194 | 1.30 | 0.50 | 0.78 | 425.64 | 0.22 | 0.00 | 1 |
|  | 201 | 0.05 | 0 | 0.65 | 5.32 | 0.35 | 0.01 | 0.99 |
|  | 253 | 2.63 | 0.15 | 0.93 | 858.19 | 0.07 | 0.01 | 0.93 |
|  | 255 | 0.07 | 0.07 | 0.69 | 21.15 | 0.31 | 0.01 | 0.98 |
|  | 269 | 0.99 | 0.20 | 0.91 | 119.87 | 0.09 | 0.01 | 0.86 |
|  | 286 | 0.14 | 0 | 0.90 | 12.6 | 0.10 | 0.04 | 1 |
|  | 318 | 0 | 0 | 0.03 | 0.81 | 0.97 | 0.03 | 1 |
|  | 323 | 9.41 | 0 | 0.92 | 1185.28 | 0.08 | 0.03 | 1 |
|  | 395 | 0.30 | 0.03 | 0.94 | 22.06 | 0.06 | 0.03 | 1 |
|  | 397 | 0 | 0 | 0.48 | 1.63 | 0.52 | 0.03 | 1 |
|  | 410 | 0 | 0 | 0.94 | 49.73 | 0.06 | 0.00 | 0.00 |
|  | 418 | 1.55 | 0.21 | 0.95 | 104.21 | 0.05 | 0.02 | 1 |
|  | 420 | 0.36 | 0 | 0.94 | 61.55 | 0.06 | 0.03 | 1 |
|  | 428 | 0.56 | 0 | 0.95 | 131.71 | 0.05 | 0.00 | 0.42 |
|  | 530 | 0.20 | 0.09 | 0.91 | 6.54 | 0.09 | 0.01 | 1 |
|  | 580 | 0.21 | 0 | 0.86 | 56.55 | 0.14 | 0.00 | 0.18 |
|  | 611 | 0.85 | 0.23 | 0.88 | 25.61 | 0.12 | 0.04 | 1 |
|  | 676 | 0.16 | 0 | 0.83 | 4.39 | 0.17 | 0.01 | 0.88 |
|  | 685 | 0.65 | 0.10 | 0.70 | 39.23 | 0.30 | 0.04 | 1 |
|  | 688 | 0.89 | 0.76 | 0.94 | 336.91 | 0.06 | 0.02 | 1 |
|  | 694 | 0.15 | 0.03 | 0.91 | 69.55 | 0.09 | 0.01 | 0.91 |
|  | 728 | 0 | 0 | 0.74 | 20.99 | 0.26 | 0.01 | 0.97 |
|  | 775 | 0.86 | 0 | 0.94 | 63.47 | 0.06 | 0.03 | 1 |
|  | 926 | 0.10 | 0.03 | 0.97 | 152.66 | 0.03 | 0.00 | 0.54 |
|  | 948 | 0.59 | 0.06 | 0.94 | 81.54 | 0.06 | 0.03 | 1 |
|  | 963 | 0 | 0 | 0.94 | 12.07 | 0.06 | 0.01 | 0.99 |
|  | 1056 | 0.20 | 0.20 | 0.79 | 21.34 | 0.21 | 0.04 | 1 |
|  | 1057 | 0.27 | 0 | 0.76 | 20.03 | 0.24 | 0.01 | 0.90 |
|  | 1079 | 1.23 | 0.10 | 0.96 | 24.78 | 0.04 | 0.02 | 1 |
|  | 1392 | 0.35 | 0.04 | 0.91 | 39.13 | 0.09 | 0.01 | 1 |
|  | 1400 | 0.28 | 0.14 | 0.65 | 3.60 | 0.35 | 0.04 | 1 |
|  | 1420 | 0.51 | 0.01 | 0.96 | 227.00 | 0.04 | 0.03 | 1 |
|  | 1465 | 0.91 | 0.08 | 0.97 | 272.44 | 0.03 | 0.00 | 0.21 |
|  | 1469 | 0.77 | 0 | 0.97 | 511.52 | 0.03 | 0.01 | 0.96 |
|  | 1482 | 0.15 | 0 | 0.81 | 2.45 | 0.19 | 0.04 | 1 |
|  | 1489 | 0 | 0 | 0.85 | 1.85 | 0.15 | 0.04 | 1 |
| **7b** | 7 | 0.14 | 0 | 0.88 | 21.22 | 0.12 | 0.00 | 0.31 |
|  | 12 | 0.95 | 0.26 | 0.95 | 34.11 | 0.05 | 0.03 | 0.75 |
|  | 50 | 0 | 0 | 0.89 | 6.34 | 0.11 | 0.04 | 0.93 |
|  | 61 | 1.17 | 0 | 0.87 | 319.99 | 0.13 | 0.01 | 0.71 |
|  | 82 | 0 | 0 | 0.69 | 2.85 | 0.31 | 0.02 | 0.72 |
|  | 104 | 0 | 0 | 0.73 | 12.51 | 0.27 | 0.00 | 0.41 |
|  | 154 | 0.05 | 0.05 | 0.89 | 5.99 | 0.11 | 0.02 | 0.63 |
|  | 159 | 0 | 0 | 0.76 | 4.51 | 0.24 | 0.02 | 0.69 |
|  | 166 | 0 | 0 | 0.82 | 1.76 | 0.18 | 0.05 | 0.91 |
|  | 168 | 0 | 0 | 0.86 | 3.01 | 0.14 | 0.01 | 0.91 |
|  | 210 | 0.22 | 0 | 0.90 | 5.92 | 0.10 | 0.03 | 0.69 |

**Table S6** Negative selection sites for the *M* gene based on FEL analysis.

| Codon | dS | dN | dN Leaves | dN/dS | Normalized dN-dS | p-value | |
| --- | --- | --- | --- | --- | --- | --- | --- |
| 27 | 31.5973 | 0.236574 | 0 | 0.007 | -15.3799 | 0.0401517 |  |
| 44 | 15.3937 | 0.53599 | 0.273038 | 0.035 | -7.2865 | 0.0133596 |  |
| 51 | 8.48191 | 0.334393 | 0.376785 | 0.039 | -3.9957 | 0.000374579 |  |
| 52 | 6.19475 | 0 | 0 | 0.000 | -3.03803 | 0.000261184 |  |
| 53 | 2.0968 | 0 | 0 | 0.000 | -1.02831 | 0.0155748 |  |
| 54 | 3.16105 | 0.623405 | 0.349655 | 0.197 | -1.24451 | 0.0440561 |  |
| 55 | 99.1466 | 0.17469 | 0 | 0.002 | -48.5378 | 0.00361561 |  |
| 65 | 4.59809 | 0.501105 | 0 | 0.109 | -2.00925 | 0.0114936 |  |
| 71 | 3.70128 | 0 | 0.637 | 0.000 | -1.81518 | 0.000727403 |  |
| 73 | 13.0702 | 0.475808 | 0.885348 | 0.036 | -6.17654 | 0.00117833 |  |
| 78 | 9.16623 | 0 | 0.80403 | 0.000 | -4.4953 | 0.0211961 |  |
| 88 | 2.26589 | 0 | 0.311878 | 0.000 | -1.11124 | 0.00210594 |  |
| 90 | 1.36153 | 0 | 0 | 0.000 | -0.667721 | 0.00669745 |  |
| 91 | 2.26024 | 0 | 0 | 0.000 | -1.10847 | 0.000913779 |  |
| 95 | 0.877013 | 0 | 0.269905 | 0.000 | -0.430105 | 0.0108173 |  |
| 102 | 1.94414 | 0 | 0 | 0.000 | -0.953447 | 0.00291695 |  |
| 103 | 1.35609 | 0.150948 | 0 | 0.111 | -0.591024 | 0.0448807 |  |
| 108 | 2.64692 | 0.181862 | 0 | 0.069 | -1.20891 | 0.00942157 |  |
| 109 | 2.70781 | 0 | 0 | 0.000 | -1.32796 | 0.000442118 |  |
| 112 | 1.58536 | 0 | 0 | 0.000 | -0.777494 | 0.00178981 |  |
| 114 | 3.67679 | 0.303145 | 0 | 0.082 | -1.6545 | 0.00215905 |  |
| 116 | 3.88735 | 0.327823 | 0 | 0.084 | -1.74567 | 0.0215674 |  |
| 122 | 1.04928 | 0 | 0 | 0.000 | -0.514586 | 0.00601321 |  |
| 126 | 2.77365 | 0.389492 | 0 | 0.140 | -1.16924 | 0.012282 |  |
| 127 | 0.846976 | 0 | 0 | 0.000 | -0.415374 | 0.0118698 |  |
| 131 | 2.81303 | 0 | 0.485308 | 0.000 | -1.37957 | 0.00057692 |  |
| 133 | 0.616927 | 0 | 0 | 0.000 | -0.302553 | 0.0303166 |  |
| 135 | 1.16258 | 0 | 0 | 0.000 | -0.570154 | 0.00696093 |  |
| 136 | 2.25253 | 0 | 0 | 0.000 | -1.10469 | 0.000535456 |  |
| 137 | 1.6786 | 0 | 0 | 0.000 | -0.823219 | 0.000949835 |  |
| 143 | 0.537691 | 0 | 0 | 0.000 | -0.263694 | 0.0383424 |  |
| 145 | 3.00095 | 0.174533 | 0.75306 | 0.058 | -1.38613 | 0.00468682 |  |
| 147 | 5.80519 | 0.159539 | 0.35923 | 0.027 | -2.76874 | 0.000527848 |  |
| 158 | 1.29032 | 0 | 0 | 0.000 | -0.6328 | 0.0079605 |  |
| 165 | 0.840466 | 0 | 0 | 0.000 | -0.412181 | 0.00965196 |  |
| 168 | 3.06012 | 0.180599 | 0 | 0.059 | -1.41218 | 0.00685731 |  |
| 174 | 2.31905 | 0.162518 | 0.365785 | 0.070 | -1.05761 | 0.00944324 |  |
| 179 | 1.52984 | 0 | 0 | 0.000 | -0.750265 | 0.00268409 |  |
| 181 | 1.67673 | 0 | 0.317283 | 0.000 | -0.822302 | 0.00317203 |  |
| 182 | 2.1511 | 0 | 0 | 0.000 | -1.05494 | 0.00116236 |  |
| 183 | 0.715408 | 0 | 0 | 0.000 | -0.350851 | 0.0135018 |  |
| 188 | 0.666515 | 0 | 0 | 0.000 | -0.326873 | 0.033681 |  |
| 189 | 1.61702 | 0 | 0 | 0.000 | -0.793018 | 0.00354568 |  |
| 190 | 1.03699 | 0 | 0 | 0.000 | -0.508561 | 0.00513194 |  |
| 192 | 11.3349 | 0 | 0 | 0.000 | -5.55887 | 4.67135e-07 |  |
| 193 | 1.22609 | 0 | 0 | 0.000 | -0.6013 | 0.00593311 |  |
| 194 | 2.61997 | 0.115788 | 0 | 0.044 | -1.2281 | 0.00380508 |  |
| 195 | 0.944413 | 0 | 0 | 0.000 | -0.463159 | 0.00725294 |  |
| 197 | 1.4632 | 0 | 0 | 0.000 | -0.717585 | 0.00191243 |  |
| 201 | 2.30768 | 0.310652 | 0.324807 | 0.135 | -0.979383 | 0.0265251 |  |
| 203 | 1.22996 | 0 | 0 | 0.000 | -0.603199 | 0.00449692 |  |
| 205 | 2.8042 | 0.352737 | 0.619049 | 0.126 | -1.20225 | 0.0225121 |  |
| 206 | 26.9082 | 0.433507 | 0 | 0.016 | -12.9837 | 0.000833266 |  |
| 207 | 2.6989 | 0.445794 | 0 | 0.165 | -1.10497 | 0.0277844 |  |
| 208 | 7.27531 | 0.200203 | 0 | 0.028 | -3.46978 | 0.000133816 |  |
| 209 | 3.5563 | 0 | 0 | 0.000 | -1.74408 | 2.24263e-05 |  |
| 212 | 2.47352 | 0 | 0 | 0.000 | -1.21307 | 0.000293672 |  |
| 214 | 1.4647 | 0 | 0 | 0.000 | -0.71832 | 0.00211709 |  |
| 215 | 2.77626 | 0 | 0 | 0.000 | -1.36153 | 0.000126636 |  |
| 216 | 0.614024 | 0 | 0 | 0.000 | -0.30113 | 0.0385004 |  |
| 217 | 2.6582 | 0.157362 | 0 | 0.059 | -1.22646 | 0.00376922 |  |
| 218 | 0.855618 | 0 | 0 | 0.000 | -0.419612 | 0.00964233 |  |
| 219 | 3.06141 | 0 | 0 | 0.000 | -1.50138 | 0.000177649 |  |
| 220 | 5.74543 | 0 | 0 | 0.000 | -2.81768 | 2.29829e-06 |  |
| 221 | 3.08189 | 0 | 0 | 0.000 | -1.51142 | 0.00017836 |  |
| 222 | 1.59809 | 0 | 0 | 0.000 | -0.783737 | 0.00166773 |  |
| 224 | 0.691165 | 0 | 0 | 0.000 | -0.338961 | 0.0204922 |  |
| 226 | 1.10591 | 0 | 0 | 0.000 | -0.542362 | 0.00804689 |  |
| 229 | 1.06657 | 0 | 0 | 0.000 | -0.523069 | 0.0111259 |  |
| 230 | 7.99593 | 0 | 0 | 0.000 | -3.92137 | 1.20649e-06 |  |
| 231 | 3.62887 | 0.147559 | 0 | 0.041 | -1.70731 | 0.00153673 |  |
| 232 | 1.03695 | 0 | 0 | 0.000 | -0.508541 | 0.0119082 |  |
| 236 | 0.93074 | 0 | 0 | 0.000 | -0.456454 | 0.0112443 |  |
| 237 | 12.1178 | 0.403613 | 0 | 0.033 | -5.74488 | 0.00672908 |  |
| 239 | 1.78695 | 0.169189 | 0 | 0.095 | -0.79338 | 0.0167283 |  |
| 242 | 4.60164 | 0 | 0 | 0.000 | -2.25674 | 2.72861e-05 |  |
| 243 | 0.621406 | 0 | 0 | 0.000 | -0.30475 | 0.0293244 |  |
| 244 | 3.17679 | 0.121291 | 0 | 0.038 | -1.49848 | 0.00440336 |  |
| 245 | 6.85973 | 0.158614 | 0 | 0.023 | -3.28636 | 0.000123865 |  |
| 249 | 0.955904 | 0 | 0 | 0.000 | -0.468795 | 0.00823807 |  |
| 253 | 2.99763 | 0.513675 | 0 | 0.171 | -1.21818 | 0.0444053 |  |
| 254 | 0.429978 | 0 | 0 | 0.000 | -0.21087 | 0.0484647 |  |
| 255 | 1.03371 | 0 | 0 | 0.000 | -0.506953 | 0.00709185 |  |
| 256 | 1.46758 | 0 | 0 | 0.000 | -0.71973 | 0.00261169 |  |
| 257 | 0.525838 | 0 | 0 | 0.000 | -0.257881 | 0.047937 |  |
| 260 | 0.700507 | 0 | 0 | 0.000 | -0.343543 | 0.0347413 |  |
| 261 | 9.12577 | 0 | 0 | 0.000 | -4.47546 | 4.19571e-07 |  |
| 262 | 3.49524 | 0.14235 | 0 | 0.041 | -1.64433 | 0.0157303 |  |
| 265 | 2.18329 | 0.120353 | 0 | 0.055 | -1.0117 | 0.0283147 |  |
| 266 | 0.555279 | 0 | 0.33312 | 0.000 | -0.27232 | 0.0346487 |  |
| 269 | 2.27559 | 0.366863 | 0 | 0.161 | -0.93608 | 0.0412853 |  |
| 270 | 1.42679 | 0 | 0 | 0.000 | -0.699727 | 0.00185194 |  |
| 276 | 0.83431 | 0 | 0 | 0.000 | -0.409162 | 0.0148643 |  |
| 280 | 3.44824 | 0.367992 | 0 | 0.107 | -1.51062 | 0.008272 |  |
| 288 | 3.10651 | 0.322883 | 0 | 0.104 | -1.36514 | 0.0363959 |  |
| 290 | 4.47753 | 0.251249 | 0 | 0.056 | -2.07266 | 0.00221585 |  |
| 295 | 1.60989 | 0.122128 | 0 | 0.076 | -0.729627 | 0.0393013 |  |
| 297 | 1.06712 | 0 | 0 | 0.000 | -0.523337 | 0.0111564 |  |
| 303 | 0.578399 | 0 | 0 | 0.000 | -0.283658 | 0.0397539 |  |

**Table S7** Negative selection sites for the *M* gene based on REL analysis.

| Codon | E[dS] | E[dN] | Normalized E[dN-dS] | Posterior Probability | Bayes Factor |
| --- | --- | --- | --- | --- | --- |
| 27 | 1.60497 | 0.130732 | -1.47424 | 0.999942 | 592.808 |
| 44 | 2.70465 | 0.1865 | -2.51815 | 0.999996 | 7869.98 |
| 51 | 3.92859 | 0.179832 | -3.74876 | 0.999999 | 60258.5 |
| 52 | 1.98106 | 0.0505691 | -1.93049 | 1 | 4.84703e+06 |
| 53 | 1.17045 | 0.0502829 | -1.12016 | 1 | 261937 |
| 54 | 1.5092 | 0.189066 | -1.32014 | 0.999937 | 542.842 |
| 55 | 1.97735 | 0.101183 | -1.87617 | 1 | 385769 |
| 65 | 1.90935 | 0.182489 | -1.72686 | 0.999999 | 26887.2 |
| 71 | 1.56269 | 0.156571 | -1.40612 | 0.999999 | 38786.3 |
| 73 | 3.32852 | 0.2106 | -3.11792 | 0.999982 | 1868.78 |
| 78 | 1.96873 | 0.178951 | -1.78978 | 0.999967 | 1022.93 |
| 88 | 1.21379 | 0.0883002 | -1.12549 | 1 | 93899 |
| 90 | 0.958364 | 0.0301168 | -0.928247 | 1 | 2.21963e+08 |
| 91 | 1.3437 | 0.0385656 | -1.30514 | 1 | 678582 |
| 95 | 0.647456 | 0.0722137 | -0.575242 | 1 | 573425 |
| 102 | 1.31053 | 0.046611 | -1.26391 | 0.999997 | 11805.6 |
| 103 | 1.00872 | 0.096719 | -0.911996 | 1 | 74180.2 |
| 108 | 1.42286 | 0.107174 | -1.31569 | 1 | 84048.5 |
| 109 | 1.43468 | 0.0459179 | -1.38876 | 1 | 460899 |
| 112 | 1.12936 | 0.037926 | -1.09143 | 1 | 3.15918e+06 |
| 114 | 1.66338 | 0.163347 | -1.50003 | 1 | 2.98526e+06 |
| 116 | 1.60565 | 0.166577 | -1.43907 | 0.999999 | 25580.5 |
| 122 | 0.749483 | 0.037739 | -0.711744 | 1 | 1.27244e+06 |
| 126 | 1.43258 | 0.173264 | -1.25931 | 0.999999 | 42708.1 |
| 127 | 0.623614 | 0.0356882 | -0.587926 | 1 | 1.36727e+06 |
| 131 | 1.46545 | 0.120369 | -1.34508 | 0.999993 | 4778.77 |
| 133 | 0.552883 | 0.0326519 | -0.520231 | 1 | 2.31539e+07 |
| 135 | 0.855724 | 0.0384149 | -0.817309 | 1 | 501401 |
| 136 | 1.38835 | 0.047299 | -1.34105 | 1 | 860950 |
| 137 | 1.1825 | 0.0342366 | -1.14826 | 1 | 1.41592e+07 |
| 143 | 0.460696 | 0.0342364 | -0.426459 | 1 | 3.53464e+06 |
| 145 | 1.51392 | 0.185923 | -1.328 | 0.999983 | 1960.09 |
| 147 | 2.01065 | 0.16888 | -1.84177 | 1 | 239372 |
| 158 | 0.923731 | 0.0318216 | -0.891909 | 1 | 3.02548e+07 |
| 165 | 0.592662 | 0.0335936 | -0.559068 | 1 | 3.99523e+06 |
| 168 | 1.48021 | 0.107998 | -1.37221 | 0.999995 | 7405.1 |
| 174 | 1.39301 | 0.169614 | -1.2234 | 0.999995 | 6301.73 |
| 179 | 1.20731 | 0.0473107 | -1.16 | 0.999999 | 63582.6 |
| 181 | 1.1411 | 0.0960061 | -1.0451 | 0.999998 | 21399.4 |
| 182 | 1.29879 | 0.0361382 | -1.26266 | 1 | 1.25951e+06 |
| 183 | 0.506078 | 0.0335961 | -0.472482 | 1 | 3.77478e+06 |
| 188 | 0.598826 | 0.0369752 | -0.561851 | 1 | 1.51547e+06 |
| 189 | 1.06337 | 0.0347625 | -1.02861 | 1 | 6.29312e+06 |
| 190 | 0.776952 | 0.0308594 | -0.746092 | 1 | 4.06185e+07 |
| 192 | 2.98817 | 0.0320425 | -2.95613 | 1 | 7.58327e+09 |
| 193 | 0.890808 | 0.0336146 | -0.857194 | 1 | 8.36926e+06 |
| 194 | 1.38549 | 0.0715341 | -1.31395 | 1 | 1.19642e+07 |
| 195 | 0.659657 | 0.0342335 | -0.625423 | 1 | 3.98109e+06 |
| 197 | 1.10932 | 0.0389959 | -1.07032 | 1 | 1.76986e+06 |
| 201 | 1.36524 | 0.184659 | -1.18058 | 0.999992 | 4113.78 |
| 203 | 0.918587 | 0.0390461 | -0.879541 | 1 | 692265 |
| 205 | 1.45711 | 0.188057 | -1.26905 | 0.999942 | 590.066 |
| 206 | 2.6117 | 0.172247 | -2.43945 | 0.999999 | 59825.6 |
| 207 | 1.43865 | 0.174455 | -1.2642 | 0.999998 | 20682.9 |
| 208 | 3.35642 | 0.120115 | -3.2363 | 1 | 2.42847e+07 |
| 209 | 1.61776 | 0.0325889 | -1.58517 | 1 | 8.79992e+08 |
| 212 | 1.39364 | 0.0378856 | -1.35576 | 1 | 1.26908e+07 |
| 214 | 1.0363 | 0.0326112 | -1.00369 | 1 | 3.92755e+07 |
| 215 | 1.43692 | 0.0335981 | -1.40332 | 1 | 6.56816e+07 |
| 216 | 0.565126 | 0.0377039 | -0.527422 | 1 | 1.58889e+06 |
| 217 | 1.41647 | 0.100859 | -1.31561 | 1 | 228589 |
| 218 | 0.625497 | 0.0335777 | -0.591919 | 1 | 4.05624e+06 |
| 219 | 1.52243 | 0.0426077 | -1.47982 | 1 | 2.69245e+06 |
| 220 | 2.4444 | 0.0335995 | -2.4108 | 1 | 1.56794e+09 |
| 221 | 1.49142 | 0.0461 | -1.44532 | 1 | 2.10478e+06 |
| 222 | 1.13149 | 0.0370108 | -1.09448 | 1 | 7.62464e+06 |
| 224 | 0.485819 | 0.0386848 | -0.447134 | 1 | 451846 |
| 226 | 0.783821 | 0.0477089 | -0.736112 | 0.999998 | 19744.2 |
| 229 | 0.827774 | 0.0317333 | -0.796041 | 1 | 3.62791e+07 |
| 230 | 3.03735 | 0.0379897 | -2.99936 | 1 | 1.36234e+09 |
| 231 | 1.6233 | 0.0935008 | -1.5298 | 1 | 2.21789e+06 |
| 232 | 0.818118 | 0.0320773 | -0.786041 | 1 | 2.76368e+07 |
| 236 | 0.708467 | 0.0377227 | -0.670744 | 1 | 1.72623e+06 |
| 237 | 2.66725 | 0.169872 | -2.49738 | 0.999996 | 8311.52 |
| 239 | 1.2769 | 0.103196 | -1.17371 | 1 | 75111.5 |
| 242 | 2.08627 | 0.0464415 | -2.03983 | 1 | 2.3667e+07 |
| 243 | 0.575148 | 0.0326526 | -0.542495 | 1 | 2.52653e+07 |
| 244 | 1.47372 | 0.0764329 | -1.39729 | 1 | 4.97308e+06 |
| 245 | 2.54928 | 0.10179 | -2.44749 | 1 | 9.70973e+06 |
| 249 | 0.700261 | 0.0342359 | -0.666025 | 1 | 5.60519e+06 |
| 253 | 1.44746 | 0.182787 | -1.26468 | 0.999997 | 11552.3 |
| 254 | 0.406686 | 0.0336021 | -0.373084 | 1 | 3.32827e+06 |
| 255 | 0.748492 | 0.0356605 | -0.712831 | 1 | 1.62614e+06 |
| 256 | 1.06699 | 0.0384947 | -1.0285 | 1 | 953827 |
| 257 | 0.497465 | 0.0382929 | -0.459172 | 1 | 1.44123e+06 |
| 260 | 0.628038 | 0.0392609 | -0.588777 | 1 | 300981 |
| 261 | 3.49531 | 0.0392624 | -3.45605 | 1 | 2.49891e+09 |
| 262 | 1.51988 | 0.0968797 | -1.423 | 1 | 160228 |
| 265 | 1.18127 | 0.0780609 | -1.10321 | 1 | 1.43536e+06 |
| 266 | 0.466606 | 0.0878978 | -0.378708 | 0.999999 | 44593.2 |
| 269 | 1.33559 | 0.169545 | -1.16605 | 0.999997 | 13545.9 |
| 270 | 1.04158 | 0.034229 | -1.00735 | 1 | 5.2787e+06 |
| 276 | 0.60266 | 0.0392512 | -0.563409 | 1 | 264233 |
| 280 | 1.58263 | 0.170033 | -1.4126 | 1 | 156538 |
| 288 | 1.48697 | 0.168395 | -1.31858 | 0.999975 | 1349.11 |
| 290 | 1.76325 | 0.155453 | -1.6078 | 1 | 2.30744e+06 |
| 295 | 1.03145 | 0.0792001 | -0.952252 | 1 | 1.01124e+06 |
| 297 | 0.840596 | 0.0322587 | -0.808337 | 1 | 2.2363e+07 |
| 303 | 0.465585 | 0.0392222 | -0.426363 | 1 | 195831 |

**Table S8** Negative selection sites for the *N* gene based on FEL analysis.

| Codon | dS | dN | dN Leaves | dN/dS | Normalized dN-dS | p-value |
| --- | --- | --- | --- | --- | --- | --- |
| 8 | 1.25798 | 0 | 0 | 0.000 | -0.570214 | 0.0128927 |
| 18 | 2.63578 | 0.220896 | 0 | 0.084 | -1.09461 | 0.0323562 |
| 22 | 2.16153 | 0 | 0.203429 | 0.000 | -0.979769 | 0.00524567 |
| 35 | 3.32697 | 0 | 0.208722 | 0.000 | -1.50803 | 0.000483904 |
| 36 | 1.62527 | 0 | 0 | 0.000 | -0.736697 | 0.0099393 |
| 41 | 3.84064 | 0 | 0.193323 | 0.000 | -1.74087 | 0.000139474 |
| 42 | 0.680916 | 0 | 0.328099 | 0.000 | -0.308643 | 0.0489894 |
| 51 | 9.13572 | 0.220501 | 0.173828 | 0.024 | -4.04105 | 0.0010648 |
| 56 | 4.65921 | 0 | 0.180232 | 0.000 | -2.11191 | 0.000378485 |
| 60 | 8.98821 | 0.285342 | 0.20075 | 0.032 | -3.9448 | 0.00785631 |
| 64 | 1.41626 | 0 | 0 | 0.000 | -0.641957 | 0.01085 |
| 65 | 4.76493 | 0 | 0.418306 | 0.000 | -2.15983 | 0.000486142 |
| 66 | 1.12508 | 0 | 0 | 0.000 | -0.509971 | 0.0296394 |
| 67 | 1.42449 | 0 | 0 | 0.000 | -0.645687 | 0.0211107 |
| 68 | 4.64225 | 0 | 0 | 0.000 | -2.10422 | 0.000974498 |
| 70 | 1.05937 | 0 | 0 | 0.000 | -0.480188 | 0.0214849 |
| 73 | 2.0851 | 0 | 0.392126 | 0.000 | -0.945124 | 0.0100262 |
| 74 | 4.28511 | 0 | 0.708723 | 0.000 | -1.94234 | 0.000453601 |
| 75 | 9.99856 | 0 | 0.443808 | 0.000 | -4.5321 | 5.04859e-06 |
| 77 | 1.588 | 0 | 0.469453 | 0.000 | -0.719804 | 0.00696092 |
| 79 | 1.7509 | 0 | 0 | 0.000 | -0.793642 | 0.00574951 |
| 82 | 3.55313 | 0 | 0.535766 | 0.000 | -1.61055 | 0.00236745 |
| 83 | 1.40762 | 0 | 0 | 0.000 | -0.638041 | 0.0233106 |
| 84 | 2.93647 | 0 | 0.241681 | 0.000 | -1.33103 | 0.00304411 |
| 91 | 4.7425 | 0 | 0.400623 | 0.000 | -2.14966 | 7.07083e-05 |
| 93 | 0.999721 | 0 | 0.358609 | 0.000 | -0.453149 | 0.0137005 |
| 94 | 1.61886 | 0 | 0 | 0.000 | -0.73379 | 0.00798915 |
| 97 | 2.85692 | 0 | 0 | 0.000 | -1.29497 | 0.00115757 |
| 98 | 1.86105 | 0 | 0 | 0.000 | -0.84357 | 0.00634897 |
| 100 | 1.15516 | 0 | 0 | 0.000 | -0.523606 | 0.0296528 |
| 102 | 12.4162 | 0 | 0.165744 | 0.000 | -5.62797 | 1.09028e-05 |
| 105 | 2.0404 | 0 | 0.514289 | 0.000 | -0.924865 | 0.00453581 |
| 107 | 4.71722 | 0 | 0.168076 | 0.000 | -2.1382 | 0.000113775 |
| 108 | 4.11283 | 0.224285 | 0.183371 | 0.055 | -1.76258 | 0.0119839 |
| 112 | 5.37985 | 0 | 0.423905 | 0.000 | -2.43856 | 0.000502034 |
| 113 | 3.08201 | 0 | 0 | 0.000 | -1.397 | 0.00114538 |
| 114 | 1.18799 | 0 | 0 | 0.000 | -0.538487 | 0.0177272 |
| 121 | 4.71436 | 0 | 0 | 0.000 | -2.13691 | 0.000695036 |
| 125 | 6.57502 | 0.483835 | 0 | 0.074 | -2.76099 | 0.00404033 |
| 126 | 0.852631 | 0 | 0 | 0.000 | -0.386477 | 0.0279367 |
| 127 | 2.37368 | 0 | 0.448513 | 0.000 | -1.07593 | 0.00173282 |
| 129 | 6.03333 | 0 | 0 | 0.000 | -2.73476 | 2.42974e-05 |
| 130 | 1.49158 | 0 | 0 | 0.000 | -0.676096 | 0.0135768 |
| 135 | 2.90974 | 0 | 0.470584 | 0.000 | -1.31891 | 0.000876845 |
| 141 | 2.49999 | 0 | 0.214712 | 0.000 | -1.13318 | 0.00137671 |
| 143 | 2.84858 | 0 | 0 | 0.000 | -1.29119 | 0.000886539 |
| 147 | 3.90108 | 0.25599 | 0.207629 | 0.066 | -1.65223 | 0.00899645 |
| 148 | 0.848952 | 0 | 0 | 0.000 | -0.384809 | 0.0281223 |
| 151 | 26.1433 | 0 | 0.195942 | 0.000 | -11.8501 | 6.48571e-06 |
| 152 | 4.31878 | 0.265747 | 0.164169 | 0.062 | -1.83714 | 0.0254512 |
| 153 | 1.25313 | 0 | 0.212365 | 0.000 | -0.568014 | 0.0489584 |
| 171 | 1.72788 | 0 | 0 | 0.000 | -0.783207 | 0.0229724 |
| 173 | 2.65872 | 0 | 0 | 0.000 | -1.20514 | 0.0138225 |
| 178 | 4.79277 | 0.778008 | 0 | 0.162 | -1.81979 | 0.0316347 |
| 187 | 2.16561 | 0 | 0.393726 | 0.000 | -0.981619 | 0.0034476 |
| 190 | 0.918484 | 0 | 0 | 0.000 | -0.416327 | 0.0277505 |
| 220 | 2.17412 | 0 | 0 | 0.000 | -0.985477 | 0.00185484 |
| 247 | 1.69359 | 0 | 0.232346 | 0.000 | -0.767663 | 0.0171781 |
| 249 | 1.10154 | 0 | 0 | 0.000 | -0.4993 | 0.0201594 |
| 261 | 6.05859 | 0.233552 | 0.423836 | 0.039 | -2.64035 | 0.00744259 |
| 264 | 1.01009 | 0 | 0.557854 | 0.000 | -0.457849 | 0.0248983 |
| 265 | 0.768954 | 0 | 0.256856 | 0.000 | -0.348548 | 0.0385083 |
| 266 | 1.15371 | 0 | 0.513603 | 0.000 | -0.522949 | 0.0183291 |
| 267 | 4.147 | 0 | 0.178508 | 0.000 | -1.87973 | 0.00180644 |
| 268 | 8.9885 | 0.23441 | 0.1981 | 0.026 | -3.96802 | 0.000567104 |
| 272 | 1.36513 | 0 | 0.211633 | 0.000 | -0.618782 | 0.0293575 |
| 275 | 0.799205 | 0 | 0.514905 | 0.000 | -0.36226 | 0.0318389 |
| 278 | 1.12147 | 0 | 0.182706 | 0.000 | -0.508336 | 0.0291937 |
| 283 | 6.50693 | 0.214716 | 0 | 0.033 | -2.85211 | 0.00122616 |
| 284 | 0.948005 | 0 | 0 | 0.000 | -0.429708 | 0.03281 |
| 286 | 1.00456 | 0 | 0.462723 | 0.000 | -0.455344 | 0.0244911 |
| 304 | 1.25808 | 0 | 0.18948 | 0.000 | -0.570257 | 0.0149208 |
| 307 | 0.782273 | 0 | 0 | 0.000 | -0.354585 | 0.0416164 |
| 308 | 2.19524 | 0 | 0.18046 | 0.000 | -0.995051 | 0.00932797 |
| 309 | 1.70171 | 0 | 0.494972 | 0.000 | -0.771343 | 0.00830219 |
| 315 | 2.81629 | 0.197428 | 0 | 0.070 | -1.18707 | 0.0489117 |
| 318 | 1.43007 | 0 | 0 | 0.000 | -0.648218 | 0.01952 |
| 323 | 0.972353 | 0 | 0 | 0.000 | -0.440744 | 0.0438435 |
| 333 | 1.92806 | 0 | 0 | 0.000 | -0.873943 | 0.0129215 |
| 340 | 1.72119 | 0 | 0.240589 | 0.000 | -0.780173 | 0.00703712 |
| 341 | 1.75405 | 0 | 0 | 0.000 | -0.795066 | 0.0259338 |
| 344 | 1.35653 | 0 | 0.526951 | 0.000 | -0.61488 | 0.0412109 |
| 354 | 3.07424 | 0 | 0 | 0.000 | -1.39348 | 0.000526 |
| 355 | 3.19359 | 0 | 0.224905 | 0.000 | -1.44758 | 0.00130228 |
| 362 | 1.42449 | 0 | 0 | 0.000 | -0.645687 | 0.0178798 |
| 364 | 5.02801 | 0 | 0.627769 | 0.000 | -2.27907 | 0.000158158 |
| 365 | 0.878176 | 0 | 0 | 0.000 | -0.398056 | 0.0392501 |
| 368 | 4.23084 | 0.242614 | 0.56678 | 0.057 | -1.80777 | 0.00607366 |
| 371 | 0.890306 | 0 | 0 | 0.000 | -0.403554 | 0.0432962 |
| 373 | 3.33615 | 0.484763 | 0 | 0.145 | -1.29247 | 0.0335629 |
| 376 | 3.37933 | 0 | 0.706847 | 0.000 | -1.53177 | 0.000998157 |
| 379 | 2.34645 | 0 | 0.186866 | 0.000 | -1.06359 | 0.00419049 |
| 380 | 11.0356 | 0 | 0 | 0.000 | -5.00219 | 9.03322e-06 |
| 384 | 2.05534 | 0 | 0.178097 | 0.000 | -0.931637 | 0.00855886 |
| 385 | 3.89553 | 0 | 0.406225 | 0.000 | -1.76575 | 0.000239436 |
| 388 | 0.826059 | 0 | 0.15224 | 0.000 | -0.374433 | 0.026673 |
| 394 | 3.72112 | 0 | 0 | 0.000 | -1.68669 | 0.000372977 |
| 395 | 2.81199 | 0 | 0.198582 | 0.000 | -1.27461 | 0.00181629 |
| 397 | 7.29493 | 0.252383 | 0.27357 | 0.035 | -3.19221 | 0.00115142 |
| 398 | 4.1795 | 0 | 0.590821 | 0.000 | -1.89447 | 0.000115992 |
| 399 | 5.49034 | 0 | 0.560642 | 0.000 | -2.48864 | 0.000265951 |
| 403 | 1.27412 | 0 | 0.202681 | 0.000 | -0.577529 | 0.0206804 |
| 406 | 3.52121 | 0 | 0.19613 | 0.000 | -1.59608 | 0.000727072 |
| 408 | 6.25206 | 0 | 0.50807 | 0.000 | -2.83391 | 0.000102269 |
| 412 | 1.60544 | 0 | 0 | 0.000 | -0.727708 | 0.0215701 |
| 414 | 4.11539 | 0.238769 | 0.243177 | 0.058 | -1.75718 | 0.0214345 |
| 430 | 1.68127 | 0 | 0.546908 | 0.000 | -0.762081 | 0.0127372 |
| 440 | 2.71858 | 0 | 0.444305 | 0.000 | -1.23227 | 0.00234825 |
| 441 | 0.843639 | 0 | 0.475813 | 0.000 | -0.382401 | 0.032264 |
| 443 | 1.22335 | 0 | 0.438434 | 0.000 | -0.554513 | 0.0140694 |
| 455 | 0.957945 | 0 | 0.181649 | 0.000 | -0.434213 | 0.0379371 |

**Table S9** Negative selection sites for the *N* gene based on REL analysis.

| Codon | E[dS] | E[dN] | Normalized E[dN-dS] | Posterior Probability | Bayes Factor |
| --- | --- | --- | --- | --- | --- |
| 8 | 0.693981 | 0.0806222 | -0.613359 | 0.999973 | 1332.66 |
| 18 | 1.36855 | 0.102617 | -1.26593 | 0.999974 | 1404.63 |
| 22 | 1.31675 | 0.106048 | -1.2107 | 0.999911 | 408.254 |
| 35 | 1.95945 | 0.105323 | -1.85413 | 0.999893 | 337.336 |
| 36 | 0.966923 | 0.0733286 | -0.893594 | 0.999988 | 3146.95 |
| 41 | 1.94664 | 0.0918322 | -1.85481 | 0.999999 | 24463.5 |
| 42 | 0.552306 | 0.119499 | -0.432807 | 0.999837 | 222.615 |
| 51 | 2.03108 | 0.156103 | -1.87497 | 0.999989 | 3187.48 |
| 56 | 1.85683 | 0.0935694 | -1.76327 | 0.999996 | 9423.71 |
| 60 | 2.03875 | 0.189788 | -1.84896 | 0.999923 | 470.819 |
| 64 | 0.889186 | 0.064874 | -0.824312 | 0.999999 | 27278 |
| 65 | 1.81802 | 0.161443 | -1.65658 | 0.999905 | 381.536 |
| 66 | 0.83545 | 0.066668 | -0.768782 | 0.999998 | 15086.4 |
| 67 | 0.902837 | 0.069314 | -0.833523 | 0.999997 | 11513.8 |
| 68 | 1.71503 | 0.0689375 | -1.64609 | 0.999999 | 40687.4 |
| 70 | 0.645307 | 0.0712435 | -0.574063 | 0.999992 | 4348.47 |
| 73 | 1.1253 | 0.147717 | -0.977585 | 0.99985 | 241.589 |
| 74 | 1.96516 | 0.22605 | -1.73911 | 0.999276 | 50.0136 |
| 75 | 2.0646 | 0.171856 | -1.89275 | 0.999994 | 6265.61 |
| 77 | 0.92062 | 0.167808 | -0.752813 | 0.999697 | 119.437 |
| 79 | 1.01739 | 0.0701305 | -0.947256 | 0.999993 | 5181.6 |
| 82 | 1.72828 | 0.179368 | -1.54891 | 0.999549 | 80.2341 |
| 83 | 0.891023 | 0.0714782 | -0.819544 | 0.999994 | 6239.38 |
| 84 | 1.49747 | 0.113284 | -1.38419 | 0.999951 | 738.473 |
| 91 | 2.03187 | 0.158519 | -1.87336 | 0.999995 | 6955.69 |
| 93 | 0.667136 | 0.134165 | -0.532971 | 0.999975 | 1440.14 |
| 94 | 0.939845 | 0.0611842 | -0.878661 | 1 | 347740 |
| 97 | 1.73196 | 0.0720219 | -1.65994 | 0.999996 | 8172.75 |
| 98 | 1.11397 | 0.0723114 | -1.04165 | 0.999993 | 5348.04 |
| 100 | 0.763869 | 0.0734631 | -0.690405 | 0.999989 | 3431.06 |
| 102 | 2.06106 | 0.0867203 | -1.97434 | 1 | 176170 |
| 105 | 1.23611 | 0.183731 | -1.05238 | 0.999637 | 99.8171 |
| 107 | 1.88767 | 0.0810613 | -1.80661 | 1 | 83915.3 |
| 108 | 1.75437 | 0.160251 | -1.59412 | 0.999932 | 530.578 |
| 112 | 1.77708 | 0.160298 | -1.61679 | 0.999942 | 628.248 |
| 113 | 1.73025 | 0.072434 | -1.65782 | 0.999997 | 13264.2 |
| 114 | 0.719246 | 0.0679259 | -0.65132 | 0.999997 | 12128.8 |
| 121 | 1.78648 | 0.0716997 | -1.71478 | 0.999998 | 23902.4 |
| 125 | 2.01988 | 0.182648 | -1.83723 | 0.999952 | 751.041 |
| 126 | 0.607315 | 0.0668968 | -0.540419 | 0.999998 | 15030.7 |
| 127 | 1.44717 | 0.163103 | -1.28407 | 0.999776 | 161.574 |
| 129 | 2.0649 | 0.0703074 | -1.99459 | 0.999999 | 29587.1 |
| 130 | 0.837993 | 0.0716636 | -0.76633 | 0.999993 | 5243.6 |
| 135 | 1.6216 | 0.165591 | -1.45601 | 0.999925 | 484.933 |
| 141 | 1.73416 | 0.107508 | -1.62666 | 0.999979 | 1752.82 |
| 143 | 1.48427 | 0.0611675 | -1.4231 | 1 | 594183 |
| 147 | 1.93648 | 0.176224 | -1.76026 | 0.999949 | 714.251 |
| 148 | 0.605561 | 0.0668969 | -0.538664 | 0.999998 | 15015 |
| 151 | 2.01422 | 0.0851354 | -1.92908 | 1 | 158983 |
| 152 | 1.57025 | 0.167062 | -1.40319 | 0.999857 | 253.806 |
| 153 | 0.7114 | 0.113627 | -0.597773 | 0.999852 | 244.029 |
| 171 | 0.998498 | 0.0878611 | -0.910637 | 0.999689 | 116.458 |
| 173 | 1.14545 | 0.064819 | -1.08063 | 0.999999 | 38227 |
| 177 | 1.50512 | 0.125474 | -1.37965 | 0.999627 | 97.1076 |
| 178 | 1.94897 | 0.232539 | -1.71643 | 0.999574 | 84.9589 |
| 187 | 1.2675 | 0.157826 | -1.10968 | 0.999902 | 368.545 |
| 190 | 0.639077 | 0.0667211 | -0.572355 | 0.999998 | 19465.7 |
| 220 | 1.33203 | 0.0692387 | -1.26279 | 0.999995 | 7244.65 |
| 247 | 0.989922 | 0.110183 | -0.879739 | 0.999933 | 543.122 |
| 249 | 0.68985 | 0.0677528 | -0.622097 | 0.999996 | 8976.54 |
| 261 | 1.83888 | 0.223438 | -1.61544 | 0.999568 | 83.8748 |
| 264 | 0.635063 | 0.186602 | -0.448461 | 0.99931 | 52.4885 |
| 265 | 0.570267 | 0.111624 | -0.458643 | 0.999946 | 668.484 |
| 266 | 0.74116 | 0.179707 | -0.561454 | 0.999635 | 99.2177 |
| 267 | 1.49303 | 0.0910463 | -1.40198 | 0.999991 | 3832.7 |
| 268 | 2.03934 | 0.165007 | -1.87434 | 0.999994 | 6459.45 |
| 272 | 0.788302 | 0.110291 | -0.678011 | 0.999798 | 179.747 |
| 275 | 0.580735 | 0.175636 | -0.405099 | 0.999605 | 91.5952 |
| 278 | 0.804975 | 0.0937516 | -0.711224 | 0.999985 | 2448.47 |
| 283 | 1.95569 | 0.0965236 | -1.85916 | 0.999996 | 8598.94 |
| 284 | 0.79616 | 0.0648696 | -0.73129 | 0.999999 | 26505.4 |
| 286 | 0.800508 | 0.164614 | -0.635893 | 0.999819 | 199.689 |
| 304 | 0.732764 | 0.0973954 | -0.635369 | 0.999967 | 1108.55 |
| 307 | 0.600186 | 0.0714154 | -0.52877 | 0.999993 | 5009.58 |
| 308 | 1.15814 | 0.0921684 | -1.06597 | 0.999988 | 2934.77 |
| 309 | 1.00941 | 0.187027 | -0.822383 | 0.999354 | 56.0122 |
| 315 | 1.22669 | 0.0904289 | -1.13626 | 0.999984 | 2312.62 |
| 318 | 0.833839 | 0.070109 | -0.76373 | 0.999988 | 3069.92 |
| 323 | 0.730869 | 0.0648227 | -0.666046 | 0.999999 | 25962.8 |
| 333 | 1.06944 | 0.0714902 | -0.997948 | 0.999995 | 6930.18 |
| 340 | 0.989341 | 0.106736 | -0.882605 | 0.999956 | 817.357 |
| 341 | 0.991026 | 0.0712968 | -0.91973 | 0.999995 | 7609.75 |
| 344 | 0.922852 | 0.191992 | -0.73086 | 0.999633 | 98.6988 |
| 354 | 1.63709 | 0.061147 | -1.57595 | 1 | 809906 |
| 355 | 1.69903 | 0.111016 | -1.58801 | 0.999969 | 1176.22 |
| 362 | 0.903129 | 0.0667685 | -0.83636 | 0.999998 | 16644.2 |
| 364 | 2.02407 | 0.194396 | -1.82968 | 0.999932 | 535.872 |
| 365 | 0.695861 | 0.0666858 | -0.629175 | 0.999997 | 13311.1 |
| 368 | 1.98767 | 0.235105 | -1.75256 | 0.999681 | 113.706 |
| 371 | 0.650272 | 0.0677873 | -0.582485 | 0.999996 | 8838.78 |
| 373 | 1.79155 | 0.185192 | -1.60636 | 0.999745 | 142.02 |
| 376 | 1.73722 | 0.226316 | -1.5109 | 0.999572 | 84.5299 |
| 380 | 2.03511 | 0.066458 | -1.96865 | 1 | 433027 |
| 384 | 1.13306 | 0.0927635 | -1.04029 | 0.999983 | 2162.72 |
| 385 | 1.9945 | 0.163572 | -1.83093 | 0.999988 | 2999.09 |
| 388 | 0.624992 | 0.0777279 | -0.547264 | 0.999998 | 20388.4 |
| 394 | 1.89947 | 0.070222 | -1.82924 | 0.999999 | 40152.5 |
| 395 | 1.55845 | 0.101704 | -1.45675 | 0.999985 | 2390.69 |
| 397 | 2.07585 | 0.196171 | -1.87968 | 0.999992 | 4773.78 |
| 398 | 2.00414 | 0.184801 | -1.81934 | 0.999961 | 926.798 |
| 399 | 1.97437 | 0.189986 | -1.78438 | 0.999867 | 273.073 |
| 403 | 0.828923 | 0.0974662 | -0.731457 | 0.999972 | 1281.73 |
| 406 | 1.93866 | 0.101661 | -1.837 | 0.999939 | 595.28 |
| 408 | 2.05294 | 0.179088 | -1.87385 | 0.999888 | 324.854 |
| 412 | 0.929999 | 0.0704988 | -0.8595 | 0.999989 | 3386.75 |
| 414 | 1.92327 | 0.184948 | -1.73832 | 0.999362 | 56.7432 |
| 430 | 0.97608 | 0.180879 | -0.795201 | 0.999508 | 73.642 |
| 440 | 1.49979 | 0.171943 | -1.32785 | 0.999878 | 296.06 |
| 441 | 0.603592 | 0.173601 | -0.429991 | 0.999592 | 88.7797 |
| 443 | 0.701757 | 0.167124 | -0.534633 | 0.999616 | 94.2909 |
| 455 | 0.806547 | 0.0939268 | -0.71262 | 0.999978 | 1643.98 |

**Table S10** Negative selection sites for the *S* gene based on FEL analysis.

| Codon | dS | dN | dN Leaves | dN/dS | Normalized dN-dS | p-value |
| --- | --- | --- | --- | --- | --- | --- |
| 8 | 4856.24 | 0.0762134 | 0.303953 | 0.000 | -1435.88 | 0.0010778 |
| 11 | 3.22661 | 0 | 0.385838 | 0.000 | -0.954051 | 6.83761e-06 |
| 16 | 18.8592 | 0 | 3.92867 | 0.000 | -5.57634 | 1.33295e-05 |
| 40 | 10.4675 | 0.530311 | 0 | 0.051 | -2.93825 | 0.00909465 |
| 41 | 2.40353 | 0 | 0.636137 | 0.000 | -0.710682 | 0.000143583 |
| 56 | 7.49524 | 1.36192 | 0 | 0.182 | -1.81352 | 0.0453403 |
| 71 | 1.59431 | 0.140808 | 0 | 0.088 | -0.429776 | 0.019433 |
| 72 | 0.793255 | 0 | 0 | 0.000 | -0.234552 | 0.0107463 |
| 73 | 1.3246 | 0.126987 | 0.151941 | 0.096 | -0.354114 | 0.0296447 |
| 74 | 1.28014 | 0 | 0 | 0.000 | -0.378516 | 0.00328159 |
| 75 | 2.95604 | 0.361262 | 0.0643699 | 0.122 | -0.767229 | 0.022182 |
| 76 | 3.30218 | 0 | 0 | 0.000 | -0.976398 | 3.46933e-06 |
| 85 | 10.5808 | 0 | 0 | 0.000 | -3.12856 | 0.00289565 |
| 89 | 15.8763 | 0.271655 | 0.318539 | 0.017 | -4.61403 | 0.000479537 |
| 114 | 18.0685 | 0 | 0 | 0.000 | -5.34254 | 0.00085195 |
| 115 | 7.13072 | 0.358807 | 0.307366 | 0.050 | -2.00234 | 0.0306542 |
| 118 | 3.96345 | 0.249896 | 0.00335246 | 0.063 | -1.09803 | 0.00229155 |
| 119 | 10.6169 | 0.465114 | 0.127558 | 0.044 | -3.0017 | 0.0121537 |
| 120 | 2.23129 | 0.129374 | 0 | 0.058 | -0.6215 | 0.0159105 |
| 122 | 4.36165 | 0.348654 | 0 | 0.080 | -1.18657 | 0.0065724 |
| 125 | 3.24266 | 0.273742 | 0.310097 | 0.084 | -0.877856 | 0.0148257 |
| 137 | 18.5207 | 0 | 0.58415 | 0.000 | -5.47625 | 9.59559e-08 |
| 142 | 1.27008 | 0.133406 | 0.176511 | 0.105 | -0.336095 | 0.0298748 |
| 144 | 20.5698 | 0.52161 | 0.47411 | 0.025 | -5.92791 | 0.00089148 |
| 146 | 4.61821 | 0.200798 | 0.759299 | 0.043 | -1.30615 | 0.000749064 |
| 148 | 3.34701 | 0.285702 | 0.365025 | 0.085 | -0.905174 | 0.00215638 |
| 150 | 4.16752 | 0.0952736 | 0.434354 | 0.023 | -1.20409 | 0.000308969 |
| 178 | 3.65957 | 0 | 0.349978 | 0.000 | -1.08207 | 4.25216e-05 |
| 233 | 10.7424 | 0 | 0.167301 | 0.000 | -3.17634 | 0.000216643 |
| 235 | 5.73275 | 0.416916 | 0.457227 | 0.073 | -1.5718 | 0.0022581 |
| 241 | 1.78606 | 0.124958 | 0.362298 | 0.070 | -0.49116 | 0.00764654 |
| 243 | 3.37793 | 0.297417 | 0.446318 | 0.088 | -0.910853 | 0.00138249 |
| 244 | 4.60558 | 0.129677 | 0.170129 | 0.028 | -1.32345 | 0.00247992 |
| 245 | 2.87149 | 0.176252 | 0.177453 | 0.061 | -0.796934 | 0.00654266 |
| 256 | 3.30214 | 0.102054 | 0.137204 | 0.031 | -0.94621 | 0.00140877 |
| 262 | 4.40232 | 0.295009 | 0.48106 | 0.067 | -1.21446 | 0.00302682 |
| 263 | 1.75917 | 0.104854 | 0.437055 | 0.060 | -0.489152 | 0.00278463 |
| 267 | 23.8958 | 2.48884 | 0.908646 | 0.104 | -6.32967 | 0.0181177 |
| 287 | 0.577829 | 0 | 0 | 0.000 | -0.170854 | 0.0284164 |
| 291 | 3.38113 | 0 | 0.139677 | 0.000 | -0.99974 | 3.5294e-06 |
| 292 | 0.670347 | 0 | 0 | 0.000 | -0.19821 | 0.0259479 |
| 293 | 0.556422 | 0 | 0 | 0.000 | -0.164524 | 0.0238173 |
| 296 | 2.71927 | 0 | 0.287171 | 0.000 | -0.80404 | 0.000151233 |
| 298 | 0.739087 | 0 | 0.636788 | 0.000 | -0.218535 | 0.00916219 |
| 320 | 1.05292 | 0 | 0 | 0.000 | -0.311329 | 0.00515299 |
| 321 | 2.43217 | 0.220044 | 0.523925 | 0.090 | -0.654086 | 0.0111879 |
| 323 | 5.31907 | 0.124127 | 0.527752 | 0.023 | -1.53605 | 8.63527e-05 |
| 326 | 2.27804 | 0 | 0 | 0.000 | -0.673576 | 0.000155427 |
| 327 | 1.20999 | 0.118097 | 0.325073 | 0.098 | -0.322854 | 0.0152497 |
| 334 | 0.657509 | 0 | 0 | 0.000 | -0.194414 | 0.0107962 |
| 337 | 0.929938 | 0 | 0 | 0.000 | -0.274966 | 0.0022129 |
| 340 | 8.70854 | 0 | 0 | 0.000 | -2.57496 | 0.00030887 |
| 341 | 1.83861 | 0.235396 | 0 | 0.128 | -0.474041 | 0.0496482 |
| 342 | 0.99229 | 0 | 0 | 0.000 | -0.293403 | 0.013251 |
| 343 | 1.84475 | 0.103473 | 0 | 0.056 | -0.514865 | 0.00386468 |
| 344 | 1.08814 | 0 | 0 | 0.000 | -0.321745 | 0.00367203 |
| 346 | 1.64998 | 0 | 0 | 0.000 | -0.48787 | 0.0101637 |
| 348 | 0.842665 | 0 | 0 | 0.000 | -0.249161 | 0.00883236 |
| 349 | 1.08202 | 0.115689 | 0 | 0.107 | -0.285727 | 0.0246049 |
| 352 | 1.40569 | 0 | 0 | 0.000 | -0.415639 | 0.00026213 |
| 353 | 7.4182 | 0 | 0.138533 | 0.000 | -2.19343 | 1.52332e-07 |
| 357 | 1.0253 | 0 | 0 | 0.000 | -0.303163 | 0.00259205 |
| 363 | 5.87178 | 0 | 0 | 0.000 | -1.73618 | 1.59615e-06 |
| 364 | 1.03603 | 0 | 0 | 0.000 | -0.306336 | 0.00076551 |
| 365 | 1.96934 | 0.148996 | 0 | 0.076 | -0.538243 | 0.00607881 |
| 366 | 1.84046 | 0.132729 | 0.153377 | 0.072 | -0.504945 | 0.00748119 |
| 367 | 1.25116 | 0.117938 | 0 | 0.094 | -0.335073 | 0.018172 |
| 368 | 3.08513 | 0.2271 | 0.154185 | 0.074 | -0.845069 | 0.0238881 |
| 369 | 9.66013 | 0 | 0 | 0.000 | -2.85633 | 2.1341e-06 |
| 370 | 4.90253 | 0 | 0.177957 | 0.000 | -1.44959 | 4.95615e-06 |
| 373 | 1.54654 | 0.122113 | 0 | 0.079 | -0.421177 | 0.0086966 |
| 374 | 1.88266 | 0 | 0 | 0.000 | -0.556668 | 6.03596e-05 |
| 383 | 2.07915 | 0.191303 | 0.40254 | 0.092 | -0.558202 | 0.0405644 |
| 385 | 0.710756 | 0 | 0 | 0.000 | -0.210158 | 0.0164017 |
| 388 | 6.55059 | 0.374652 | 0.479934 | 0.057 | -1.82612 | 0.00195968 |
| 392 | 26.8614 | 0.419263 | 0.417621 | 0.016 | -7.81847 | 0.000288745 |
| 393 | 3.02841 | 0 | 0 | 0.000 | -0.895447 | 0.00133713 |
| 399 | 4.0274 | 0.134201 | 0 | 0.033 | -1.15115 | 0.00836281 |
| 401 | 1.48347 | 0.209368 | 0.355253 | 0.141 | -0.376729 | 0.0417674 |
| 406 | 2.19615 | 0 | 0 | 0.000 | -0.649363 | 7.36529e-05 |
| 407 | 1.20259 | 0 | 0 | 0.000 | -0.355584 | 0.000912196 |
| 408 | 95.4452 | 0 | 0.283557 | 0.000 | -28.2215 | 8.71355e-05 |
| 409 | 3.80992 | 0.11513 | 0.15283 | 0.030 | -1.09248 | 6.41666e-05 |
| 411 | 7.55108 | 0.235202 | 0.272095 | 0.031 | -2.16318 | 0.00640595 |
| 412 | 2.74294 | 0.108651 | 0.133671 | 0.040 | -0.778913 | 0.00109155 |
| 417 | 2.29841 | 0.322124 | 0.313107 | 0.140 | -0.584352 | 0.0482451 |
| 426 | 1.22801 | 0 | 0.45113 | 0.000 | -0.3631 | 0.00148148 |
| 427 | 54.585 | 0.220151 | 0.370826 | 0.004 | -16.0747 | 0.0086007 |
| 428 | 1.2155 | 0.229414 | 0.285543 | 0.189 | -0.291569 | 0.0493423 |
| 429 | 0.948434 | 0.100655 | 0 | 0.106 | -0.250673 | 0.0269579 |
| 436 | 2.24524 | 0.256013 | 0 | 0.114 | -0.588181 | 0.00983845 |
| 437 | 15.8526 | 0.56725 | 0.250609 | 0.036 | -4.51962 | 0.00451433 |
| 439 | 5.94454 | 0.261757 | 0 | 0.044 | -1.6803 | 0.00191406 |
| 440 | 2.63916 | 0 | 0 | 0.000 | -0.780354 | 0.000617185 |
| 459 | 1.57333 | 0 | 0.853232 | 0.000 | -0.465205 | 0.00325471 |
| 460 | 1.1933 | 0.0876466 | 0 | 0.073 | -0.326922 | 0.00437571 |
| 461 | 1.11459 | 0.103804 | 0 | 0.093 | -0.29887 | 0.0349039 |
| 462 | 0.988443 | 0 | 0.163415 | 0.000 | -0.292265 | 0.00210376 |
| 467 | 3.61993 | 0.317228 | 0 | 0.088 | -0.97655 | 0.00153637 |
| 468 | 4.07637 | 0.558937 | 0.176917 | 0.137 | -1.04004 | 0.0187623 |
| 470 | 0.706101 | 0 | 0 | 0.000 | -0.208782 | 0.0222736 |
| 477 | 8.99245 | 0.128471 | 0.337108 | 0.014 | -2.62092 | 0.000158654 |
| 480 | 2.33995 | 0.114032 | 0.267845 | 0.049 | -0.658166 | 0.00125611 |
| 483 | 4.17128 | 0.22253 | 0.291 | 0.053 | -1.16758 | 0.000345987 |
| 486 | 6.17464 | 0.104575 | 0.147315 | 0.017 | -1.79481 | 0.000186073 |
| 490 | 2.66378 | 0 | 0 | 0.000 | -0.787632 | 2.94128e-05 |
| 491 | 0.341826 | 0 | 0 | 0.000 | -0.101072 | 0.0379002 |
| 492 | 0.816617 | 0 | 0 | 0.000 | -0.241459 | 0.00587763 |
| 494 | 11.1179 | 0 | 0 | 0.000 | -3.28737 | 1.00437e-06 |
| 495 | 1.66913 | 0.143318 | 0.469299 | 0.086 | -0.451155 | 0.0141577 |
| 496 | 1.90367 | 0 | 0 | 0.000 | -0.562883 | 0.000626155 |
| 498 | 0.831224 | 0.1056 | 0 | 0.127 | -0.214554 | 0.0479847 |
| 501 | 3.11936 | 0.1064 | 0 | 0.034 | -0.890878 | 0.000440768 |
| 503 | 0.59263 | 0 | 0 | 0.000 | -0.17523 | 0.0178009 |
| 509 | 1.28612 | 0 | 0 | 0.000 | -0.380284 | 0.000871458 |
| 513 | 2.31462 | 0.103963 | 0 | 0.045 | -0.653653 | 0.00857435 |
| 515 | 5.75095 | 0.536324 | 0 | 0.093 | -1.54187 | 0.00163283 |
| 521 | 1.38771 | 0.241517 | 0 | 0.174 | -0.338909 | 0.0445561 |
| 523 | 1.9107 | 0.105115 | 0 | 0.055 | -0.533881 | 0.0085164 |
| 524 | 1.60437 | 0.237156 | 0 | 0.148 | -0.40426 | 0.0234141 |
| 526 | 1.26143 | 0.10837 | 0 | 0.086 | -0.340938 | 0.00988217 |
| 536 | 1.88777 | 0.126927 | 0.301993 | 0.067 | -0.520649 | 0.0105424 |
| 537 | 2.58117 | 0 | 0.132127 | 0.000 | -0.763206 | 0.000180349 |
| 539 | 1.21251 | 0 | 0 | 0.000 | -0.358518 | 0.000700251 |
| 540 | 0.970824 | 0 | 0.331401 | 0.000 | -0.287056 | 0.00930341 |
| 541 | 2.40609 | 0 | 0.137335 | 0.000 | -0.71144 | 7.64467e-05 |
| 542 | 1.96797 | 0.129245 | 0 | 0.066 | -0.54368 | 0.00655556 |
| 543 | 0.893544 | 0 | 0 | 0.000 | -0.264205 | 0.00212781 |
| 552 | 0.664694 | 0 | 0 | 0.000 | -0.196538 | 0.0152999 |
| 555 | 2.19471 | 0 | 0.133215 | 0.000 | -0.648938 | 8.8288e-05 |
| 557 | 1.49923 | 0 | 0 | 0.000 | -0.443297 | 0.00100978 |
| 558 | 3.60297 | 0.237521 | 0 | 0.066 | -0.995103 | 0.00221905 |
| 559 | 3.0165 | 0.363208 | 0 | 0.120 | -0.784532 | 0.0155728 |
| 560 | 2.64963 | 0.314304 | 0.136713 | 0.119 | -0.690514 | 0.0126063 |
| 563 | 1.86909 | 0 | 0 | 0.000 | -0.552658 | 0.00955126 |
| 568 | 1.23387 | 0.106493 | 0.138074 | 0.086 | -0.333347 | 0.0364481 |
| 570 | 2.56618 | 0.111986 | 0 | 0.044 | -0.725661 | 0.00398018 |
| 571 | 3.19053 | 0.10501 | 0 | 0.033 | -0.912335 | 0.000782027 |
| 572 | 2.58186 | 0 | 0 | 0.000 | -0.763412 | 0.00116396 |
| 574 | 2.29481 | 0 | 0 | 0.000 | -0.678534 | 0.000288846 |
| 575 | 2.02537 | 0.134272 | 0.323202 | 0.066 | -0.559166 | 0.0100885 |
| 579 | 2.43329 | 0 | 0 | 0.000 | -0.71948 | 6.11779e-05 |
| 582 | 2.74003 | 0 | 0 | 0.000 | -0.810178 | 1.58602e-05 |
| 583 | 1.73672 | 0 | 0 | 0.000 | -0.513517 | 0.0136758 |
| 584 | 0.668854 | 0 | 0 | 0.000 | -0.197768 | 0.0179691 |
| 586 | 2.60083 | 0.253836 | 0 | 0.098 | -0.693965 | 0.00396222 |
| 594 | 3.77679 | 0.359225 | 0.34012 | 0.095 | -1.01052 | 0.00796899 |
| 596 | 2.06712 | 0.112052 | 0.307651 | 0.054 | -0.57808 | 0.00559137 |
| 601 | 2.17267 | 0 | 0 | 0.000 | -0.64242 | 0.00013313 |
| 603 | 1.6392 | 0.130583 | 0 | 0.080 | -0.446073 | 0.00705155 |
| 604 | 0.818616 | 0 | 0 | 0.000 | -0.24205 | 0.00515919 |
| 606 | 5.80099 | 0.218318 | 0 | 0.038 | -1.6507 | 0.013143 |
| 609 | 6.78399 | 0.11653 | 0 | 0.017 | -1.97145 | 0.00426511 |
| 613 | 2.7519 | 0 | 0.138322 | 0.000 | -0.813688 | 0.00134503 |
| 615 | 4.20561 | 0.101212 | 0.138348 | 0.024 | -1.2136 | 5.07459e-05 |
| 620 | 4.30057 | 0.274348 | 0.301061 | 0.064 | -1.19048 | 0.00252875 |
| 626 | 2.84018 | 0.137669 | 0.552461 | 0.048 | -0.799086 | 0.000845086 |
| 631 | 10.4428 | 0.75494 | 0.223076 | 0.072 | -2.86454 | 0.00327319 |
| 647 | 29.7329 | 0.340177 | 0.339052 | 0.011 | -8.69092 | 0.00217454 |
| 663 | 4.19033 | 0 | 0 | 0.000 | -1.23901 | 0.000823966 |
| 668 | 2.47456 | 0.110744 | 0 | 0.045 | -0.698939 | 0.00197258 |
| 669 | 3.19377 | 0.105104 | 0 | 0.033 | -0.913265 | 0.000396764 |
| 670 | 1.45962 | 0.247707 | 0 | 0.170 | -0.358341 | 0.0392403 |
| 677 | 3.25367 | 0.151223 | 0 | 0.046 | -0.917339 | 0.0107092 |
| 696 | 4.17945 | 0.53031 | 0.358523 | 0.127 | -1.07899 | 0.0123014 |
| 705 | 2.85949 | 0 | 0 | 0.000 | -0.845502 | 9.66285e-05 |
| 706 | 0.406929 | 0 | 0 | 0.000 | -0.120322 | 0.0321214 |
| 707 | 0.464672 | 0 | 0 | 0.000 | -0.137395 | 0.0306141 |
| 708 | 1.50293 | 0.268978 | 0 | 0.179 | -0.364859 | 0.0452318 |
| 710 | 2.17502 | 0.26149 | 0 | 0.120 | -0.565796 | 0.03073 |
| 713 | 1.84502 | 0 | 0 | 0.000 | -0.545539 | 0.000402284 |
| 715 | 1.16926 | 0.121959 | 0 | 0.104 | -0.30967 | 0.0282286 |
| 716 | 1.441 | 0.129291 | 0.151423 | 0.090 | -0.387849 | 0.0134812 |
| 721 | 3.03047 | 0.350608 | 0.247411 | 0.116 | -0.792389 | 0.00565771 |
| 727 | 1.06701 | 0.186029 | 0 | 0.174 | -0.26049 | 0.0383278 |
| 732 | 1.22999 | 0 | 0 | 0.000 | -0.363686 | 0.00160624 |
| 736 | 2.13811 | 0.130873 | 0 | 0.061 | -0.593505 | 0.00371636 |
| 746 | 0.769137 | 0 | 0 | 0.000 | -0.22742 | 0.0297681 |
| 751 | 2.02263 | 0.317012 | 0 | 0.157 | -0.504321 | 0.042063 |
| 752 | 3.39023 | 0.284209 | 0.160121 | 0.084 | -0.918395 | 0.0464733 |
| 753 | 1.49823 | 0.12714 | 0 | 0.085 | -0.405406 | 0.0185223 |
| 755 | 1.90921 | 0.129206 | 0 | 0.068 | -0.526316 | 0.0339313 |
| 761 | 1.56712 | 0 | 0.31012 | 0.000 | -0.46337 | 0.00028103 |
| 765 | 1.19245 | 0.0846424 | 0.143442 | 0.071 | -0.32756 | 0.00564406 |
| 771 | 3.18771 | 0.440878 | 0.155837 | 0.138 | -0.812191 | 0.0295387 |
| 773 | 3.93044 | 0 | 0 | 0.000 | -1.16216 | 0.000121673 |
| 774 | 2.30165 | 0.298857 | 0.313064 | 0.130 | -0.592191 | 0.0440197 |
| 775 | 1.25064 | 0 | 0.501409 | 0.000 | -0.369793 | 0.00079309 |
| 777 | 1.40117 | 0.130912 | 0.390279 | 0.093 | -0.375592 | 0.0116489 |
| 782 | 4.07368 | 0 | 0 | 0.000 | -1.20452 | 7.54052e-05 |
| 783 | 0.687014 | 0 | 0 | 0.000 | -0.203138 | 0.00793037 |
| 785 | 2.32242 | 0 | 0 | 0.000 | -0.686699 | 0.000401155 |
| 787 | 1.19785 | 0.104902 | 0 | 0.088 | -0.323167 | 0.0183253 |
| 788 | 0.996557 | 0 | 0 | 0.000 | -0.294664 | 0.0199407 |
| 789 | 1.56788 | 0 | 0 | 0.000 | -0.463594 | 0.000546384 |
| 792 | 0.711954 | 0 | 0 | 0.000 | -0.210512 | 0.0078058 |
| 794 | 0.644025 | 0 | 0 | 0.000 | -0.190427 | 0.014341 |
| 796 | 0.600448 | 0 | 0 | 0.000 | -0.177542 | 0.0231777 |
| 797 | 2.33715 | 0.13593 | 0 | 0.058 | -0.650862 | 0.00449769 |
| 799 | 0.927907 | 0 | 0 | 0.000 | -0.274366 | 0.00162755 |
| 800 | 1.56592 | 0.105501 | 0 | 0.067 | -0.431822 | 0.00377236 |
| 802 | 7.5747 | 0.399046 | 0.307389 | 0.053 | -2.12171 | 0.000567498 |
| 806 | 0.49964 | 0 | 0 | 0.000 | -0.147735 | 0.0180142 |
| 808 | 2.18908 | 0 | 0 | 0.000 | -0.647273 | 0.000356621 |
| 809 | 0.368812 | 0 | 0 | 0.000 | -0.109051 | 0.0434637 |
| 810 | 0.924821 | 0 | 0 | 0.000 | -0.273453 | 0.00305607 |
| 811 | 0.691679 | 0 | 0 | 0.000 | -0.204517 | 0.0112277 |
| 814 | 0.434269 | 0 | 0 | 0.000 | -0.128406 | 0.0468581 |
| 815 | 4.3853 | 0.240248 | 0 | 0.055 | -1.22562 | 0.00355049 |
| 816 | 2.11197 | 0 | 0 | 0.000 | -0.624472 | 0.000134081 |
| 817 | 1.60236 | 0 | 0 | 0.000 | -0.473789 | 0.000884006 |
| 818 | 1.51027 | 0.12628 | 0 | 0.084 | -0.409221 | 0.0161105 |
| 820 | 1.05761 | 0 | 0 | 0.000 | -0.312715 | 0.00820148 |
| 825 | 0.802901 | 0 | 0 | 0.000 | -0.237404 | 0.0121495 |
| 830 | 2.04034 | 0.120464 | 0 | 0.059 | -0.567673 | 0.00217621 |
| 832 | 0.576788 | 0 | 0 | 0.000 | -0.170546 | 0.0100072 |
| 836 | 2.60197 | 0.249136 | 0 | 0.096 | -0.695691 | 0.0107113 |
| 837 | 0.872192 | 0.1109 | 0.137198 | 0.127 | -0.225101 | 0.0450078 |
| 839 | 5.09986 | 0.101838 | 0 | 0.020 | -1.47783 | 1.31429e-05 |
| 840 | 0.426473 | 0 | 0 | 0.000 | -0.126101 | 0.03653 |
| 847 | 1.91469 | 0 | 0 | 0.000 | -0.56614 | 0.000357851 |
| 848 | 0.791007 | 0 | 0 | 0.000 | -0.233887 | 0.0116285 |
| 850 | 1.32099 | 0.102282 | 0.129896 | 0.077 | -0.36035 | 0.0472734 |
| 851 | 1.891 | 0.105294 | 0 | 0.056 | -0.528002 | 0.00116723 |
| 854 | 8.63511 | 0.483932 | 0.215337 | 0.056 | -2.41016 | 0.00567296 |
| 863 | 8.83972 | 0.477123 | 0 | 0.054 | -2.47267 | 0.00544378 |
| 884 | 0.979481 | 0 | 0 | 0.000 | -0.289615 | 0.0013923 |
| 895 | 2.04006 | 0 | 0 | 0.000 | -0.603209 | 0.000238933 |
| 897 | 0.427376 | 0 | 0 | 0.000 | -0.126368 | 0.035029 |
| 899 | 1.2593 | 0.110931 | 0 | 0.088 | -0.339552 | 0.00811784 |
| 902 | 1.63131 | 0 | 0.286183 | 0.000 | -0.48235 | 0.0134109 |
| 903 | 168.992 | 0 | 0 | 0.000 | -49.968 | 0.000368319 |
| 923 | 1.06044 | 0 | 0 | 0.000 | -0.313555 | 0.00273846 |
| 928 | 1.93437 | 0.12594 | 0 | 0.065 | -0.53472 | 0.00925834 |
| 930 | 2.57672 | 0 | 0 | 0.000 | -0.761891 | 0.000443475 |
| 932 | 2.3242 | 0.236132 | 0 | 0.102 | -0.617406 | 0.0078177 |
| 935 | 1.44514 | 0.121188 | 0 | 0.084 | -0.391469 | 0.0241389 |
| 938 | 3.38344 | 0.263735 | 0 | 0.078 | -0.922441 | 0.0491223 |
| 939 | 1.03705 | 0 | 0 | 0.000 | -0.306637 | 0.00418459 |
| 940 | 14.6406 | 0.397427 | 0 | 0.027 | -4.21145 | 0.00505176 |
| 941 | 1.96335 | 0.139297 | 0 | 0.071 | -0.53934 | 0.0190957 |
| 947 | 1.89947 | 0.228462 | 0 | 0.120 | -0.494088 | 0.0129473 |
| 950 | 13.1021 | 0.436239 | 0.295016 | 0.033 | -3.74508 | 0.00571687 |
| 957 | 4.74165 | 0.286996 | 0.171612 | 0.061 | -1.31716 | 0.00172351 |
| 959 | 1.03825 | 0.140948 | 0.153643 | 0.136 | -0.265317 | 0.0415089 |
| 961 | 0.370067 | 0 | 0 | 0.000 | -0.109422 | 0.0353499 |
| 962 | 1.33876 | 0.118715 | 0.144182 | 0.089 | -0.360745 | 0.0345816 |
| 964 | 0.394212 | 0 | 0.135497 | 0.000 | -0.116562 | 0.0388377 |
| 965 | 0.873888 | 0 | 0 | 0.000 | -0.258393 | 0.00367559 |
| 966 | 0.554816 | 0 | 0.13702 | 0.000 | -0.164049 | 0.0236916 |
| 969 | 0.72219 | 0 | 0 | 0.000 | -0.213539 | 0.00667517 |
| 970 | 1.0147 | 0 | 0 | 0.000 | -0.300028 | 0.00187686 |
| 972 | 8.60301 | 0 | 0 | 0.000 | -2.54376 | 6.56331e-05 |
| 973 | 0.533921 | 0 | 0 | 0.000 | -0.157871 | 0.0224304 |
| 974 | 1.07114 | 0.10996 | 0 | 0.103 | -0.284203 | 0.0160072 |
| 975 | 1.75491 | 0.213367 | 0 | 0.122 | -0.455807 | 0.0127588 |
| 977 | 1.1102 | 0.126396 | 0 | 0.114 | -0.290893 | 0.0368906 |
| 978 | 2.49714 | 0.123878 | 0 | 0.050 | -0.701732 | 0.0044496 |
| 981 | 0.534733 | 0 | 0 | 0.000 | -0.158111 | 0.0174251 |
| 983 | 2.24777 | 0 | 0 | 0.000 | -0.664626 | 0.000334697 |
| 988 | 1.72897 | 0.0913346 | 0.300705 | 0.053 | -0.484221 | 0.00423489 |
| 989 | 2.19563 | 0 | 0 | 0.000 | -0.649208 | 0.000360916 |
| 991 | 1.3775 | 0.245176 | 0 | 0.178 | -0.334808 | 0.041543 |
| 992 | 1.60977 | 0 | 0 | 0.000 | -0.47598 | 0.00191869 |
| 995 | 1.95673 | 0.269613 | 0.154876 | 0.138 | -0.498852 | 0.0258344 |
| 996 | 2.38077 | 0 | 0 | 0.000 | -0.703951 | 0.000301606 |
| 997 | 0.814378 | 0 | 0 | 0.000 | -0.240797 | 0.00637565 |
| 998 | 55.9075 | 0 | 0 | 0.000 | -16.5309 | 9.19706e-06 |
| 999 | 1.15854 | 0 | 0 | 0.000 | -0.342561 | 0.00171473 |
| 1000 | 1.47285 | 0 | 0 | 0.000 | -0.435496 | 0.000845688 |
| 1001 | 2.80432 | 0 | 0 | 0.000 | -0.829189 | 1.5299e-05 |
| 1003 | 1.98005 | 0.112762 | 0 | 0.057 | -0.552124 | 0.00398089 |
| 1004 | 1.32472 | 0 | 0 | 0.000 | -0.391696 | 0.00140277 |
| 1007 | 0.48742 | 0 | 0 | 0.000 | -0.144122 | 0.0315994 |
| 1008 | 1.4695 | 0 | 0 | 0.000 | -0.434504 | 0.00126214 |
| 1009 | 0.825297 | 0.0993661 | 0.449179 | 0.120 | -0.214645 | 0.0471451 |
| 1010 | 2.22839 | 0 | 0 | 0.000 | -0.658897 | 0.000305706 |
| 1011 | 1.54891 | 0 | 0 | 0.000 | -0.457986 | 0.00136838 |
| 1012 | 3.1307 | 0.438467 | 0.171029 | 0.140 | -0.796046 | 0.0294445 |
| 1015 | 1.1859 | 0 | 0 | 0.000 | -0.35065 | 0.0049291 |
| 1016 | 25.2098 | 0.186901 | 0.555774 | 0.007 | -7.39882 | 0.00152278 |
| 1017 | 0.578775 | 0 | 0.13333 | 0.000 | -0.171134 | 0.0131143 |
| 1018 | 0.610458 | 0 | 0 | 0.000 | -0.180502 | 0.0241635 |
| 1019 | 2.92223 | 0 | 0 | 0.000 | -0.864053 | 5.3191e-05 |
| 1021 | 0.942576 | 0 | 0 | 0.000 | -0.278703 | 0.00163262 |
| 1022 | 1.46161 | 0 | 0 | 0.000 | -0.432172 | 0.00174485 |
| 1024 | 9.38753 | 0.20944 | 0 | 0.022 | -2.7138 | 0.00161501 |
| 1026 | 1.07575 | 0.109074 | 0.303084 | 0.101 | -0.28583 | 0.0147847 |
| 1027 | 2.61795 | 0 | 0 | 0.000 | -0.774081 | 7.34179e-05 |
| 1028 | 1.60414 | 0 | 0 | 0.000 | -0.474317 | 0.00060344 |
| 1029 | 3.42541 | 0 | 0 | 0.000 | -1.01283 | 6.62021e-05 |
| 1031 | 3.18241 | 0.351446 | 0.133708 | 0.110 | -0.837067 | 0.0316663 |
| 1032 | 9.84344 | 0.224205 | 0 | 0.023 | -2.84424 | 8.44483e-05 |
| 1038 | 5.24926 | 0.445689 | 0 | 0.085 | -1.42033 | 0.0110039 |
| 1039 | 2.20709 | 0.251757 | 0 | 0.114 | -0.578158 | 0.0104987 |
| 1040 | 5.98936 | 0 | 0 | 0.000 | -1.77095 | 3.36378e-07 |
| 1044 | 3.73705 | 0 | 0.164567 | 0.000 | -1.10498 | 1.60937e-05 |
| 1046 | 1.65703 | 0 | 0.477671 | 0.000 | -0.489955 | 0.000883939 |
| 1050 | 8.39837 | 0.263055 | 0.287529 | 0.031 | -2.40547 | 0.000678753 |
| 1052 | 0.639996 | 0 | 0 | 0.000 | -0.189236 | 0.0143106 |
| 1053 | 0.840823 | 0 | 0 | 0.000 | -0.248616 | 0.00544857 |
| 1059 | 7.63403 | 0.224203 | 0.20222 | 0.029 | -2.19095 | 0.0122126 |
| 1062 | 1.5392 | 0 | 0 | 0.000 | -0.455114 | 0.0109463 |
| 1074 | 3.06945 | 0.668004 | 0 | 0.218 | -0.710065 | 0.0391211 |
| 1075 | 1.84403 | 0.116047 | 0 | 0.063 | -0.510935 | 0.0309414 |
| 1077 | 3.51274 | 0 | 0 | 0.000 | -1.03865 | 2.22077e-05 |
| 1081 | 5.09886 | 0 | 0 | 0.000 | -1.50764 | 6.15802e-06 |
| 1082 | 1.97357 | 0 | 0 | 0.000 | -0.583551 | 4.3479e-05 |
| 1083 | 3.67872 | 0.420943 | 0.154644 | 0.114 | -0.963267 | 0.00762973 |
| 1095 | 2.92917 | 0.111204 | 0 | 0.038 | -0.833223 | 0.000233334 |
| 1096 | 3.93867 | 0 | 0 | 0.000 | -1.1646 | 2.7994e-05 |
| 1098 | 1.40959 | 0.101215 | 0.228701 | 0.072 | -0.386864 | 0.028132 |
| 1099 | 1.4151 | 0 | 0 | 0.000 | -0.41842 | 0.00511704 |
| 1100 | 7.62069 | 0 | 0.143113 | 0.000 | -2.25331 | 5.91361e-06 |
| 1101 | 0.692271 | 0 | 0 | 0.000 | -0.204693 | 0.011645 |
| 1102 | 1.98764 | 0 | 0 | 0.000 | -0.587709 | 0.000103857 |
| 1103 | 0.856186 | 0 | 0 | 0.000 | -0.253159 | 0.0041856 |
| 1104 | 2.97709 | 0.120254 | 0.134668 | 0.040 | -0.844715 | 0.0090064 |
| 1105 | 7.74002 | 0 | 0 | 0.000 | -2.28859 | 4.89495e-06 |
| 1106 | 0.936293 | 0 | 0 | 0.000 | -0.276845 | 0.00860863 |
| 1107 | 1.85395 | 0 | 0 | 0.000 | -0.548179 | 0.000160895 |
| 1108 | 4.09847 | 0 | 0 | 0.000 | -1.21185 | 1.27987e-05 |
| 1110 | 1.26831 | 0 | 0 | 0.000 | -0.375018 | 0.00145933 |
| 1111 | 8.3731 | 0 | 0 | 0.000 | -2.47578 | 2.7597e-08 |
| 1113 | 0.987579 | 0 | 0 | 0.000 | -0.29201 | 0.00560893 |
| 1114 | 1.04339 | 0 | 0 | 0.000 | -0.308511 | 0.00860985 |
| 1116 | 2.88381 | 0.105287 | 0 | 0.037 | -0.821561 | 0.00464999 |
| 1118 | 14.1414 | 0 | 0 | 0.000 | -4.18137 | 2.64231e-05 |
| 1119 | 2.55158 | 0 | 0 | 0.000 | -0.754457 | 0.000205576 |
| 1121 | 1.79472 | 0 | 0 | 0.000 | -0.530668 | 0.00176702 |
| 1122 | 1.70713 | 0.115721 | 0 | 0.068 | -0.470552 | 0.00458309 |
| 1124 | 1.20026 | 0 | 0 | 0.000 | -0.354896 | 0.00353742 |
| 1128 | 1.77726 | 0 | 0 | 0.000 | -0.525506 | 0.000437182 |
| 1129 | 0.868443 | 0 | 0 | 0.000 | -0.256783 | 0.00365737 |
| 1130 | 2.0712 | 0 | 0 | 0.000 | -0.612418 | 0.000143919 |
| 1131 | 1.89656 | 0.120048 | 0 | 0.063 | -0.525283 | 0.00222793 |
| 1133 | 1.2421 | 0 | 0 | 0.000 | -0.367268 | 0.000444279 |
| 1134 | 1.99324 | 0 | 0 | 0.000 | -0.589366 | 0.00103166 |
| 1135 | 0.99229 | 0 | 0 | 0.000 | -0.293403 | 0.0191768 |
| 1136 | 1.9289 | 0 | 0.164143 | 0.000 | -0.570342 | 0.000702702 |
| 1138 | 0.553391 | 0 | 0 | 0.000 | -0.163628 | 0.0121562 |
| 1141 | 4.76523 | 0 | 0 | 0.000 | -1.409 | 7.27487e-06 |
| 1142 | 2.15289 | 0 | 0 | 0.000 | -0.636571 | 0.000118166 |
| 1143 | 11.9762 | 0 | 0 | 0.000 | -3.54114 | 7.84276e-06 |
| 1144 | 0.501216 | 0 | 0 | 0.000 | -0.148201 | 0.0246531 |
| 1145 | 3.36684 | 0 | 0 | 0.000 | -0.995515 | 7.33589e-06 |
| 1147 | 2.41358 | 0.120267 | 0 | 0.050 | -0.678092 | 0.00725203 |
| 1150 | 1.80095 | 0 | 0 | 0.000 | -0.532511 | 0.000544441 |
| 1151 | 41.0519 | 0.0825243 | 0 | 0.002 | -12.1139 | 0.0237231 |
| 1154 | 0.766775 | 0 | 0 | 0.000 | -0.226722 | 0.00950566 |
| 1155 | 2.14588 | 0.111405 | 0 | 0.052 | -0.60156 | 0.00351063 |
| 1156 | 1.05188 | 0 | 0 | 0.000 | -0.311022 | 0.000833269 |
| 1157 | 12.6816 | 0 | 0 | 0.000 | -3.74972 | 0.000153721 |
| 1158 | 3.95086 | 0 | 0 | 0.000 | -1.1682 | 4.4674e-06 |
| 1160 | 1.68472 | 0 | 0 | 0.000 | -0.498142 | 0.00120062 |
| 1164 | 1.50335 | 0 | 0.345302 | 0.000 | -0.444513 | 0.00154095 |
| 1165 | 1.70328 | 0 | 0 | 0.000 | -0.503629 | 0.000364778 |
| 1167 | 1.1657 | 0.128208 | 0 | 0.110 | -0.306767 | 0.0268005 |
| 1172 | 1.68417 | 0.119254 | 0 | 0.071 | -0.462719 | 0.00287736 |
| 1173 | 2.50873 | 0.227504 | 0.288258 | 0.091 | -0.674518 | 0.00450855 |
| 1175 | 1.16697 | 0 | 0 | 0.000 | -0.345054 | 0.00128115 |
| 1176 | 1.34698 | 0 | 0 | 0.000 | -0.398279 | 0.00963072 |
| 1180 | 2.25617 | 0 | 0 | 0.000 | -0.667111 | 5.41301e-05 |
| 1181 | 10.7362 | 0 | 0 | 0.000 | -3.17451 | 8.41401e-07 |
| 1182 | 2.54065 | 0 | 0 | 0.000 | -0.751225 | 2.1773e-05 |
| 1183 | 2.03708 | 0 | 0 | 0.000 | -0.602329 | 0.000139836 |
| 1184 | 1.03631 | 0 | 0 | 0.000 | -0.306419 | 0.0018593 |
| 1185 | 3.1331 | 0 | 0 | 0.000 | -0.926402 | 0.000638054 |
| 1187 | 1.60196 | 0.129914 | 0 | 0.081 | -0.435259 | 0.02712 |
| 1188 | 1.11912 | 0 | 0 | 0.000 | -0.330903 | 0.00214078 |
| 1189 | 1.60135 | 0 | 0 | 0.000 | -0.473492 | 0.000467092 |
| 1190 | 2.13968 | 0 | 0 | 0.000 | -0.632665 | 0.00070916 |
| 1191 | 0.749955 | 0 | 0 | 0.000 | -0.221748 | 0.00671164 |
| 1192 | 1.03309 | 0 | 0 | 0.000 | -0.305467 | 0.0148433 |
| 1193 | 1.63045 | 0 | 0 | 0.000 | -0.482097 | 0.000428265 |
| 1194 | 1.12383 | 0 | 0 | 0.000 | -0.332297 | 0.00191915 |
| 1195 | 3.04467 | 0.229278 | 0 | 0.075 | -0.832461 | 0.0106875 |
| 1196 | 8.54258 | 0.412586 | 0.147222 | 0.048 | -2.4039 | 0.0328096 |
| 1197 | 0.466971 | 0 | 0 | 0.000 | -0.138075 | 0.018775 |
| 1198 | 0.570104 | 0 | 0 | 0.000 | -0.16857 | 0.0272958 |
| 1200 | 1.54275 | 0.139865 | 0.137718 | 0.091 | -0.414807 | 0.0207922 |
| 1201 | 0.700894 | 0 | 0 | 0.000 | -0.207242 | 0.00866941 |
| 1202 | 0.399449 | 0 | 0 | 0.000 | -0.11811 | 0.0411944 |
| 1204 | 1.87874 | 0.238983 | 0 | 0.127 | -0.484848 | 0.0150306 |
| 1205 | 1.79883 | 0 | 0 | 0.000 | -0.531881 | 0.000256412 |
| 1206 | 3.22161 | 0 | 0 | 0.000 | -0.952575 | 0.000141138 |
| 1208 | 0.86575 | 0 | 0 | 0.000 | -0.255987 | 0.00211084 |
| 1211 | 0.417246 | 0 | 0 | 0.000 | -0.123372 | 0.0236235 |
| 1212 | 1.70676 | 0 | 0 | 0.000 | -0.504658 | 0.000656786 |
| 1213 | 1.61122 | 0 | 0 | 0.000 | -0.476409 | 0.000104173 |
| 1215 | 1.09896 | 0 | 0 | 0.000 | -0.324943 | 0.00116576 |
| 1218 | 3.01254 | 0.219479 | 0 | 0.073 | -0.82586 | 0.00593112 |
| 1219 | 0.831268 | 0 | 0 | 0.000 | -0.245791 | 0.00618946 |
| 1222 | 0.764598 | 0 | 0.13792 | 0.000 | -0.226078 | 0.00355042 |
| 1229 | 2.61081 | 0.124284 | 0 | 0.048 | -0.735221 | 0.00214096 |
| 1230 | 1.9926 | 0.12209 | 0 | 0.061 | -0.553076 | 0.0072728 |
| 1234 | 1.2079 | 0 | 0 | 0.000 | -0.357154 | 0.00171781 |
| 1236 | 0.804987 | 0.102008 | 0 | 0.127 | -0.207859 | 0.0397627 |
| 1237 | 2.50584 | 0 | 0 | 0.000 | -0.740932 | 5.0794e-05 |
| 1239 | 2.23131 | 0 | 0 | 0.000 | -0.65976 | 0.000817898 |
| 1241 | 1.01337 | 0 | 0 | 0.000 | -0.299637 | 0.0097482 |
| 1243 | 0.694164 | 0 | 0 | 0.000 | -0.205252 | 0.0139656 |
| 1244 | 1.27479 | 0 | 0 | 0.000 | -0.376932 | 0.00119444 |
| 1245 | 1.44821 | 0 | 0 | 0.000 | -0.42821 | 0.000667037 |
| 1247 | 1.28678 | 0 | 0.147546 | 0.000 | -0.380478 | 0.00615041 |
| 1248 | 1.04004 | 0 | 0 | 0.000 | -0.307521 | 0.00372195 |
| 1249 | 4.42318 | 0.547221 | 0 | 0.124 | -1.14605 | 0.0220418 |
| 1250 | 0.842847 | 0 | 0 | 0.000 | -0.249215 | 0.00723383 |
| 1251 | 1.24338 | 0 | 0 | 0.000 | -0.367646 | 0.00247549 |
| 1254 | 1.11011 | 0 | 0 | 0.000 | -0.328242 | 0.0011456 |
| 1255 | 2.4077 | 0 | 0 | 0.000 | -0.711914 | 3.44281e-05 |
| 1258 | 0.88423 | 0 | 0 | 0.000 | -0.261451 | 0.00511557 |
| 1260 | 2.75428 | 0.223041 | 0 | 0.081 | -0.748442 | 0.0171496 |
| 1261 | 3.35196 | 0 | 0 | 0.000 | -0.991117 | 0.000276969 |
| 1262 | 8.53425 | 0 | 0 | 0.000 | -2.52343 | 1.08122e-05 |
| 1263 | 1.30985 | 0 | 0 | 0.000 | -0.3873 | 0.00254799 |
| 1264 | 0.59949 | 0 | 0 | 0.000 | -0.177259 | 0.00841824 |
| 1265 | 0.443231 | 0 | 0.146079 | 0.000 | -0.131056 | 0.0405001 |
| 1267 | 0.957217 | 0 | 0 | 0.000 | -0.283032 | 0.0139854 |
| 1268 | 1.20676 | 0 | 0 | 0.000 | -0.356818 | 0.000750239 |
| 1269 | 0.599302 | 0 | 0 | 0.000 | -0.177203 | 0.0246124 |
| 1272 | 0.620625 | 0 | 0 | 0.000 | -0.183508 | 0.0134648 |
| 1276 | 2.50454 | 0 | 0 | 0.000 | -0.740549 | 0.000130444 |
| 1277 | 5.11554 | 0.108342 | 0 | 0.021 | -1.48054 | 0.000297955 |
| 1278 | 6.36356 | 0.444947 | 0 | 0.070 | -1.75003 | 0.0169736 |
| 1279 | 2.87742 | 0.292598 | 0.183193 | 0.102 | -0.764285 | 0.0099812 |
| 1281 | 0.348574 | 0 | 0 | 0.000 | -0.103067 | 0.0476394 |
| 1282 | 0.653614 | 0 | 0 | 0.000 | -0.193262 | 0.0224503 |
| 1283 | 0.993646 | 0.105561 | 0 | 0.106 | -0.262591 | 0.0256872 |
| 1284 | 1.15289 | 0 | 0.145633 | 0.000 | -0.340889 | 0.00841127 |
| 1286 | 4.71204 | 0 | 0 | 0.000 | -1.39327 | 1.26373e-05 |
| 1287 | 2.21547 | 0 | 0 | 0.000 | -0.655075 | 0.000298854 |
| 1288 | 0.367975 | 0 | 0 | 0.000 | -0.108804 | 0.0345777 |
| 1289 | 1.07559 | 0 | 0 | 0.000 | -0.318033 | 0.00247409 |
| 1290 | 0.972642 | 0 | 0 | 0.000 | -0.287593 | 0.00205947 |
| 1291 | 5.78157 | 0 | 0 | 0.000 | -1.70951 | 1.94613e-06 |
| 1292 | 1.90714 | 0 | 0 | 0.000 | -0.563909 | 0.000160822 |
| 1293 | 2.51832 | 0 | 0 | 0.000 | -0.744624 | 5.45232e-05 |
| 1296 | 0.61544 | 0 | 0 | 0.000 | -0.181975 | 0.0114338 |
| 1298 | 1.2626 | 0 | 0 | 0.000 | -0.373329 | 0.000581508 |
| 1300 | 0.734385 | 0 | 0.158477 | 0.000 | -0.217145 | 0.00720864 |
| 1303 | 0.891318 | 0 | 0 | 0.000 | -0.263547 | 0.00315364 |
| 1304 | 1.43979 | 0 | 0 | 0.000 | -0.425722 | 0.00071407 |
| 1305 | 0.447785 | 0 | 0 | 0.000 | -0.132402 | 0.0188967 |
| 1308 | 1.59363 | 0.113389 | 0.272364 | 0.071 | -0.43768 | 0.0331709 |
| 1313 | 1.34219 | 0 | 0 | 0.000 | -0.396863 | 0.00167353 |
| 1314 | 1.49447 | 0 | 0 | 0.000 | -0.44189 | 0.000756469 |
| 1315 | 3.34158 | 0.112156 | 0 | 0.034 | -0.954885 | 0.00117549 |
| 1316 | 3.04542 | 0 | 0 | 0.000 | -0.900478 | 1.57441e-05 |
| 1317 | 1.00219 | 0 | 0.145369 | 0.000 | -0.29633 | 0.00134019 |
| 1320 | 2.48979 | 0 | 0 | 0.000 | -0.736189 | 0.000220819 |
| 1324 | 7.77787 | 0 | 0 | 0.000 | -2.29978 | 4.82343e-06 |
| 1325 | 0.887042 | 0 | 0 | 0.000 | -0.262283 | 0.00486795 |
| 1326 | 2.21884 | 0.120727 | 0 | 0.054 | -0.620375 | 0.00231728 |
| 1327 | 0.818609 | 0 | 0 | 0.000 | -0.242048 | 0.00579705 |
| 1328 | 18.0234 | 0 | 0 | 0.000 | -5.3292 | 4.02e-07 |
| 1329 | 1.83313 | 0 | 0 | 0.000 | -0.542025 | 0.000128301 |
| 1331 | 1.19466 | 0 | 0 | 0.000 | -0.353241 | 0.00334684 |
| 1332 | 9.37625 | 0.120629 | 0 | 0.013 | -2.73673 | 0.000475993 |
| 1333 | 0.443088 | 0 | 0 | 0.000 | -0.131013 | 0.0461549 |
| 1334 | 0.432684 | 0 | 0 | 0.000 | -0.127937 | 0.0210311 |
| 1337 | 1.03743 | 0 | 0 | 0.000 | -0.306749 | 0.00223712 |
| 1339 | 1.03511 | 0 | 0 | 0.000 | -0.306064 | 0.00243114 |
| 1340 | 1.55986 | 0.222412 | 0 | 0.143 | -0.395461 | 0.0164271 |
| 1341 | 0.516394 | 0 | 0 | 0.000 | -0.152689 | 0.0132302 |
| 1342 | 1.22254 | 0.164415 | 0 | 0.134 | -0.312869 | 0.0428216 |
| 1343 | 0.691283 | 0 | 0 | 0.000 | -0.2044 | 0.00973352 |
| 1344 | 1.41002 | 0 | 0 | 0.000 | -0.416919 | 0.000518123 |
| 1345 | 2.79303 | 0 | 0 | 0.000 | -0.82585 | 2.82365e-05 |
| 1348 | 1.08078 | 0.108411 | 0 | 0.100 | -0.287514 | 0.0211489 |
| 1350 | 0.501505 | 0 | 0 | 0.000 | -0.148286 | 0.0250762 |
| 1353 | 5.18283 | 0.180438 | 0.188159 | 0.035 | -1.47912 | 0.00287951 |
| 1357 | 1.83152 | 0 | 0 | 0.000 | -0.541548 | 6.52152e-05 |
| 1358 | 0.816289 | 0 | 0.135113 | 0.000 | -0.241362 | 0.00382852 |
| 1359 | 2.17796 | 0 | 0 | 0.000 | -0.643986 | 5.46246e-05 |
| 1360 | 2.55694 | 0 | 0 | 0.000 | -0.756043 | 2.80322e-05 |
| 1362 | 1.21454 | 0 | 0 | 0.000 | -0.359118 | 0.000335964 |
| 1363 | 1.80816 | 0.235513 | 0 | 0.130 | -0.465004 | 0.0101264 |
| 1367 | 2.52931 | 0.366092 | 0.146807 | 0.145 | -0.639625 | 0.0348732 |
| 1368 | 1.04134 | 0 | 0 | 0.000 | -0.307906 | 0.00169287 |
| 1369 | 1.30547 | 0.230911 | 0.27615 | 0.177 | -0.317728 | 0.035964 |
| 1370 | 1.88036 | 0 | 0 | 0.000 | -0.55599 | 5.34782e-05 |
| 1373 | 1.69927 | 0 | 0 | 0.000 | -0.502445 | 0.00026167 |
| 1376 | 3.04665 | 0.141164 | 0.310307 | 0.046 | -0.859101 | 0.00707911 |
| 1382 | 2.2928 | 0.225743 | 0 | 0.098 | -0.611193 | 0.00963576 |
| 1383 | 10.3792 | 0.398594 | 0.181805 | 0.038 | -2.95108 | 0.0491465 |
| 1387 | 1.35742 | 0.154135 | 0 | 0.114 | -0.355791 | 0.0381851 |
| 1388 | 6.02627 | 0.109019 | 0 | 0.018 | -1.74963 | 0.000714068 |
| 1389 | 2.32261 | 0.108188 | 0 | 0.047 | -0.654767 | 0.0413632 |
| 1390 | 7.37245 | 0.397668 | 0 | 0.054 | -2.06232 | 0.00392521 |
| 1394 | 2.08515 | 0.158092 | 0 | 0.076 | -0.569797 | 0.0139683 |
| 1395 | 1.78893 | 0 | 0 | 0.000 | -0.528955 | 0.00137706 |
| 1397 | 2.42356 | 0.225791 | 0.138377 | 0.093 | -0.649843 | 0.00652332 |
| 1410 | 2.44648 | 0.106688 | 0 | 0.044 | -0.691836 | 0.00443914 |
| 1411 | 1.55215 | 0.0919394 | 0 | 0.059 | -0.431758 | 0.0013368 |
| 1412 | 0.895739 | 0 | 0 | 0.000 | -0.264854 | 0.00629187 |
| 1415 | 3.30661 | 0.125641 | 0 | 0.038 | -0.940556 | 0.00718122 |
| 1417 | 1.01835 | 0 | 0 | 0.000 | -0.301108 | 0.0036991 |
| 1418 | 0.76767 | 0 | 0 | 0.000 | -0.226986 | 0.00893382 |
| 1420 | 0.978221 | 0.101733 | 0.131248 | 0.104 | -0.259162 | 0.0258713 |
| 1423 | 3.49606 | 0.453329 | 0.150392 | 0.130 | -0.899683 | 0.0351666 |
| 1425 | 1.57376 | 0 | 0 | 0.000 | -0.465333 | 0.00129991 |
| 1426 | 2.12067 | 0 | 0 | 0.000 | -0.627045 | 0.000119928 |
| 1427 | 1.20265 | 0 | 0.151247 | 0.000 | -0.355604 | 0.00870207 |
| 1428 | 1.99757 | 0 | 0.142134 | 0.000 | -0.590648 | 0.000789062 |
| 1431 | 8.2806 | 0 | 0 | 0.000 | -2.44843 | 0.0012698 |
| 1436 | 1.19064 | 0.132945 | 0 | 0.112 | -0.312741 | 0.0322374 |
| 1437 | 0.533009 | 0 | 0 | 0.000 | -0.157601 | 0.0261622 |
| 1444 | 1.02356 | 0.0964813 | 0 | 0.094 | -0.274122 | 0.0370151 |
| 1446 | 1.4704 | 0.108963 | 0 | 0.074 | -0.402552 | 0.0144376 |
| 1449 | 0.635266 | 0 | 0 | 0.000 | -0.187837 | 0.0215325 |
| 1450 | 2.61184 | 0 | 0 | 0.000 | -0.772276 | 0.00087603 |
| 1451 | 1.28362 | 0.107736 | 0 | 0.084 | -0.34769 | 0.0163282 |
| 1452 | 1.69427 | 0 | 0 | 0.000 | -0.500966 | 0.000414105 |
| 1455 | 1.87452 | 0.114076 | 0.310944 | 0.061 | -0.520532 | 0.00481369 |
| 1456 | 0.698446 | 0 | 0 | 0.000 | -0.206518 | 0.00876062 |
| 1459 | 3.78989 | 0.107791 | 0 | 0.028 | -1.08873 | 0.000614784 |
| 1461 | 0.961905 | 0 | 0 | 0.000 | -0.284418 | 0.00597622 |
| 1466 | 0.681599 | 0 | 0.125276 | 0.000 | -0.201537 | 0.00514214 |
| 1467 | 1.43344 | 0 | 0 | 0.000 | -0.423843 | 0.00192316 |
| 1469 | 1.10519 | 0 | 0.132426 | 0.000 | -0.326786 | 0.0021506 |
| 1471 | 1.04256 | 0 | 0.118369 | 0.000 | -0.308266 | 0.00148843 |
| 1473 | 2.2703 | 0.137439 | 0.362469 | 0.061 | -0.630649 | 0.00550841 |
| 1477 | 2.34531 | 0 | 0.329576 | 0.000 | -0.693467 | 0.000157153 |
| 1480 | 6.30829 | 0 | 0 | 0.000 | -1.86525 | 5.77095e-05 |
| 1481 | 1.11205 | 0 | 0 | 0.000 | -0.328814 | 0.00338522 |
| 1483 | 0.448177 | 0 | 0 | 0.000 | -0.132518 | 0.0147846 |
| 1484 | 2.37094 | 0.278129 | 0 | 0.117 | -0.618806 | 0.0146693 |
| 1485 | 1.41413 | 0 | 0 | 0.000 | -0.418133 | 0.00168311 |
| 1486 | 2.15344 | 0 | 0 | 0.000 | -0.636735 | 9.01801e-05 |
| 1488 | 0.393369 | 0 | 0 | 0.000 | -0.116312 | 0.0393598 |
| 1490 | 1.11574 | 0 | 0 | 0.000 | -0.329904 | 0.00803007 |
| 1491 | 0.871879 | 0 | 0 | 0.000 | -0.257799 | 0.00571097 |
| 1494 | 2.11662 | 0 | 0 | 0.000 | -0.625847 | 0.000166344 |
| 1495 | 2.65089 | 0 | 0 | 0.000 | -0.78382 | 0.000189107 |
| 1496 | 11.144 | 0.543076 | 0.144904 | 0.049 | -3.13452 | 0.0053271 |
| 1497 | 1.95553 | 0 | 0 | 0.000 | -0.578215 | 0.000128052 |
| 1498 | 2.02853 | 0.124162 | 0 | 0.061 | -0.563088 | 0.00518312 |
| 1501 | 0.496668 | 0 | 0 | 0.000 | -0.146856 | 0.0208071 |
| 1504 | 2.40519 | 0.10316 | 0 | 0.043 | -0.68067 | 0.001058 |
| 1505 | 2.64352 | 0.364825 | 0 | 0.138 | -0.673769 | 0.0162097 |
| 1507 | 1.98539 | 0 | 0 | 0.000 | -0.587046 | 0.000884291 |
| 1508 | 2.37036 | 0 | 0 | 0.000 | -0.700874 | 2.28767e-05 |
| 1514 | 4.57064 | 0.102776 | 0 | 0.022 | -1.32107 | 0.000423395 |
| 1516 | 9.49355 | 0 | 0 | 0.000 | -2.80708 | 2.19837e-05 |
| 1517 | 4.0165 | 0.354917 | 0 | 0.088 | -1.08267 | 0.0123651 |
| 1518 | 1.01486 | 0 | 0 | 0.000 | -0.300078 | 0.00123975 |
| 1519 | 0.970084 | 0 | 0 | 0.000 | -0.286837 | 0.00370287 |
| 1520 | 0.434815 | 0 | 0 | 0.000 | -0.128567 | 0.0284965 |
| 1521 | 2.34494 | 0.238292 | 0 | 0.102 | -0.6229 | 0.024926 |
| 1523 | 3.93842 | 0 | 0.138903 | 0.000 | -1.16452 | 6.08945e-05 |
| 1526 | 0.448323 | 0 | 0 | 0.000 | -0.132561 | 0.0216931 |
| 1529 | 2.22872 | 0 | 0 | 0.000 | -0.658993 | 0.000815942 |
| 1530 | 0.434269 | 0 | 0 | 0.000 | -0.128406 | 0.0340153 |
| 1532 | 0.95938 | 0.120475 | 0 | 0.126 | -0.24805 | 0.0453461 |
| 1533 | 2.10349 | 0 | 0 | 0.000 | -0.621966 | 0.000545287 |
| 1535 | 1.35628 | 0 | 0 | 0.000 | -0.401028 | 0.00021544 |
| 1538 | 0.704233 | 0 | 0 | 0.000 | -0.208229 | 0.00761609 |
| 1542 | 1.88144 | 0 | 0 | 0.000 | -0.556308 | 0.000434018 |
| 1543 | 6.16743 | 0.289828 | 0 | 0.047 | -1.7379 | 0.00140595 |
| 1551 | 1.74482 | 0.0925398 | 0 | 0.053 | -0.48855 | 0.00183922 |
| 1553 | 0.722956 | 0 | 0 | 0.000 | -0.213765 | 0.0103701 |
| 1555 | 0.987447 | 0 | 0 | 0.000 | -0.291971 | 0.00276028 |
| 1556 | 1.12985 | 0 | 0 | 0.000 | -0.334077 | 0.00282307 |
| 1557 | 1.23421 | 0 | 0 | 0.000 | -0.364936 | 0.0041393 |
| 1560 | 0.955469 | 0 | 0 | 0.000 | -0.282515 | 0.00135968 |
| 1562 | 12.4503 | 0 | 0 | 0.000 | -3.68133 | 2.8988e-06 |
| 1564 | 1.00512 | 0 | 0 | 0.000 | -0.297196 | 0.00254456 |
| 1565 | 3.67683 | 0 | 0 | 0.000 | -1.08718 | 2.979e-05 |
| 1567 | 1.16758 | 0 | 0.168142 | 0.000 | -0.345233 | 0.00331075 |
| 1568 | 10.8925 | 0 | 0 | 0.000 | -3.22071 | 7.152e-06 |
| 1572 | 0.451218 | 0 | 0 | 0.000 | -0.133417 | 0.0354828 |
| 1576 | 4.61587 | 0.598457 | 0.144903 | 0.130 | -1.18788 | 0.0212696 |
| 1577 | 0.83217 | 0 | 0 | 0.000 | -0.246058 | 0.0182307 |
| 1583 | 2.66193 | 0.119969 | 0 | 0.045 | -0.751614 | 0.00244716 |
| 1588 | 5.84713 | 0 | 0 | 0.000 | -1.72889 | 3.20219e-05 |
| 1589 | 1.41199 | 0 | 0.13966 | 0.000 | -0.417502 | 0.00586109 |
| 1590 | 1.25952 | 0.0963543 | 0 | 0.077 | -0.343926 | 0.0102271 |
| 1592 | 6.1886 | 0 | 0 | 0.000 | -1.82986 | 1.60469e-05 |
| 1594 | 0.695465 | 0 | 0 | 0.000 | -0.205637 | 0.00818985 |
| 1595 | 1.96823 | 0 | 0 | 0.000 | -0.58197 | 0.000229938 |

**Table S11** Negative selection sites for the *S* gene based on REL analysis.

| Codon | E[dS] | E[dN] | Normalized E[dN-dS] | Posterior Probability | Bayes Factor |
| --- | --- | --- | --- | --- | --- |
| 8 | 1.22617 | 0.195445 | -1.03072 | 0.999928 | 878.098 |
| 11 | 1.28033 | 0.159032 | -1.1213 | 0.999978 | 2909.21 |
| 16 | 4.27218 | 0.688878 | -3.5833 | 0.999687 | 202.695 |
| 40 | 1.57328 | 0.200903 | -1.37237 | 0.999918 | 769.946 |
| 41 | 1.11714 | 0.200724 | -0.916414 | 0.999802 | 321.164 |
| 56 | 2.30103 | 0.230846 | -2.07018 | 0.999006 | 63.7928 |
| 71 | 0.974832 | 0.059175 | -0.915657 | 0.999985 | 4356.88 |
| 72 | 0.722996 | 0.0210149 | -0.701981 | 1 | 612381 |
| 73 | 0.892089 | 0.1402 | -0.751889 | 0.999961 | 1641.74 |
| 74 | 0.908141 | 0.0212416 | -0.886899 | 1 | 654539 |
| 75 | 1.20693 | 0.194828 | -1.01211 | 0.999941 | 1083.58 |
| 76 | 1.27049 | 0.0185793 | -1.25191 | 1 | 8.93272e+08 |
| 85 | 1.65415 | 0.0592416 | -1.59491 | 0.999717 | 224.004 |
| 89 | 2.11635 | 0.201802 | -1.91455 | 0.999883 | 540.462 |
| 114 | 1.74787 | 0.0429159 | -1.70495 | 0.999915 | 750.498 |
| 115 | 1.24792 | 0.202383 | -1.04554 | 0.99943 | 111.225 |
| 118 | 1.40086 | 0.13677 | -1.26408 | 0.999992 | 7721.02 |
| 119 | 1.58981 | 0.20312 | -1.38669 | 0.99958 | 150.914 |
| 120 | 1.06001 | 0.0512369 | -1.00878 | 0.999997 | 23345.7 |
| 122 | 1.36182 | 0.192536 | -1.16928 | 0.999962 | 1689.86 |
| 125 | 1.24243 | 0.203303 | -1.03913 | 0.998881 | 56.6838 |
| 137 | 4.38682 | 0.200482 | -4.18634 | 0.999994 | 10752.2 |
| 142 | 0.864203 | 0.154605 | -0.709598 | 0.999901 | 642.983 |
| 144 | 3.93329 | 0.228821 | -3.70447 | 0.99969 | 204.916 |
| 146 | 1.66002 | 0.20999 | -1.45003 | 0.999605 | 160.506 |
| 148 | 1.28541 | 0.202609 | -1.0828 | 0.999788 | 299.22 |
| 150 | 1.31231 | 0.199811 | -1.1125 | 0.999956 | 1437.84 |
| 178 | 1.27182 | 0.139105 | -1.13271 | 0.999992 | 7562.97 |
| 233 | 1.25083 | 0.049165 | -1.20166 | 0.999998 | 35650.8 |
| 235 | 1.76743 | 0.207615 | -1.55981 | 0.998952 | 60.525 |
| 241 | 1.02456 | 0.196106 | -0.82845 | 0.999585 | 152.797 |
| 243 | 1.3382 | 0.203609 | -1.13459 | 0.999912 | 724.162 |
| 244 | 1.29891 | 0.150306 | -1.14861 | 0.999977 | 2772.25 |
| 245 | 1.20153 | 0.173277 | -1.02826 | 0.99974 | 243.873 |
| 256 | 1.20799 | 0.106071 | -1.10192 | 0.999997 | 23184.7 |
| 262 | 1.4386 | 0.205651 | -1.23295 | 0.999394 | 104.694 |
| 263 | 1.01837 | 0.200373 | -0.818 | 0.999835 | 385.151 |
| 267 | 4.36375 | 0.684924 | -3.67883 | 0.9997 | 211.76 |
| 287 | 0.592194 | 0.0226754 | -0.569518 | 0.999999 | 71755.4 |
| 291 | 1.28523 | 0.0314339 | -1.2538 | 1 | 1.59504e+06 |
| 292 | 0.760964 | 0.0212428 | -0.739721 | 1 | 463194 |
| 293 | 0.720726 | 0.0212419 | -0.699484 | 1 | 442606 |
| 296 | 1.1544 | 0.111654 | -1.04275 | 0.999995 | 12271.7 |
| 298 | 0.669102 | 0.200562 | -0.46854 | 0.99926 | 85.7146 |
| 320 | 0.816873 | 0.0200813 | -0.796792 | 1 | 3.00857e+06 |
| 321 | 1.10056 | 0.203663 | -0.896901 | 0.998778 | 51.8627 |
| 323 | 1.73262 | 0.201569 | -1.53105 | 0.999937 | 1007.47 |
| 326 | 1.1401 | 0.0192969 | -1.1208 | 1 | 1.60181e+07 |
| 327 | 0.81996 | 0.19356 | -0.6264 | 0.999667 | 190.697 |
| 334 | 0.623487 | 0.0208633 | -0.602624 | 1 | 605075 |
| 337 | 0.782422 | 0.0185799 | -0.763842 | 1 | 9.94291e+07 |
| 340 | 1.20478 | 0.0191186 | -1.18566 | 1 | 2.05478e+07 |
| 341 | 0.983462 | 0.11667 | -0.866792 | 0.999993 | 8977.52 |
| 342 | 0.8745 | 0.0191501 | -0.85535 | 1 | 1.00477e+07 |
| 343 | 0.995687 | 0.0317527 | -0.963935 | 1 | 379617 |
| 344 | 0.838136 | 0.0193205 | -0.818815 | 1 | 6.94268e+06 |
| 346 | 0.990924 | 0.0191503 | -0.971774 | 1 | 1.2194e+07 |
| 348 | 0.689309 | 0.0218279 | -0.667481 | 0.999999 | 124164 |
| 349 | 0.781738 | 0.0385135 | -0.743224 | 0.999998 | 36563.9 |
| 352 | 0.908953 | 0.0196746 | -0.889279 | 1 | 4.30504e+06 |
| 353 | 2.4935 | 0.0310983 | -2.4624 | 1 | 1.17028e+07 |
| 357 | 0.732045 | 0.0211692 | -0.710875 | 1 | 507908 |
| 363 | 1.83557 | 0.020034 | -1.81554 | 1 | 5.09424e+07 |
| 364 | 0.758304 | 0.0185793 | -0.739724 | 1 | 9.45929e+07 |
| 365 | 1.08727 | 0.0637659 | -1.02351 | 0.99999 | 6211.42 |
| 366 | 1.04167 | 0.145057 | -0.896609 | 0.999957 | 1490.71 |
| 367 | 0.854635 | 0.0437805 | -0.810854 | 0.999997 | 19753 |
| 368 | 1.13499 | 0.190676 | -0.94431 | 0.999942 | 1094.08 |
| 369 | 1.80065 | 0.0200733 | -1.78057 | 1 | 4.27206e+07 |
| 370 | 1.76507 | 0.0534325 | -1.71164 | 0.999999 | 60471.9 |
| 373 | 0.961754 | 0.046082 | -0.915672 | 0.999997 | 20778 |
| 374 | 1.03268 | 0.0185793 | -1.0141 | 1 | 2.68608e+08 |
| 383 | 1.03098 | 0.202023 | -0.828953 | 0.998819 | 53.6719 |
| 385 | 0.770667 | 0.0191478 | -0.75152 | 1 | 7.99295e+06 |
| 388 | 2.00575 | 0.211799 | -1.79395 | 0.999057 | 67.2648 |
| 392 | 3.06855 | 0.212332 | -2.85622 | 0.999447 | 114.675 |
| 393 | 1.11412 | 0.0200785 | -1.09404 | 1 | 5.297e+06 |
| 399 | 1.20009 | 0.0607591 | -1.13933 | 0.99999 | 6579.29 |
| 401 | 0.918326 | 0.201414 | -0.716912 | 0.998775 | 51.7491 |
| 406 | 1.07194 | 0.019468 | -1.05248 | 1 | 1.95268e+07 |
| 407 | 0.858265 | 0.0191437 | -0.839122 | 1 | 1.03116e+07 |
| 408 | 1.14446 | 0.106885 | -1.03758 | 0.999995 | 12498.1 |
| 409 | 1.40757 | 0.128498 | -1.27907 | 0.999997 | 24948.1 |
| 411 | 1.32551 | 0.200443 | -1.12507 | 0.999892 | 589.181 |
| 412 | 1.19793 | 0.112139 | -1.0858 | 0.999996 | 15481.2 |
| 417 | 1.06216 | 0.202661 | -0.859502 | 0.999077 | 68.7223 |
| 426 | 0.821017 | 0.168729 | -0.652288 | 0.99932 | 93.3291 |
| 427 | 1.21365 | 0.201413 | -1.01223 | 0.999583 | 152.269 |
| 428 | 0.829297 | 0.200507 | -0.62879 | 0.999616 | 165.116 |
| 429 | 0.723967 | 0.0305159 | -0.693451 | 1 | 199281 |
| 436 | 1.11835 | 0.138881 | -0.979467 | 0.999944 | 1141.02 |
| 437 | 2.00841 | 0.207825 | -1.80059 | 0.999165 | 75.9517 |
| 439 | 1.57489 | 0.129312 | -1.44557 | 0.999995 | 13170.7 |
| 440 | 1.09522 | 0.0200792 | -1.07514 | 1 | 5.78181e+06 |
| 459 | 0.972151 | 0.202111 | -0.77004 | 0.998998 | 63.3074 |
| 460 | 0.833857 | 0.0242823 | -0.809575 | 1 | 2.87308e+06 |
| 461 | 0.825049 | 0.0316279 | -0.793421 | 1 | 191251 |
| 462 | 0.730506 | 0.0459767 | -0.684529 | 0.999997 | 21387.7 |
| 467 | 1.30146 | 0.18509 | -1.11637 | 0.999995 | 12490.9 |
| 468 | 1.39885 | 0.204834 | -1.19402 | 0.999641 | 176.609 |
| 470 | 0.83948 | 0.0200813 | -0.819399 | 1 | 2.96059e+06 |
| 477 | 1.89441 | 0.195197 | -1.69921 | 0.999968 | 2010.43 |
| 480 | 1.11298 | 0.185586 | -0.927396 | 0.999982 | 3597.33 |
| 483 | 1.44939 | 0.200395 | -1.249 | 0.999979 | 3046.5 |
| 486 | 1.5396 | 0.117127 | -1.42247 | 0.999996 | 14642.9 |
| 490 | 1.1913 | 0.0224222 | -1.16888 | 1 | 175853 |
| 491 | 0.544355 | 0.0185804 | -0.525775 | 1 | 6.32761e+07 |
| 492 | 0.777709 | 0.018582 | -0.759127 | 1 | 1.17541e+08 |
| 494 | 2.27808 | 0.0206567 | -2.25742 | 1 | 1.43827e+07 |
| 495 | 0.975937 | 0.201657 | -0.774279 | 0.999174 | 76.7609 |
| 496 | 1.00121 | 0.019504 | -0.98171 | 1 | 9.54891e+06 |
| 498 | 0.727135 | 0.0331722 | -0.693962 | 0.999999 | 124870 |
| 501 | 1.19635 | 0.0339567 | -1.16239 | 1 | 667926 |
| 503 | 0.584429 | 0.0210667 | -0.563362 | 1 | 430293 |
| 509 | 0.866967 | 0.0209131 | -0.846054 | 1 | 933855 |
| 513 | 1.06634 | 0.0315308 | -1.03481 | 1 | 388845 |
| 515 | 1.87162 | 0.196132 | -1.67549 | 0.999985 | 4098.02 |
| 521 | 0.895924 | 0.139078 | -0.756846 | 0.999928 | 883.882 |
| 523 | 1.02846 | 0.0320695 | -0.99639 | 1 | 266479 |
| 524 | 0.986751 | 0.119672 | -0.86708 | 0.99999 | 6049.44 |
| 526 | 0.849485 | 0.0333607 | -0.816124 | 0.999999 | 117096 |
| 536 | 1.02862 | 0.193669 | -0.834953 | 0.999853 | 431.703 |
| 537 | 1.15585 | 0.0302206 | -1.12562 | 1 | 560564 |
| 539 | 0.838581 | 0.0191312 | -0.819449 | 1 | 1.01921e+07 |
| 540 | 0.799651 | 0.129493 | -0.670158 | 0.999942 | 1086.72 |
| 541 | 1.12137 | 0.032177 | -1.08919 | 1 | 444090 |
| 542 | 1.03365 | 0.0514031 | -0.982246 | 0.999997 | 19597 |
| 543 | 0.674837 | 0.0191286 | -0.655708 | 1 | 6.66709e+06 |
| 552 | 0.70663 | 0.0206578 | -0.685972 | 1 | 470530 |
| 555 | 1.07477 | 0.0301852 | -1.04458 | 1 | 571514 |
| 557 | 0.926918 | 0.0196712 | -0.907247 | 1 | 3.06437e+06 |
| 558 | 1.31154 | 0.123607 | -1.18793 | 0.999997 | 20951.7 |
| 559 | 1.1765 | 0.192252 | -0.984244 | 0.999747 | 250.654 |
| 560 | 1.17585 | 0.19634 | -0.979511 | 0.999944 | 1138.35 |
| 563 | 0.974677 | 0.0200798 | -0.954597 | 1 | 4.04826e+06 |
| 568 | 0.903148 | 0.11184 | -0.791307 | 0.999991 | 6716.93 |
| 570 | 1.10967 | 0.0374262 | -1.07225 | 0.999999 | 110733 |
| 571 | 1.2045 | 0.0340924 | -1.17041 | 1 | 442108 |
| 572 | 1.08814 | 0.020079 | -1.06806 | 1 | 5.79823e+06 |
| 574 | 1.08058 | 0.0199342 | -1.06065 | 1 | 9.10663e+06 |
| 575 | 1.0199 | 0.195619 | -0.824283 | 0.999651 | 181.677 |
| 579 | 1.14993 | 0.0220526 | -1.12788 | 1 | 562837 |
| 582 | 1.20058 | 0.0212746 | -1.1793 | 1 | 4.97693e+06 |
| 583 | 0.95199 | 0.019152 | -0.932838 | 1 | 1.17322e+07 |
| 584 | 0.781585 | 0.021242 | -0.760343 | 1 | 485994 |
| 586 | 1.18898 | 0.137366 | -1.05162 | 0.999993 | 8499.42 |
| 594 | 1.44298 | 0.205845 | -1.23714 | 0.999508 | 128.953 |
| 596 | 1.03228 | 0.190713 | -0.841568 | 0.999932 | 933.243 |
| 601 | 1.08374 | 0.0206564 | -1.06308 | 1 | 1.78376e+06 |
| 603 | 0.991871 | 0.0468251 | -0.945046 | 0.999998 | 26569.9 |
| 604 | 0.733668 | 0.0185803 | -0.715088 | 1 | 9.52544e+07 |
| 606 | 1.30735 | 0.110719 | -1.19663 | 0.999991 | 6778.97 |
| 609 | 1.30312 | 0.0459964 | -1.25713 | 0.999998 | 35038 |
| 613 | 1.0777 | 0.0321504 | -1.04555 | 1 | 290062 |
| 615 | 1.45757 | 0.105937 | -1.35163 | 0.999999 | 100625 |
| 620 | 1.43933 | 0.201708 | -1.23762 | 0.999811 | 336.544 |
| 626 | 1.19746 | 0.202465 | -0.994993 | 0.999734 | 238.619 |
| 631 | 2.88984 | 0.241061 | -2.64878 | 0.998949 | 60.3522 |
| 647 | 1.7091 | 0.204351 | -1.50475 | 0.999692 | 206.333 |
| 663 | 1.1975 | 0.0200779 | -1.17742 | 1 | 6.71582e+06 |
| 668 | 1.10337 | 0.0380198 | -1.06535 | 1 | 289972 |
| 669 | 1.22038 | 0.0343183 | -1.18606 | 1 | 483943 |
| 670 | 0.924211 | 0.128272 | -0.795939 | 0.999972 | 2245.03 |
| 677 | 1.17844 | 0.0523702 | -1.12607 | 0.999998 | 33777.8 |
| 696 | 1.42742 | 0.209377 | -1.21804 | 0.99903 | 65.3578 |
| 705 | 1.21973 | 0.0200776 | -1.19966 | 1 | 1.07064e+07 |
| 706 | 0.668458 | 0.0185825 | -0.649876 | 1 | 9.46672e+07 |
| 707 | 0.643712 | 0.0190803 | -0.624632 | 1 | 8.14611e+06 |
| 708 | 0.938302 | 0.146725 | -0.791577 | 0.999923 | 828.76 |
| 710 | 1.0975 | 0.137001 | -0.960502 | 0.999982 | 3466.34 |
| 713 | 1.00825 | 0.0193079 | -0.988942 | 1 | 1.2782e+07 |
| 715 | 0.832437 | 0.0411846 | -0.791253 | 0.999998 | 42040.2 |
| 716 | 0.917258 | 0.141657 | -0.775601 | 0.999934 | 958.278 |
| 721 | 1.17973 | 0.203841 | -0.975885 | 0.998813 | 53.4194 |
| 727 | 0.756614 | 0.0746393 | -0.681975 | 0.999999 | 76614.6 |
| 732 | 0.861091 | 0.0200812 | -0.84101 | 1 | 3.34854e+06 |
| 736 | 1.124 | 0.0566801 | -1.06732 | 0.99999 | 6626.45 |
| 746 | 0.748829 | 0.025903 | -0.722926 | 0.999994 | 11065.3 |
| 751 | 1.10409 | 0.160358 | -0.943733 | 0.999845 | 408.499 |
| 752 | 1.13218 | 0.195579 | -0.936597 | 0.999815 | 343.113 |
| 753 | 0.922448 | 0.0479017 | -0.874546 | 0.999996 | 16268.1 |
| 755 | 1.00042 | 0.0511159 | -0.9493 | 0.999997 | 18807.3 |
| 761 | 0.963721 | 0.118718 | -0.845003 | 0.999982 | 3483.23 |
| 765 | 0.809691 | 0.0848954 | -0.724796 | 0.999998 | 27067.6 |
| 771 | 1.20149 | 0.203049 | -0.998445 | 0.99923 | 82.3273 |
| 773 | 1.23681 | 0.0195545 | -1.21726 | 1 | 1.20296e+07 |
| 774 | 1.14117 | 0.20266 | -0.93851 | 0.999589 | 154.232 |
| 775 | 0.858347 | 0.190982 | -0.667365 | 0.999854 | 433.677 |
| 777 | 0.908978 | 0.196982 | -0.711996 | 0.999438 | 112.823 |
| 782 | 1.32192 | 0.0200771 | -1.30184 | 1 | 1.24606e+07 |
| 783 | 0.646008 | 0.0191299 | -0.626878 | 1 | 6.47514e+06 |
| 785 | 1.06232 | 0.0212411 | -1.04108 | 1 | 1.11671e+06 |
| 787 | 0.83039 | 0.034582 | -0.795808 | 0.999999 | 56992.8 |
| 788 | 0.885553 | 0.0212425 | -0.86431 | 1 | 600415 |
| 789 | 0.958776 | 0.0211712 | -0.937605 | 1 | 942445 |
| 792 | 0.617088 | 0.0209767 | -0.596111 | 1 | 511099 |
| 794 | 0.631409 | 0.0208592 | -0.61055 | 1 | 631624 |
| 796 | 0.657276 | 0.0194943 | -0.637781 | 1 | 2.09743e+06 |
| 797 | 1.11202 | 0.0532221 | -1.0588 | 0.999995 | 12070.4 |
| 799 | 0.715321 | 0.0191292 | -0.696192 | 1 | 7.38157e+06 |
| 800 | 0.966807 | 0.0328932 | -0.933914 | 1 | 226822 |
| 802 | 3.47006 | 0.20522 | -3.26484 | 0.999879 | 523.954 |
| 806 | 0.544292 | 0.0208966 | -0.523395 | 1 | 507999 |
| 808 | 1.09805 | 0.0212411 | -1.0768 | 1 | 1.21754e+06 |
| 809 | 0.547042 | 0.021243 | -0.525799 | 1 | 323820 |
| 810 | 0.785726 | 0.0191332 | -0.766593 | 1 | 8.60999e+06 |
| 811 | 0.628152 | 0.0205725 | -0.60758 | 1 | 484396 |
| 814 | 0.630962 | 0.0209506 | -0.610012 | 1 | 528708 |
| 815 | 1.33962 | 0.124338 | -1.21528 | 0.999996 | 16679.2 |
| 816 | 1.1051 | 0.0204305 | -1.08467 | 1 | 942164 |
| 817 | 0.976505 | 0.021766 | -0.954739 | 0.999999 | 102067 |
| 818 | 0.956153 | 0.047143 | -0.90901 | 0.999996 | 17299.3 |
| 820 | 0.845073 | 0.0192421 | -0.82583 | 1 | 1.09154e+07 |
| 825 | 0.753452 | 0.0208677 | -0.732584 | 1 | 791738 |
| 830 | 1.08329 | 0.0420922 | -1.04119 | 0.999999 | 55594 |
| 832 | 0.6537 | 0.018581 | -0.635119 | 1 | 7.79527e+07 |
| 836 | 1.14636 | 0.120784 | -1.02557 | 0.999992 | 7508.48 |
| 837 | 0.687595 | 0.116476 | -0.571118 | 0.999982 | 3445 |
| 839 | 1.8015 | 0.0319484 | -1.76955 | 1 | 4.52711e+06 |
| 840 | 0.62256 | 0.019344 | -0.603216 | 1 | 3.91644e+06 |
| 847 | 1.0449 | 0.0206517 | -1.02425 | 1 | 1.20323e+06 |
| 848 | 0.704812 | 0.0213671 | -0.683444 | 1 | 375783 |
| 850 | 0.881499 | 0.102913 | -0.778586 | 0.999993 | 9397.04 |
| 851 | 1.07181 | 0.0320831 | -1.03972 | 1 | 437041 |
| 854 | 1.71742 | 0.20305 | -1.51437 | 0.999693 | 206.368 |
| 863 | 1.78566 | 0.200454 | -1.58521 | 0.999961 | 1623.32 |
| 884 | 0.730528 | 0.0185802 | -0.711948 | 1 | 9.4564e+07 |
| 895 | 1.0634 | 0.0191035 | -1.04429 | 1 | 2.02207e+07 |
| 897 | 0.612677 | 0.0212422 | -0.591435 | 1 | 362527 |
| 899 | 0.846045 | 0.0360093 | -0.810035 | 0.999999 | 82569.5 |
| 902 | 0.953389 | 0.139888 | -0.813501 | 0.999958 | 1503.72 |
| 903 | 1.48968 | 0.0352606 | -1.45442 | 0.999991 | 6894.32 |
| 923 | 0.87345 | 0.0200801 | -0.85337 | 1 | 3.25014e+06 |
| 928 | 1.03943 | 0.0429008 | -0.996527 | 0.999999 | 43457.1 |
| 930 | 1.12443 | 0.0212411 | -1.10319 | 1 | 1.24424e+06 |
| 932 | 1.14052 | 0.118875 | -1.02165 | 0.999995 | 12241.2 |
| 935 | 0.907042 | 0.0441003 | -0.862942 | 0.999997 | 25185.3 |
| 938 | 1.11626 | 0.128724 | -0.987534 | 0.999985 | 4193.19 |
| 939 | 0.816699 | 0.0209873 | -0.795712 | 1 | 769105 |
| 940 | 1.55516 | 0.192958 | -1.3622 | 0.999978 | 2887.7 |
| 941 | 1.0462 | 0.05654 | -0.989657 | 0.999941 | 1075.62 |
| 947 | 1.03593 | 0.112748 | -0.923185 | 0.999995 | 12310.6 |
| 950 | 1.72152 | 0.203127 | -1.51839 | 0.999491 | 124.683 |
| 957 | 1.50949 | 0.196698 | -1.3128 | 0.999953 | 1361.98 |
| 959 | 0.751109 | 0.149713 | -0.601396 | 0.999883 | 544.645 |
| 961 | 0.595059 | 0.0185826 | -0.576477 | 1 | 8.32223e+07 |
| 962 | 0.885036 | 0.128811 | -0.756224 | 0.999973 | 2330.76 |
| 964 | 0.604793 | 0.0303677 | -0.574425 | 1 | 136897 |
| 965 | 0.673093 | 0.0208569 | -0.652237 | 1 | 636124 |
| 966 | 0.647676 | 0.0317487 | -0.615927 | 1 | 128241 |
| 969 | 0.713132 | 0.0194556 | -0.693676 | 1 | 2.62817e+06 |
| 970 | 0.815282 | 0.0185822 | -0.7967 | 1 | 1.29502e+08 |
| 972 | 1.34569 | 0.0193132 | -1.32638 | 1 | 2.21028e+07 |
| 973 | 0.624144 | 0.0191068 | -0.605037 | 1 | 7.1487e+06 |
| 974 | 0.759376 | 0.033718 | -0.725658 | 0.999999 | 105043 |
| 975 | 1.002 | 0.112739 | -0.88926 | 0.999993 | 9068.26 |
| 977 | 0.800158 | 0.0474966 | -0.752661 | 0.999995 | 11719.7 |
| 978 | 1.11224 | 0.0453559 | -1.06688 | 0.999999 | 81405.2 |
| 981 | 0.586228 | 0.021243 | -0.564985 | 1 | 343841 |
| 983 | 1.05507 | 0.0200541 | -1.03501 | 1 | 7.4833e+06 |
| 988 | 0.980475 | 0.183789 | -0.796686 | 0.999971 | 2221.59 |
| 989 | 1.05047 | 0.0206549 | -1.02981 | 1 | 1.35751e+06 |
| 991 | 0.89905 | 0.140054 | -0.758996 | 0.999934 | 956.76 |
| 992 | 0.970405 | 0.0195934 | -0.950811 | 1 | 5.66394e+06 |
| 995 | 1.06373 | 0.194464 | -0.869264 | 0.999768 | 273.294 |
| 996 | 1.09337 | 0.0212412 | -1.07213 | 1 | 1.45584e+06 |
| 997 | 0.706946 | 0.020657 | -0.686289 | 1 | 470706 |
| 998 | 1.63135 | 0.0200742 | -1.61128 | 1 | 1.42079e+07 |
| 999 | 0.870697 | 0.0193101 | -0.851387 | 1 | 7.62744e+06 |
| 1000 | 0.929248 | 0.0209163 | -0.908332 | 1 | 1.23674e+06 |
| 1001 | 1.1978 | 0.0192884 | -1.17852 | 1 | 3.81906e+07 |
| 1003 | 1.06209 | 0.0373831 | -1.02471 | 0.999998 | 36480 |
| 1004 | 0.893128 | 0.020081 | -0.873047 | 1 | 3.59946e+06 |
| 1007 | 0.557291 | 0.0225314 | -0.53476 | 0.999999 | 83013.9 |
| 1008 | 0.921811 | 0.0223835 | -0.899428 | 0.999999 | 62099.3 |
| 1009 | 0.679638 | 0.200468 | -0.479169 | 0.999377 | 101.86 |
| 1010 | 1.07026 | 0.0199349 | -1.05033 | 1 | 8.83797e+06 |
| 1011 | 0.971073 | 0.021241 | -0.949832 | 1 | 791914 |
| 1012 | 1.20266 | 0.202353 | -1.00031 | 0.999535 | 136.345 |
| 1015 | 0.906507 | 0.0200803 | -0.886426 | 1 | 3.5051e+06 |
| 1016 | 1.54915 | 0.202025 | -1.34712 | 0.999788 | 299.779 |
| 1017 | 0.636834 | 0.0304064 | -0.606427 | 1 | 146055 |
| 1018 | 0.699177 | 0.0196187 | -0.679558 | 1 | 1.67706e+06 |
| 1019 | 1.16922 | 0.0195737 | -1.14965 | 1 | 1.60914e+07 |
| 1021 | 0.685367 | 0.0193427 | -0.666025 | 1 | 4.36154e+06 |
| 1022 | 0.923256 | 0.0200286 | -0.903227 | 1 | 468350 |
| 1024 | 1.58062 | 0.100858 | -1.47976 | 0.999996 | 14715.8 |
| 1026 | 0.730684 | 0.189999 | -0.540685 | 0.999796 | 310.634 |
| 1027 | 1.16045 | 0.0200255 | -1.14042 | 1 | 2.75086e+06 |
| 1028 | 0.968598 | 0.0199401 | -0.948658 | 1 | 1.41061e+06 |
| 1029 | 1.23925 | 0.0194083 | -1.21984 | 1 | 2.4417e+07 |
| 1031 | 1.1727 | 0.200598 | -0.972098 | 0.999848 | 417.8 |
| 1032 | 2.49675 | 0.109409 | -2.38734 | 1 | 150388 |
| 1038 | 1.5003 | 0.192898 | -1.3074 | 0.999946 | 1178.23 |
| 1039 | 1.09448 | 0.137329 | -0.957147 | 0.999979 | 3083.08 |
| 1040 | 2.20299 | 0.0203304 | -2.18266 | 1 | 4.59675e+07 |
| 1044 | 1.3179 | 0.0492919 | -1.2686 | 0.999999 | 78919.9 |
| 1046 | 1.01327 | 0.171981 | -0.841291 | 0.999301 | 90.7522 |
| 1050 | 2.00293 | 0.201081 | -1.80185 | 0.99995 | 1269.64 |
| 1052 | 0.645918 | 0.0190969 | -0.626821 | 1 | 7.68648e+06 |
| 1053 | 0.747361 | 0.0193007 | -0.72806 | 1 | 6.04763e+06 |
| 1059 | 1.35582 | 0.18403 | -1.17179 | 0.999427 | 110.781 |
| 1062 | 0.930747 | 0.0352639 | -0.895483 | 0.999977 | 2741.71 |
| 1074 | 1.19903 | 0.201968 | -0.997062 | 0.999758 | 262.223 |
| 1075 | 0.947975 | 0.0416483 | -0.906327 | 0.999998 | 30427 |
| 1077 | 1.32486 | 0.025361 | -1.2995 | 1 | 443145 |
| 1081 | 1.96035 | 0.0230006 | -1.93735 | 1 | 3.36967e+06 |
| 1082 | 1.05462 | 0.0185793 | -1.03604 | 1 | 3.04612e+08 |
| 1083 | 1.30292 | 0.202997 | -1.09992 | 0.999453 | 116.027 |
| 1095 | 1.19902 | 0.0391461 | -1.15987 | 1 | 153949 |
| 1096 | 1.30151 | 0.0195106 | -1.282 | 1 | 2.45292e+07 |
| 1098 | 0.893808 | 0.151326 | -0.742482 | 0.999924 | 831.037 |
| 1099 | 0.925979 | 0.0193542 | -0.906625 | 1 | 1.09726e+07 |
| 1100 | 1.69781 | 0.0346041 | -1.66321 | 1 | 891596 |
| 1101 | 0.594049 | 0.0226272 | -0.571422 | 0.999999 | 80275.5 |
| 1102 | 1.07119 | 0.020726 | -1.05046 | 1 | 1.02371e+06 |
| 1103 | 0.730613 | 0.0190979 | -0.711515 | 1 | 8.77685e+06 |
| 1104 | 1.19586 | 0.124059 | -1.0718 | 0.999992 | 7785.5 |
| 1105 | 1.75562 | 0.019537 | -1.73608 | 1 | 3.47251e+07 |
| 1106 | 0.810521 | 0.0206517 | -0.789869 | 1 | 577664 |
| 1107 | 1.04364 | 0.0206575 | -1.02299 | 1 | 1.43205e+06 |
| 1108 | 1.36186 | 0.0191312 | -1.34273 | 1 | 5.16307e+07 |
| 1110 | 0.864519 | 0.020948 | -0.843571 | 1 | 950350 |
| 1111 | 4.05537 | 0.0208621 | -4.03451 | 1 | 6.2087e+07 |
| 1113 | 0.780628 | 0.0191305 | -0.761498 | 1 | 8.64857e+06 |
| 1114 | 0.816677 | 0.0206558 | -0.796021 | 1 | 601864 |
| 1116 | 1.10997 | 0.0334357 | -1.07653 | 1 | 390662 |
| 1118 | 1.77417 | 0.0212391 | -1.75293 | 1 | 2.51319e+06 |
| 1119 | 1.11586 | 0.0194115 | -1.09645 | 1 | 1.50143e+07 |
| 1121 | 1.00474 | 0.0200796 | -0.984658 | 1 | 4.74729e+06 |
| 1122 | 1.00677 | 0.0383478 | -0.968421 | 0.999999 | 78626 |
| 1124 | 0.872329 | 0.0208997 | -0.851429 | 1 | 1.01392e+06 |
| 1128 | 1.00559 | 0.0193437 | -0.986251 | 1 | 1.03047e+07 |
| 1129 | 0.711204 | 0.019493 | -0.691711 | 1 | 4.09252e+06 |
| 1130 | 1.10105 | 0.022442 | -1.0786 | 1 | 145389 |
| 1131 | 1.07162 | 0.0448438 | -1.02678 | 0.999998 | 40718.7 |
| 1133 | 0.841687 | 0.019343 | -0.822344 | 1 | 6.26298e+06 |
| 1134 | 1.01081 | 0.0198727 | -0.990933 | 1 | 8.14032e+06 |
| 1135 | 0.874836 | 0.0212425 | -0.853593 | 1 | 589365 |
| 1136 | 1.01011 | 0.0491954 | -0.96092 | 0.999998 | 30235.4 |
| 1138 | 0.551606 | 0.0208559 | -0.53075 | 1 | 553157 |
| 1141 | 1.48497 | 0.0206564 | -1.46431 | 1 | 6.43393e+06 |
| 1142 | 1.11835 | 0.0225979 | -1.09575 | 1 | 353539 |
| 1143 | 1.4914 | 0.0185799 | -1.47282 | 1 | 6.62569e+08 |
| 1144 | 0.597507 | 0.0208577 | -0.57665 | 1 | 595444 |
| 1145 | 1.28005 | 0.020655 | -1.2594 | 1 | 7.79647e+06 |
| 1147 | 1.09192 | 0.0386869 | -1.05324 | 1 | 134288 |
| 1150 | 0.983973 | 0.0195461 | -0.964427 | 1 | 7.83298e+06 |
| 1151 | 1.24556 | 0.0227366 | -1.22282 | 1 | 1.30328e+07 |
| 1154 | 0.769204 | 0.0212416 | -0.747963 | 1 | 490059 |
| 1155 | 1.09249 | 0.0346444 | -1.05785 | 1 | 190716 |
| 1156 | 0.749207 | 0.0193428 | -0.729864 | 1 | 4.97418e+06 |
| 1157 | 1.27788 | 0.0194639 | -1.25842 | 1 | 1.32689e+07 |
| 1158 | 1.47763 | 0.0207126 | -1.45692 | 1 | 1.59257e+06 |
| 1160 | 0.98049 | 0.0210065 | -0.959483 | 1 | 1.16211e+06 |
| 1164 | 0.931013 | 0.1544 | -0.776613 | 0.999767 | 271.814 |
| 1165 | 1.00045 | 0.0210337 | -0.979415 | 1 | 1.31455e+06 |
| 1167 | 0.824356 | 0.0516472 | -0.772709 | 0.999988 | 5204.7 |
| 1172 | 1.02164 | 0.0442156 | -0.977424 | 0.999999 | 46101.1 |
| 1173 | 1.13825 | 0.200527 | -0.937721 | 0.999897 | 614.355 |
| 1175 | 0.84425 | 0.0185818 | -0.825668 | 1 | 1.39553e+08 |
| 1176 | 0.933009 | 0.019146 | -0.913863 | 1 | 1.17076e+07 |
| 1180 | 1.11966 | 0.0206539 | -1.09901 | 1 | 2.62414e+06 |
| 1181 | 2.19841 | 0.020118 | -2.1783 | 1 | 5.60294e+07 |
| 1182 | 1.1723 | 0.0193424 | -1.15296 | 1 | 2.83682e+07 |
| 1183 | 1.06384 | 0.0199574 | -1.04388 | 1 | 4.43667e+06 |
| 1184 | 0.759812 | 0.0198971 | -0.739915 | 1 | 1.05812e+06 |
| 1185 | 1.14099 | 0.0193397 | -1.12165 | 1 | 1.46971e+07 |
| 1187 | 0.954851 | 0.0491055 | -0.905745 | 0.999996 | 15070.7 |
| 1188 | 0.815612 | 0.0193424 | -0.796269 | 1 | 5.9614e+06 |
| 1189 | 1.00129 | 0.0220861 | -0.979207 | 1 | 171642 |
| 1190 | 1.03835 | 0.0199665 | -1.01838 | 1 | 7.43238e+06 |
| 1191 | 0.676132 | 0.0191252 | -0.657007 | 1 | 6.59533e+06 |
| 1192 | 0.928548 | 0.0196111 | -0.908937 | 1 | 4.59571e+06 |
| 1193 | 0.976669 | 0.0206586 | -0.956011 | 1 | 1.04041e+06 |
| 1194 | 0.763861 | 0.0226214 | -0.741239 | 0.999999 | 88553.7 |
| 1195 | 1.14796 | 0.131026 | -1.01694 | 0.999993 | 8985.7 |
| 1196 | 1.53122 | 0.202357 | -1.32887 | 0.999374 | 101.291 |
| 1197 | 0.603561 | 0.019209 | -0.584352 | 1 | 6.33973e+06 |
| 1198 | 0.641938 | 0.0203994 | -0.621538 | 1 | 1.71076e+06 |
| 1200 | 0.942026 | 0.141519 | -0.800507 | 0.999962 | 1672.87 |
| 1201 | 0.589687 | 0.0210376 | -0.56865 | 1 | 203539 |
| 1202 | 0.648267 | 0.0193436 | -0.628923 | 1 | 4.21911e+06 |
| 1204 | 1.03084 | 0.12689 | -0.90395 | 0.999985 | 4357.37 |
| 1205 | 0.983506 | 0.0190709 | -0.964435 | 1 | 2.06111e+07 |
| 1206 | 1.21178 | 0.0193284 | -1.19245 | 1 | 1.73879e+07 |
| 1208 | 0.68645 | 0.019344 | -0.667106 | 1 | 4.54905e+06 |
| 1211 | 0.581594 | 0.0191994 | -0.562395 | 1 | 6.41144e+06 |
| 1212 | 0.983908 | 0.0196998 | -0.964208 | 1 | 3.17105e+06 |
| 1213 | 0.989908 | 0.0191266 | -0.970781 | 1 | 1.64931e+07 |
| 1215 | 0.78303 | 0.0193427 | -0.763687 | 1 | 5.4478e+06 |
| 1218 | 1.16055 | 0.113568 | -1.04698 | 0.999997 | 22940.1 |
| 1219 | 0.7306 | 0.0206594 | -0.709941 | 1 | 495822 |
| 1222 | 0.660538 | 0.0320748 | -0.628463 | 0.999999 | 112546 |
| 1229 | 1.14281 | 0.0477125 | -1.0951 | 0.999999 | 54326.5 |
| 1230 | 1.03126 | 0.0442825 | -0.986978 | 0.999998 | 31784.1 |
| 1234 | 0.849362 | 0.0193438 | -0.830019 | 1 | 6.5325e+06 |
| 1236 | 0.64975 | 0.0320377 | -0.617712 | 0.999999 | 109407 |
| 1237 | 1.16095 | 0.0222809 | -1.13867 | 1 | 137764 |
| 1239 | 1.06237 | 0.0192609 | -1.04311 | 1 | 1.78106e+07 |
| 1241 | 0.811892 | 0.0198877 | -0.792005 | 1 | 4.42225e+06 |
| 1243 | 0.711615 | 0.0206571 | -0.690958 | 1 | 472716 |
| 1244 | 0.870935 | 0.0206627 | -0.850273 | 1 | 712263 |
| 1245 | 0.924088 | 0.0192878 | -0.9048 | 1 | 1.01187e+07 |
| 1247 | 0.874253 | 0.0378217 | -0.836432 | 1 | 138617 |
| 1248 | 0.802344 | 0.0208792 | -0.781464 | 1 | 865437 |
| 1249 | 1.34245 | 0.200999 | -1.14145 | 0.999889 | 571.65 |
| 1250 | 0.742171 | 0.0193426 | -0.722828 | 1 | 5.00047e+06 |
| 1251 | 0.836118 | 0.022106 | -0.814012 | 0.999999 | 49345.8 |
| 1254 | 0.776702 | 0.0201261 | -0.756575 | 1 | 1.0195e+06 |
| 1255 | 1.11833 | 0.0191292 | -1.0992 | 1 | 3.42326e+07 |
| 1258 | 0.655577 | 0.0225653 | -0.633011 | 0.999999 | 77450.4 |
| 1260 | 1.12045 | 0.113054 | -1.00739 | 0.999995 | 13889.5 |
| 1261 | 1.16141 | 0.019337 | -1.14208 | 1 | 1.48569e+07 |
| 1262 | 1.47688 | 0.0190788 | -1.4578 | 1 | 6.54437e+07 |
| 1263 | 0.873782 | 0.0200442 | -0.853738 | 1 | 3.85028e+06 |
| 1264 | 0.590236 | 0.0193433 | -0.570892 | 1 | 3.78456e+06 |
| 1265 | 0.642911 | 0.037057 | -0.605854 | 0.999998 | 30907.5 |
| 1267 | 0.909727 | 0.0191047 | -0.890623 | 1 | 1.89147e+07 |
| 1268 | 0.845592 | 0.019313 | -0.826279 | 1 | 8.65814e+06 |
| 1269 | 0.698343 | 0.0196116 | -0.678731 | 1 | 1.83581e+06 |
| 1272 | 0.647922 | 0.0197486 | -0.628173 | 1 | 1.47749e+06 |
| 1276 | 1.10819 | 0.0210447 | -1.08714 | 1 | 577779 |
| 1277 | 1.47698 | 0.034716 | -1.44227 | 1 | 702599 |
| 1278 | 1.42148 | 0.19521 | -1.22627 | 0.99994 | 1066.31 |
| 1279 | 1.18444 | 0.197253 | -0.987184 | 0.999772 | 278.102 |
| 1281 | 0.589583 | 0.0193439 | -0.570239 | 1 | 3.82526e+06 |
| 1282 | 0.772366 | 0.0195755 | -0.752791 | 1 | 3.83804e+06 |
| 1283 | 0.737192 | 0.0331591 | -0.704033 | 0.999999 | 126090 |
| 1284 | 0.859032 | 0.036527 | -0.822505 | 1 | 158073 |
| 1286 | 1.41744 | 0.0195325 | -1.39791 | 1 | 2.99267e+07 |
| 1287 | 1.06117 | 0.019531 | -1.04164 | 1 | 8.98717e+06 |
| 1288 | 0.532624 | 0.019826 | -0.512798 | 1 | 1.47053e+06 |
| 1289 | 0.819697 | 0.0196784 | -0.800018 | 1 | 2.06263e+06 |
| 1290 | 0.755816 | 0.0191288 | -0.736687 | 1 | 7.85241e+06 |
| 1291 | 1.82209 | 0.0210966 | -1.80099 | 1 | 1.60596e+07 |
| 1292 | 1.03148 | 0.0195157 | -1.01196 | 1 | 1.51665e+07 |
| 1293 | 1.149 | 0.021076 | -1.12793 | 1 | 798517 |
| 1296 | 0.646603 | 0.0192162 | -0.627387 | 1 | 7.9936e+06 |
| 1298 | 0.84989 | 0.0193429 | -0.830547 | 1 | 6.57111e+06 |
| 1300 | 0.612083 | 0.0441313 | -0.567952 | 0.999994 | 10753.8 |
| 1303 | 0.749984 | 0.0191293 | -0.730855 | 1 | 7.95371e+06 |
| 1304 | 0.928961 | 0.0218498 | -0.907111 | 1 | 165991 |
| 1305 | 0.551391 | 0.0191292 | -0.532262 | 1 | 5.57716e+06 |
| 1308 | 0.98505 | 0.187357 | -0.797693 | 0.999948 | 1229.43 |
| 1313 | 0.964525 | 0.0190667 | -0.945458 | 1 | 2.42808e+07 |
| 1314 | 0.923798 | 0.0195119 | -0.904286 | 1 | 9.24378e+06 |
| 1315 | 1.23323 | 0.0375857 | -1.19565 | 1 | 402373 |
| 1316 | 1.22369 | 0.0201231 | -1.20357 | 1 | 3.75454e+06 |
| 1317 | 0.731671 | 0.0337895 | -0.697882 | 0.999999 | 82183.6 |
| 1320 | 1.09386 | 0.0194886 | -1.07437 | 1 | 1.30412e+07 |
| 1324 | 1.81852 | 0.0200729 | -1.79844 | 1 | 2.84113e+07 |
| 1325 | 0.702293 | 0.0206535 | -0.681639 | 1 | 456265 |
| 1326 | 1.10537 | 0.0416341 | -1.06373 | 0.999999 | 79372 |
| 1327 | 0.683323 | 0.0206393 | -0.662684 | 1 | 552949 |
| 1328 | 2.52809 | 0.0200816 | -2.508 | 1 | 6.41049e+07 |
| 1329 | 1.03507 | 0.0195821 | -1.01549 | 1 | 9.34833e+06 |
| 1331 | 0.819328 | 0.0197828 | -0.799545 | 1 | 1.67701e+06 |
| 1332 | 1.66736 | 0.0402246 | -1.62713 | 1 | 185958 |
| 1333 | 0.577652 | 0.0215899 | -0.556062 | 1 | 234871 |
| 1334 | 0.557483 | 0.0190462 | -0.538437 | 1 | 7.86905e+06 |
| 1337 | 0.7718 | 0.0210494 | -0.750751 | 1 | 631647 |
| 1339 | 0.764612 | 0.0210776 | -0.743535 | 1 | 607818 |
| 1340 | 0.966118 | 0.109413 | -0.856705 | 0.999995 | 11899 |
| 1341 | 0.689981 | 0.018635 | -0.671346 | 1 | 8.63155e+07 |
| 1342 | 0.824524 | 0.0726269 | -0.751897 | 0.999948 | 1229.89 |
| 1343 | 0.697876 | 0.0190867 | -0.678789 | 1 | 8.4909e+06 |
| 1344 | 0.910651 | 0.0203664 | -0.890285 | 1 | 1.2011e+06 |
| 1345 | 1.17978 | 0.0198588 | -1.15992 | 1 | 5.15909e+06 |
| 1348 | 0.776684 | 0.0343131 | -0.742371 | 0.999999 | 84499.5 |
| 1350 | 0.638172 | 0.0185816 | -0.619591 | 1 | 8.44584e+07 |
| 1353 | 1.3564 | 0.188292 | -1.16811 | 0.999988 | 5344.31 |
| 1357 | 1.03888 | 0.0186545 | -1.02023 | 1 | 2.0142e+08 |
| 1358 | 0.707943 | 0.0305266 | -0.677416 | 1 | 145678 |
| 1359 | 1.11579 | 0.0206602 | -1.09513 | 1 | 2.50173e+06 |
| 1360 | 1.1793 | 0.0224761 | -1.15682 | 1 | 837631 |
| 1362 | 0.828795 | 0.0185794 | -0.810216 | 1 | 1.18506e+08 |
| 1363 | 1.0535 | 0.116388 | -0.93711 | 0.999995 | 11545.8 |
| 1367 | 1.10241 | 0.200823 | -0.901583 | 0.99978 | 288.817 |
| 1368 | 0.721586 | 0.0206539 | -0.700933 | 1 | 471855 |
| 1369 | 0.866569 | 0.2004 | -0.666169 | 0.999719 | 225.588 |
| 1370 | 1.06339 | 0.0193429 | -1.04405 | 1 | 1.6676e+07 |
| 1373 | 1.0058 | 0.0210217 | -0.984781 | 1 | 1.44958e+06 |
| 1376 | 1.18448 | 0.195058 | -0.989421 | 0.999888 | 567.526 |
| 1382 | 1.09397 | 0.126485 | -0.967488 | 0.999989 | 5601.83 |
| 1383 | 1.23897 | 0.202435 | -1.03653 | 0.99892 | 58.713 |
| 1387 | 0.898625 | 0.062244 | -0.836381 | 0.999974 | 2410.73 |
| 1388 | 1.66789 | 0.0384608 | -1.62943 | 1 | 156787 |
| 1389 | 1.01104 | 0.0352338 | -0.975808 | 1 | 169402 |
| 1390 | 1.54504 | 0.192819 | -1.35222 | 0.999978 | 2942.55 |
| 1394 | 1.09399 | 0.0704361 | -1.02356 | 0.999984 | 3854.54 |
| 1395 | 1.00353 | 0.0191267 | -0.9844 | 1 | 1.52347e+07 |
| 1397 | 1.17181 | 0.188063 | -0.983743 | 0.999975 | 2587.48 |
| 1410 | 1.10446 | 0.0326776 | -1.07179 | 1 | 297887 |
| 1411 | 0.976012 | 0.0252422 | -0.95077 | 1 | 3.87453e+06 |
| 1412 | 0.718398 | 0.0201039 | -0.698294 | 1 | 1.6517e+06 |
| 1415 | 1.17351 | 0.0473053 | -1.12621 | 0.999998 | 27984.2 |
| 1417 | 0.808855 | 0.0185815 | -0.790273 | 1 | 1.24888e+08 |
| 1418 | 0.697059 | 0.0202931 | -0.676766 | 1 | 1.04412e+06 |
| 1420 | 0.725476 | 0.10294 | -0.622536 | 0.999987 | 4823.51 |
| 1423 | 1.25698 | 0.202059 | -1.05493 | 0.999555 | 142.683 |
| 1425 | 0.966595 | 0.0191253 | -0.94747 | 1 | 1.36518e+07 |
| 1426 | 1.09539 | 0.0206516 | -1.07474 | 1 | 1.79456e+06 |
| 1427 | 0.897831 | 0.0372508 | -0.860581 | 1 | 130645 |
| 1428 | 1.02002 | 0.0347112 | -0.985313 | 0.999999 | 106537 |
| 1431 | 1.14266 | 0.0200786 | -1.12258 | 1 | 5.3984e+06 |
| 1436 | 0.82068 | 0.0532117 | -0.767468 | 0.999987 | 4763.03 |
| 1437 | 0.683006 | 0.0193263 | -0.66368 | 1 | 5.15706e+06 |
| 1444 | 0.822061 | 0.0279714 | -0.79409 | 1 | 594099 |
| 1446 | 0.922752 | 0.0355915 | -0.88716 | 0.999999 | 79974.7 |
| 1449 | 0.686772 | 0.019549 | -0.667223 | 1 | 3.51488e+06 |
| 1450 | 1.14231 | 0.0212406 | -1.12107 | 1 | 1.06497e+06 |
| 1451 | 0.89252 | 0.0358887 | -0.856632 | 0.999999 | 68066.1 |
| 1452 | 0.987056 | 0.0195326 | -0.967523 | 1 | 7.80462e+06 |
| 1455 | 1.03043 | 0.19168 | -0.838746 | 0.999907 | 685.235 |
| 1456 | 0.677763 | 0.0191294 | -0.658634 | 1 | 6.71129e+06 |
| 1459 | 1.28011 | 0.0347427 | -1.24537 | 1 | 534846 |
| 1461 | 0.736693 | 0.021607 | -0.715086 | 0.999999 | 116134 |
| 1466 | 0.699177 | 0.0252513 | -0.673926 | 1 | 1.38156e+06 |
| 1467 | 0.951197 | 0.0193238 | -0.931873 | 1 | 8.82087e+06 |
| 1469 | 0.827865 | 0.0297671 | -0.798098 | 1 | 266252 |
| 1471 | 0.788025 | 0.0242808 | -0.763745 | 1 | 2.6925e+06 |
| 1473 | 1.12112 | 0.197572 | -0.923551 | 0.999571 | 148.044 |
| 1477 | 1.10573 | 0.136685 | -0.969047 | 0.999955 | 1418.53 |
| 1480 | 1.34604 | 0.0190779 | -1.32696 | 1 | 3.43237e+07 |
| 1481 | 0.842513 | 0.0193063 | -0.823207 | 1 | 7.58629e+06 |
| 1483 | 0.54424 | 0.019128 | -0.525112 | 1 | 5.41168e+06 |
| 1484 | 1.1173 | 0.148548 | -0.968749 | 0.999977 | 2777.28 |
| 1485 | 0.908239 | 0.021468 | -0.886771 | 1 | 132465 |
| 1486 | 1.11708 | 0.0192906 | -1.09779 | 1 | 1.99686e+07 |
| 1488 | 0.624834 | 0.0191293 | -0.605705 | 1 | 6.24966e+06 |
| 1490 | 0.847971 | 0.0194003 | -0.828571 | 1 | 7.86118e+06 |
| 1491 | 0.724099 | 0.0196127 | -0.704486 | 1 | 2.01276e+06 |
| 1494 | 1.09778 | 0.022464 | -1.07532 | 1 | 311069 |
| 1495 | 1.14064 | 0.0194614 | -1.12118 | 1 | 1.53189e+07 |
| 1496 | 1.89783 | 0.203224 | -1.69461 | 0.999645 | 178.983 |
| 1497 | 1.08542 | 0.0199016 | -1.06552 | 1 | 2.79693e+06 |
| 1498 | 1.04346 | 0.044035 | -0.999428 | 0.999998 | 41765.4 |
| 1501 | 0.541783 | 0.021091 | -0.520692 | 1 | 181578 |
| 1504 | 1.13052 | 0.0319209 | -1.0986 | 1 | 543285 |
| 1505 | 1.2291 | 0.19287 | -1.03623 | 0.999955 | 1415.85 |
| 1507 | 1.01428 | 0.0193852 | -0.994892 | 1 | 1.25577e+07 |
| 1508 | 1.16021 | 0.0205473 | -1.13966 | 1 | 1.3178e+06 |
| 1514 | 1.36063 | 0.0322768 | -1.32835 | 1 | 633534 |
| 1516 | 1.38561 | 0.0193028 | -1.36631 | 1 | 3.0906e+07 |
| 1517 | 1.29652 | 0.193714 | -1.10281 | 0.999903 | 651.907 |
| 1518 | 0.761535 | 0.0191304 | -0.742405 | 1 | 7.92237e+06 |
| 1519 | 0.73314 | 0.0204369 | -0.712703 | 1 | 435003 |
| 1520 | 0.547157 | 0.0206607 | -0.526496 | 1 | 359111 |
| 1521 | 1.08164 | 0.12747 | -0.954174 | 0.999989 | 5922.69 |
| 1523 | 1.28751 | 0.0325918 | -1.25491 | 1 | 713039 |
| 1526 | 0.646143 | 0.0192284 | -0.626914 | 1 | 6.51985e+06 |
| 1529 | 1.04981 | 0.0193702 | -1.03044 | 1 | 1.42574e+07 |
| 1530 | 0.630849 | 0.0191287 | -0.61172 | 1 | 6.26436e+06 |
| 1532 | 0.729058 | 0.0448606 | -0.684198 | 0.999995 | 14017.5 |
| 1533 | 1.03338 | 0.0193028 | -1.01408 | 1 | 1.58111e+07 |
| 1535 | 0.890546 | 0.0185791 | -0.871967 | 1 | 1.45351e+08 |
| 1538 | 0.592831 | 0.0206518 | -0.572179 | 1 | 373637 |
| 1542 | 1.00272 | 0.0195414 | -0.983181 | 1 | 5.21206e+06 |
| 1543 | 1.62549 | 0.152843 | -1.47265 | 0.999992 | 8257.62 |
| 1551 | 1.00023 | 0.025402 | -0.974826 | 1 | 4.0252e+06 |
| 1553 | 0.596332 | 0.0225178 | -0.573815 | 0.999999 | 79965.9 |
| 1555 | 0.858647 | 0.0190928 | -0.839554 | 1 | 1.152e+07 |
| 1556 | 0.83485 | 0.02008 | -0.81477 | 1 | 3.15805e+06 |
| 1557 | 0.902297 | 0.0200809 | -0.882216 | 1 | 3.50275e+06 |
| 1560 | 0.709327 | 0.0191272 | -0.6902 | 1 | 7.01342e+06 |
| 1562 | 1.89882 | 0.0200723 | -1.87875 | 1 | 3.13385e+07 |
| 1564 | 0.789928 | 0.0212782 | -0.76865 | 1 | 512299 |
| 1565 | 1.29157 | 0.0200763 | -1.27149 | 1 | 1.56696e+07 |
| 1567 | 0.856031 | 0.047592 | -0.808439 | 0.999997 | 22576.3 |
| 1568 | 2.13244 | 0.0200711 | -2.11237 | 1 | 2.28771e+07 |
| 1572 | 0.638274 | 0.0200821 | -0.618192 | 1 | 2.06819e+06 |
| 1576 | 1.43387 | 0.204075 | -1.2298 | 0.999326 | 94.1696 |
| 1577 | 0.859406 | 0.0200808 | -0.839325 | 1 | 3.13071e+06 |
| 1583 | 1.15146 | 0.042544 | -1.10892 | 0.999998 | 30230.9 |
| 1588 | 1.50049 | 0.0212396 | -1.47925 | 1 | 3.21426e+06 |
| 1589 | 0.919541 | 0.0323916 | -0.887149 | 1 | 264819 |
| 1590 | 0.850217 | 0.0263678 | -0.823849 | 1 | 1.48371e+06 |
| 1592 | 1.57361 | 0.0193904 | -1.55422 | 1 | 4.47007e+07 |
| 1594 | 0.591553 | 0.0206539 | -0.570899 | 1 | 370356 |
| 1595 | 1.09384 | 0.0186606 | -1.07518 | 1 | 1.75969e+08 |
